# Multifunctional and Reprogrammable Magnetoactive Graphene Oxide Origami

**DOI:** 10.1002/advs.202514597

**Published:** 2025-10-30

**Authors:** Jun Cai, Yiwen Chen, Alireza Seyedkanani, Guocheng Shen, Marta Cerruti, Abdolhamid Akbarzadeh

**Affiliations:** ^1^ Department of Bioresource Engineering McGill University Montreal QC H9X 3V9 Canada; ^2^ Department of Mining and Materials Engineering McGill University Montreal QC H9A 0C3 Canada; ^3^ Department of Mechanical Engineering McGill University Montreal QC H3A 0C3 Canada

**Keywords:** magnetic graphene oxide, magnetization reprogramming, on‐demand manufacturing, sensoriactuator, soft robot

## Abstract

Magnetoactive materials, which change shape in response to magnetic fields, hold significant potential for applications in soft robotics, biomedical devices, and morphable structures. However, existing systems often suffer from complex fabrication processes, limited geometric customizability, and inefficient magnetization reprogramming strategies, especially for 3D structures. Here, lightweight magnetic graphene oxide (MGO) bilayer films incorporating hard‐magnetic microparticles are introduced to enable fast, precise, and stable shape‐morphing under magnetic actuation, including in aqueous environments. The paper‐like nature of MGO films allows low‐cost and straightforward fabrication of customized structures through post‐processing steps such as cutting, folding, and assembly. In addition, the hygroscopic properties of GO introduce a humidity‐tunable actuation, offering an extra degree of control. To address the reprogramming challenge, a reversible, high‐throughput, and energy‐efficient strategy is introduced based on the rearrangements of reusable MGO magnetic stickers, enabling multimodal magnetic shape reconfiguration and functional versatility. Their applications are showcased in in situ mechanical state transitions, sequential logic computing, and soft robot locomotion. Finally, a MGO sensoriactuator is demonstrated capable of magnetic actuation and real‐time deformation monitoring, paving the way for closed‐loop soft robotic systems. This work presents a sustainable, reconfigurable, and multifunctional strategy for advancing next‐generation intelligent magnetoactive soft machines.

## Graphical abstract

A magnetic graphene oxide film is developed that enables rapid, precise, and programmable shape morphing under magnetic actuation. The MGO sensoriactuators feature humidity‐tunable performance and integrated sensing–actuation capability. Customized origami structures can be easily created by simple cutting and folding, while reusable magnetic stickers allow energy‐efficient reprogramming for adaptive soft robots and smart devices.

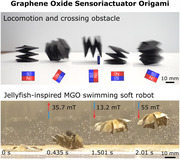

## 1 Introduction

Responsive actuators are able to convert various forms of energy into mechanical work,^[^ ^1^, ^2^ ^]^ enabling shape morphing in response to external stimuli such as light,^[^ ^3^ ^]^ temperature change,^[^ ^4^, ^5^, ^6^, ^7^ ^]^ pH,^[^ ^8^ ^]^ humidity,^[^ ^2^, ^5^ ^]^ electric fields,^[^ ^9^, ^10^ ^]^ air pressure,^[^ ^11^, ^12^, ^13^, ^14^, ^15^ ^]^ and magnetic fields.^[^ ^16^, ^17^, ^18^, ^19^, ^20^, ^21^, ^22^, ^23^, ^24^ ^]^ Among these, magnetic actuation offers a fast, safe, contactless, and precise control over responsive behaviors, making it highly promising for applications in robotics,^[^ ^17^, ^20^, ^24^, ^25^ ^]^ biomedical devices,^[^ ^26^, ^27^, ^28^ ^]^ soft manipulators,^[^ ^29^, ^30^ ^]^ and flexible electronics.^[^ ^31^ ^]^ Despite their versatility, most current magnetoactive materials/structures rely on fabrication techniques such as molding and casting,^[^ ^32^, ^33^, ^34^ ^]^ extrusion‐based three‐dimensional (3D) printing,^[^ ^20^, ^35^, ^36^ ^]^ microfabrication and assembly.^[^ ^21^, ^37^, ^38^ ^]^ These approaches generally require predefined molds or complex printing procedures, which are time‐consuming and limit the geometric flexibility, structural complexity, and scalability of magnetoactive machines. For example, modeling and casting are typically suited for two‐dimensional (2D) geometries and are not suitable for achieving complex 3D structures.^[^ ^39^, ^40^ ^]^ Extrusion‐based 3D printing, while offering design freedom, often faces challenges due to the viscosity and die swell of composite inks,^[^ ^40^, ^41^ ^]^ whereas light‐based 3D printing methods may encounter difficulties in removing supports, hindering the production of complex structures. These limitations underscore the need for simpler, faster, and more adaptable fabrication strategies for magnetoactive soft machines.

Recent advances in four‐dimensional (4D) printing have significantly expanded the field of magnetic materials. Magnetic particles, such as iron oxide, embedded within 3D‐printed polymer matrices can generate internal heat under alternating magnetic fields, thereby activating shape memory behavior and enabling remote actuation.^[^ ^42^, ^43^, ^44^, ^45^ ^]^ While 4D‐printed magnetic composites offer promising capabilities for remote actuation^[^ ^45^ ^]^ and enable complex structural transformations,^[^ ^43^ ^]^ their reliance on thermally driven activation poses challenges for temperature‐sensitive applications or systems with strict thermal management constraints.

Another crucial feature of magnetoactive systems is their capacity for post‐fabrication reprogramming of magnetization. Reprogrammability, i.e., the ability to reconfigure and alter functionalities as needed, is critical for magnetoactive machines to enable multifunctional operation and adaptation to dynamic environments. Various strategies have been proposed to achieve reprogramming of magnetic configurations.^[^ ^46^, ^47^, ^48^, ^49^, ^50^, ^51^, ^52^, ^53^ ^]^ The most straightforward approach involves heating magnetic samples above the Curie temperature to demagnetize them, followed by remagnetization under an external magnetic field during cooling.^[^ ^53^ ^]^ However, this method is typically applied to magnetic materials with relatively low Curie temperature (e.g., CrO_2_),^[^ ^53^ ^]^ and is incompatible with high‐remanence magnetic materials (e.g., NdFeB, SmCo).^[^ ^50^ ^]^ Another approach for magnetization reprogramming involves physically rotating the direction of magnetic particles in polymers under a magnetic field during a solid‐to‐liquid phase transition or through cleavage of dynamic linkages at elevated temperatures.^[^ ^46^, ^47^, ^49^ ^]^ While this method can reprogram high‐remanence magnetic materials, it demands significant energy input and remains difficult to apply to complex 3D structures. An alternative reprogramming strategy involves assembly and disassembly of multiple magnetic modules through magnetic attraction and electrostatic anchoring,^[^ ^54^, ^55^, ^56^ ^]^ but the assembly is prone to mechanical instabilities and external perturbations.^[^ ^50^, ^57^ ^]^ Therefore, a more convenient, flexible, and precise reprogramming strategy with minimal equipment and energy consumption is needed, particularly for reprogramming the magnetization of 3D structures.

Magnetic materials are commonly made by embedding magnetic microparticles into matrices such as hydrogels,^[^ ^28^, ^58^ ^]^ silicone elastomers,^[^ ^16^, ^25^, ^32^ ^]^ or other soft materials.^[^ ^30^, ^36^, ^39^, ^46^ ^]^ Graphene is an ideal matrix to fabricate lightweight magnetic soft materials due to its flexibility, large specific surface area‐to‐mass ratio, and excellent mechanical properties.^[^ ^59^, ^60^, ^61^ ^]^ Its water soluble and oxidized derivative, i.e., graphene oxide (GO), retains many of graphene's properties while offering easier processing and chemical versatility.^[^ ^61^, ^62^, ^63^ ^]^ Previous studies have shown that GO‐based bilayer films^[^ ^2^, ^3^ ^]^ can spontaneously deform under the actuation of light, heat, or humidity through asymmetric swelling or shrinkage caused by water molecule absorption/desorption, resulting in interfacial strain mismatch and bending deformation.^[^ ^2^ ^]^ However, these GO‐based actuators often suffer from slow response speeds,^[^ ^1^, ^2^, ^64^ ^]^ poor control precision,^[^ ^2^ ^]^ limited deformation modes, incompatibility with aqueous environments,^[^ ^65^ ^]^ and stimulus‐induced degradation of mechanical or electrical properties.^[^ ^62^ ^]^ We recently reported the production of flexible yet strong GO‐based papers with improved mechanical properties and water stability, which can be used to create various complex 3D structures through cutting, origami folding, and assembly.^[^ ^66^ ^]^ These GO films could be loaded with magnetically responsive components to develop lightweight magnetically‐responsive GO‐based metamaterials with complex architectures through origami engineering.^[^ ^67^, ^68^, ^69^, ^70^ ^]^

While magnetically actuated origami and soft robotic systems have been reported previously using polymer or elastomer matrices,^[^ ^16^, ^17^, ^24^, ^32^, ^71^ ^]^ there has been no demonstration of freestanding GO‐based magnetic films that combine lightweight paper‐like handling with high remanence hard‐magnetic particles. This unique material platform is important because GO not only provides mechanical robustness and origami foldability but also introduces multifunctionality not accessible to polymeric composites. For example, the electrical conductivity of GO/reduced GO is sensitive to mechanical deformation,^[^ ^72^, ^73^, ^74^ ^]^ suggesting future opportunities for building integrated sensoriactuators where actuation and sensing are coupled in a single platform. In addition, the lightweight nature of GO means that, for the same absolute amount of NdFeB particles, the weight fraction relative to the GO matrix is significantly higher than that relative to conventional polymer matrices, enhancing actuation efficiency. Together, these characteristics establish GO as a distinctive platform for magnetoactive systems that cannot be achieved with polymer‐ and elastomer‐based approaches.

Herein, we introduce a GO‐based magnetic material, termed magnetic GO (MGO), which consists of one GO layer and one composite layer containing dispersed hard‐magnetic microparticles and GO/poly(acrylic acid) (PAA). The density of the MGO film is ≈8.94 mg cm^−2^. The structures fabricated using MGO films can be precisely and remotely actuated by a magnetic field, delivering various functionalities, and outperforming current humidity‐responsive GO‐based actuators.^[^ ^2^, ^3^ ^]^ All magnetoactive machines developed in this study weigh less than one gram. Different from conventional fabrication approaches for magnetoactive machines, the paper‐like nature of MGO enables effortless creation of diverse designs through post‐fabrication steps such as cutting and origami folding. This capability offers the freedom to obtain complex structures and enables the realization of various functionalities and complex maneuverability, including multimodal locomotion, inchworm‐inspired walking, and jellyfish‐like swimming. Moreover, the hygroscopic nature of GO provides an additional control modality: environmental humidity can modulate the mechanical properties of the films and influence magnetic actuation performance, introducing a previously unexplored degree of control in magnetoactive systems. To address the reprogramming challenge, we introduce a reusable magnetic‐sticker‐assisted strategy that enables on‐demand programming and reprogramming of magnetization in 3D structures, without heating, solvent processing, or external anchoring. This method is low‐energy, reversible, and compatible with complex geometries, offering a practical alternative to conventional reprogramming techniques.^[^ ^46^, ^47^, ^53^, ^54^ ^]^ Finally, by integrating a strain‐sensitive GO layer, we realize self‐sensing MGO actuators capable of simultaneous deformation and real‐time feedback. Together, these features establish a scalable, reconfigurable, and sustainable platform for next‐generation intelligent magnetoactive soft machines with potential in soft robotics, underwater devices, and adaptive biomedical systems.

## 2 Results and Discussion

### 2.1 Fabrication and Characterization of MGO Films

**Figure 1:**
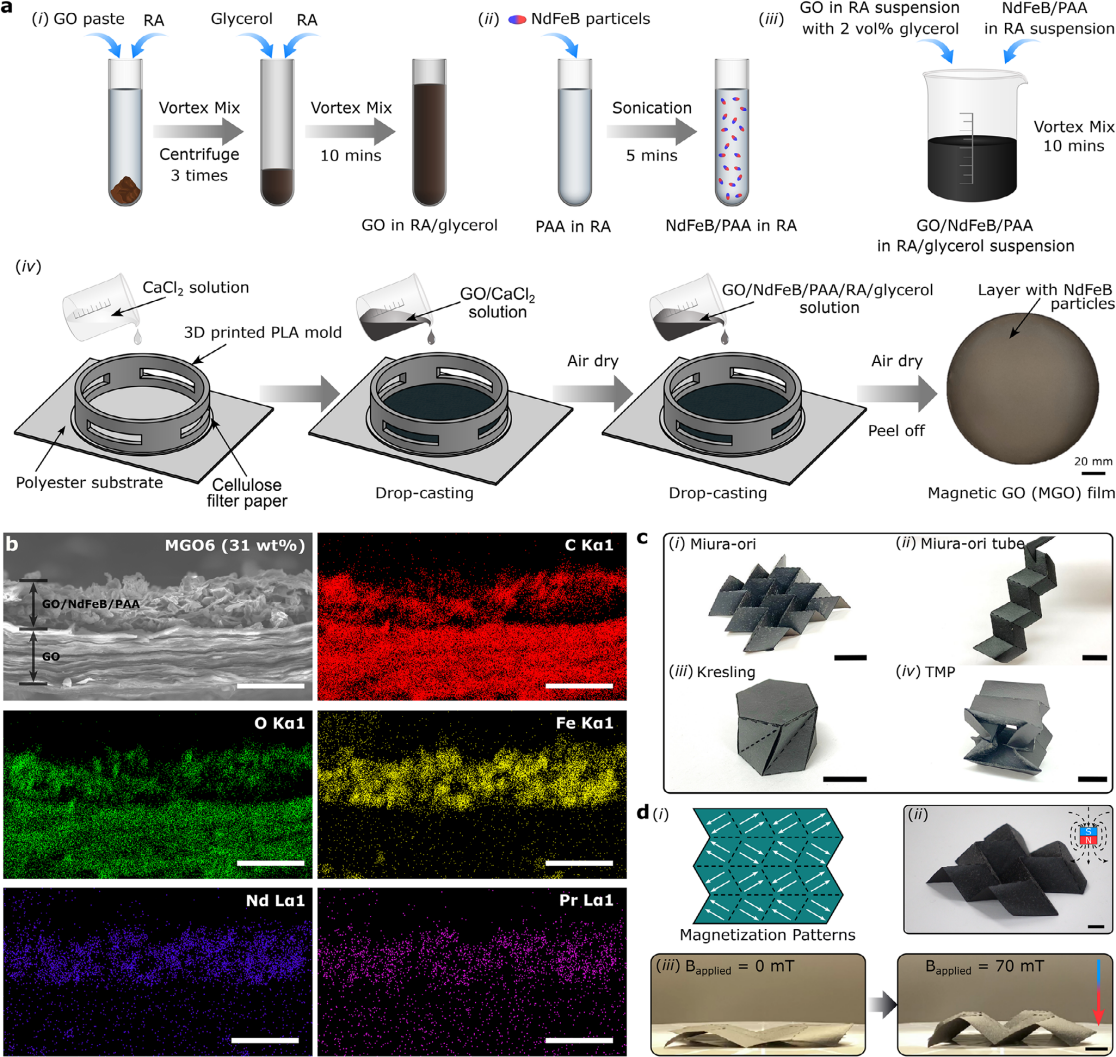
Fabrication and characterization of magnetic graphene oxide films. a) Illustration of the preparation process of MGO films. (i) Preparation of a suspension of GO in RA/glycerol; (ii) dispersion of NdFeB particles in PAA/RA solution; (iii) mixing of GO and NdFeB suspensions; (iv) preparation of MGO films. The GO/NdFeB/PAA/RA/glycerol solution is drop‐cast onto the surface of an air‐dried Ca^2+^ cross‐linked GO film. After drying the GO/NdFeB/PAA layer, a freestanding MGO film is obtained by removing the polylactic acid (PLA) mold and peeling it off from the substrate. b) SEM and EDS elemental mapping images of an MGO6 film (*ϕ* = 31 wt.%). Scale bar = 25 µm. c) Origami structures made by MGO films. Scale bar = 10 mm. d) Magnetically‐actuated shape change of Miura‐ori origami made by MGO film. (i) Schematic showing the magnetization patterns; folding of MGO Miura‐ori actuated by magnetic fields generated by (ii) a permanent magnet (beneath the sample) and (iii) a Helmholtz coil.

The MGO films consist of a layer of GO substrate and a layer of NdFeB particles embedded in GO and PAA. We prepare the bilayer MGO films by drop‐casting suspensions of GO/NdFeB/PAA/glycerol in reagent alcohol (RA) onto air‐dried CaCl_2_‐cross‐linked GO films (**Figure** **1**a and Experimental Section). The GO/NdFeB/PAA in RA suspensions contain GO (20 g L^−1^), PAA (10 g L^−1^), and NdFeB at various concentrations (Table S1, Supporting Information). RA is used as the dispersing solvent due to its quick evaporation, leaving a magnetizable layer. In the suspension, GO flakes act as physical barriers, trapping the particles and preventing their precipitation, while PAA serves as a binding agent, which solidifies after RA evaporation and ensures the binding.

First, we obtain GO in RA suspension by mixing the GO paste in RA through vortexing, followed by removing the water from the GO paste by centrifugation (Figure 1a‐i). The supernatant is removed, and RA is added to the GO paste to disperse GO in RA by vortexing. After repeating the vortexing, centrifugation and resuspension steps for 3 times, 2 vol.% of glycerol is added to the final GO/RA suspension. Glycerol acts as a plasticizer and makes the coating flexible.^[^ ^75^, ^76^ ^]^ We disperse the NdFeB particles in the PAA/RA solution by sonication (Figure 1a‐ii) and added to the GO in RA suspension through vortexing (Figure 1a‐iii). We immediately drop‐cast the mixture onto the surface of a Ca^2+^ cross‐linked GO film, which is previously prepared by drying GO/CaCl_2_ in water suspension on a cellulose paper in aid with a mold and adhesive polyester substrate (Section S2, Supporting Information). After drying of the GO/NdFeB/PAA layer, we obtain a freestanding MGO film by removing the mold and peeling off the film from the substrate (Figure 1a‐iv). The NdFeB weight fractions (*ϕ*) in the MGO films vary from 11 (MGO1) to 31 (MGO6) wt.%, as found from thermogravimetric analysis (TGA) results (Figure S1 and Table S2, Supporting Information).

The cross‐section morphology of MGO films is characterized by scanning electron microscopy (SEM) and X‐ray energy‐dispersive spectroscopy (EDS) mapping (Figure 1b; Figures S2–S6 in Supporting Information). As shown in Figure 1b, the MGO6 film features two distinguishable layers. The lower layer clearly shows stacked GO flakes containing carbon and oxygen. The upper layer, adhering to the GO substrate, includes neodymium and praseodymium throughout, in addition to carbon and oxygen, indicating the incorporation of the NdFeB particles within GO and PAA. Importantly, our fabrication process ensures that the NdFeB particles are uniformly distributed within the top layer of the MGO films (see EDS elemental mapping images in Figure 1b; Figures S2–S6 (Supporting Information), and micro‐computed tomography image in Figure S8, Supporting Information), which is crucial for achieving uniform magnetic torque under an applied field and thereby ensuring reproducible actuation behavior. The thickness of the MGO films characterized by different particle weight concentrations is determined by SEM (Figure 1b; Figures S2–S6, Supporting Information) and reported in Table S2 (Supporting Information). The MGO films remain their structural integrity in deionized water even after 30 days due to the cross‐linking of GO layer by Ca^2+^ and the presence of PAA that effectively binds the GO and the embedded particles together (Figure S7 and Movie S1, Supporting Information). However, it should be noted that while the overall film structure remains stable, the NdFeB particles themselves are prone to oxidation in humid or acidic environments, which may reduce magnetic performance during long‐term exposure.^[^ ^77^, ^78^ ^]^ In this study, magnetic actuation tests are performed immediately after fabrication, and no degradation is observed under these short‐term conditions. A detailed discussion of oxidation/corrosion effects on magnetic properties and possible protective strategies is provided in the Section S3 (Supporting Information).

Even with the addition of a magnetic composite layer onto GO film, the resulting MGO films retain the excellent folding capability of GO paper. By introducing creases with appropriately designed patterns, MGO films can be folded into a variety of origami structures (Figure 1c and Experimental Section). Different from other fabrication methods to realize magnetic soft materials (e.g., molding and casting,^[^ ^32^, ^79^ ^]^ additive manufacturing,^[^ ^16^ ^]^ microfabrication and microassembly^[^ ^40^ ^]^), our magnetic origami fabrication approach exploits pre‐existing MGO films, which allows for swift adaptation to customized structures through straightforward cutting, folding, and assembly post‐processing, thereby eliminating the need for predefined designs and significantly reducing fabrication time and complexity. For example, by laser‐cutting Miura‐ori patterns into an MGO film (Figure S10, Supporting Information) and manually folding along the designated “mountain” and “valley” creases, a 3D MGO Miura‐ori structure can be obtained within minutes, considerably faster than other reported fabrication approaches.^[^ ^16^, ^17^, ^33^, ^34^, ^35^, ^79^, ^80^ ^]^ Once magnetized in a prescribed direction (see magnetization pattern in Figure 1d‐i), MGO Miura‐ori can achieve complex and programmable motions both in air (Figure 1d; and Movie S2, Supporting Information) and water (Figure S11 and Movie S2, Supporting Information), when actuated by external magnetic fields generated by either a permanent magnet (Figure 1d‐ii) or Helmholtz coils (Figure 1d‐iii).

### 2.2 Magnetic Actuation with Humidity‐Tunable Performance

**Figure 2:**
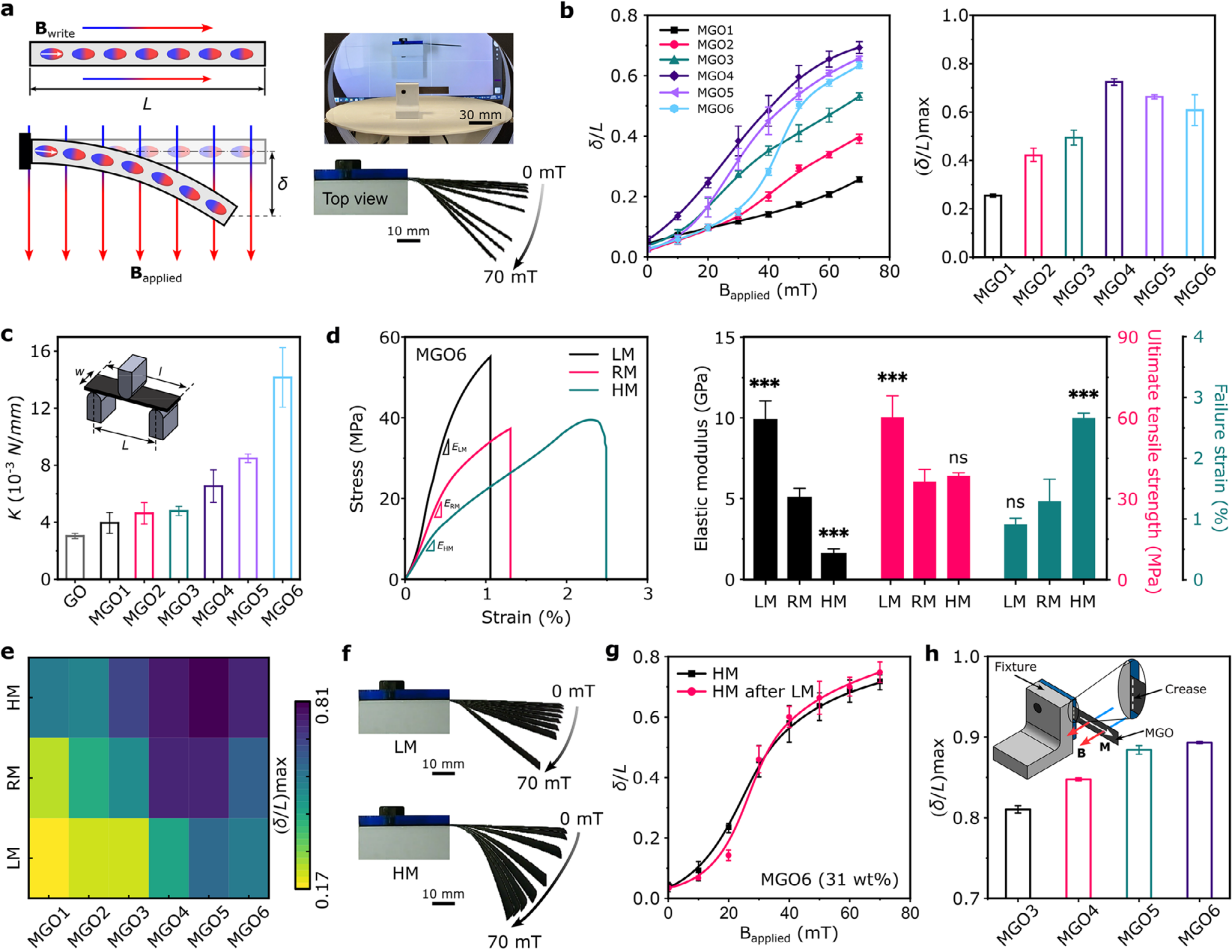
Magnetic actuation with humidity‐tunable performance of MGO films. a) MGO beam is uniformly magnetized (B_write_) along its length, and the torque‐driven cantilever bending actuation occurs under a uniform magnetic field (B_applied_) initially applied perpendicular to the MGO beam. Experimental setup for the cantilever bending actuation of MGO under a uniform magnetic field. Digital pictures of the torque‐driven cantilever bending actuation of MGO6 under various magnitudes of magnetic field (from 0 to 70 mT). b) Normalized free end deflection of MGO beams as a function of applied magnetic field at different particle weight concentrations in room moisture (RM) conditions. The maximum normalized free‐end deflection of MGO beams for different particle weight concentrations under a uniform magnetic field (B_applied_ = 70 mT) at RM. c) Bending stiffness of the MGO samples with different particle weight concentrations. The MGO sample dimensions are set to *L* = 20 mm and *W* = 20 mm. d) Stress‐strain curves of MGO6 under different moisture levels (low (LM), room (RM), and high (HM) moisture). The effect of moisture levels (i.e., LM, RM and HM, respectively) on the mechanical properties (i.e., elastic modulus, ultimate tensile strength, and failure strain) of MGO6. The data are represented as the mean ± standard deviation. The *** sign indicates statistically significant differences (*P* < 0.001) between RM and LM or HM. Differences were based on Student's t‐test. The MGO6 dogbone samples are first processed to achieve the desired moisture levels. Immediately after removal from the chamber, tensile testing is conducted at room humidity (RH ∼ 30%) and room temperature (≈23 °C). e) Effect of moisture levels (i.e., LM, RM, and HM) on normalized free‐end deflection of MGO beam with varying particle weight concentrations at B_applied_ = 70 mT. f) Digital pictures of the torque‐driven cantilever bending actuation of MGO6 under various moisture conditions (LM and HM) and magnitudes of magnetic field (from 0 to 70 mT). g) Comparison of actuation performance of the same MGO6 sample under HM following different moisture conditions. We first subject an MGO sample to magnetic‐driven deflection under HM conditions (black curve); we then heat it up to 60 °C to release water (i.e., LM), re‐expose it to a high level of humidity by locating it in a humid chamber (RH > 70%) for 2 min, and finally perform the magnetic‐driven deflection again under HM conditions (pink curve). h) Normalized free‐end deflection of creased MGO beams with different particle weight concentrations under a uniform magnetic field (B_applied_ = 70 mT) at RM.

A fully magnetized MGO film retains a large magnetic hysteresis due to NdFeB's significant remanence and coercivity.^[^ ^16^ ^]^ We first assess the magnetic responsiveness of MGO films using a simple rectangular beam uniformly magnetized along its length (**Figure** **2**a; Figure S13, Supporting Information). When a uniform magnetic field is applied perpendicularly to the longitudinally magnetized beam, the embedded hard‐magnetic particles experience a magnetic torque that tends to align their remanent magnetization with the external magnetic actuation field (Section S5C, Supporting Information). To eliminate the contribution of gravity, the surface of the MGO beam and the uniform magnetic field direction are perpendicular and parallel to the ground, respectively (Figure 2a), so that the induced magnetic torque leads to the MGO sample bending parallel to the ground.

We start by investigating the magnetically‐actuated bending of MGO beams characterized with varying particle weight concentrations in terms of the normalized free‐end‐deflection (Figure 2b) under the room moisture (RM) condition (the relative humidity, RH ≈ 55%) (Figure S19, Supporting Information). Figure 2b shows the significant bending of the MGO beam caused by the magnetic torque under room moisture (RM) conditions, notably influenced by the weight fraction of magnetic particles in its composition. MGO4 (*ϕ* = 22 wt.%) exhibits the best actuation performance, with a normalized deflection reaching over 0.7 at B_applied_ = 70 mT. The actuation performance increases by increasing particle weight concentration while *ϕ* ≤ 22 wt.%, and it declines at greater *ϕ* values. The results in Figure 2b indicate that maximizing the actuation performance of MGO actuators requires a balance between stiffness and magnetic torque. As more particles are added, magnetization (**M**) and thus magnetic torque increase, but stiffness also rises (Figure 2c; Figure S14, Supporting Information). Consequently, the actuation performance of the MGO beam under a uniform magnetic field is dictated by the trade‐off between increasing magnetic torque and bending stiffness as a function of magnetic particle loading (Section S5C, Supporting Information). In addition, our MGO actuators demonstrate excellent durability. No significant degradation in actuation amplitude is observed after 50 loading cycles (Figure S16, Supporting Information), and no cracks or defects appear around the creases following repeated bending deformation (Figure S17, Supporting Information), confirming the mechanical robustness and reliability of the MGO films. We also note that actuation performance in magnetoactive systems is frequency‐dependent.^[^ ^20^, ^81^, ^82^ ^]^ While higher frequencies increase actuation speed, they also reduce degree of actuation (see Figure S18, Supporting Information), consistent with observations in magnetoactive soft robots.^[^ ^72^, ^73^, ^74^ ^]^ Compared with conventional actuators,^[^ ^16^, ^83^, ^84^, ^85^, ^86^, ^87^, ^88^, ^89^, ^90^, ^91^, ^92^, ^93^, ^94^ ^]^ our MGO actuators exhibit significantly higher actuation rates while operating at lower energy densities (Figure S23a, Supporting Information). In addition, when compared to conventional GO‐based actuators driven by humidity or light stimuli,^[^ ^1^, ^2^, ^64^, ^95^, ^96^, ^97^ ^]^ the MGO actuators demonstrate controllable and faster actuation speeds (Figure S23b, Supporting Information).

In addition to the magnetic loading, environmental humidity introduces an additional degree of freedom for tuning the mechanical and actuation behavior of MGO films. GO films are hygroscopic, absorbing water molecules from the environment and releasing them when heated.^[^ ^96^ ^]^ Upon water absorption, the GO layers in MGO films swell and their interlayer spacing increases, thereby becoming softer and more flexible^[^ ^61^, ^62^, ^98^ ^]^ (Figure 2d). To investigate the coupling between moisture and magnetic response, MGO beams are conditioned under two additional moisture levels: low moisture (LM) and high moisture (HM) (Figure S19, Supporting Information). These conditioning processes allow the MGO beams to release or absorb water from the environment before conducting the magnetically driven deflection test (refer to Experimental Section and Section S5E, Supporting Information). In Figure 2e, we report the normalized deflection (at B_applied_ = 70 mT) of MGO beams conditioned under different moisture with different particle weight fractions. The actuation performance (i.e., deflection of the tip of the MGO beam) is enhanced by water absorption (i.e., HM condition) and inhibited by water release (i.e., LM condition) (Figure 2f; Figure S20, Supporting Information). Importantly, the magnetically‐driven response of MGO beams remains stable and reversible after multiple LM–HM cycling tests (Figure 2g), demonstrating the robustness of humidity‐controlled tuning. This dual‐field modulation (i.e., magnetism for actuation and humidity for stiffness control), provides unprecedented tunability for programmable, multimodal deformation. While magnetic fields govern the direction and magnitude, humidity offers fine control over deformation range, speed, and energy efficiency. Such synergistic coupling between humidity and magnetism is rarely reported in magnetoactive systems and represents a promising route toward enhanced functional control.

We next use the MGO origami structures as actuators (see the fabrication of MGO origami structures in Experimental Section). To understand how they deform under a magnetic field, we first perform preliminary experiments with creased MGO beams, mimicking the presence of creases in MGO origamis. In origami, creases are significantly softer than panels, and the overall deformation primarily occurs through the folding and unfolding of these creases.^[^ ^13^, ^99^ ^]^ Therefore, we investigate the magnetic actuation of creased MGO beams, with the crease introduced near the fixed end of the beam (Figure 2h). We focus specifically on MGO3 to MGO6, as they exhibit notably higher deflection under magnetic actuation compared to MGO1 and MGO2 (Figure 2b). The deflection of creased MGO beams increases with the enhancement of particle concentration, with the maximum deflection observed in MGO6 (Figure 2h; and Section S5E, Supporting Information). In addition, the significant reduction in stiffness at the crease minimizes the impact of moisture on the deflection of creased MGO6 samples compared to uncreased MGO samples (Figure S21, Supporting Information). Based on these results, we select MGO6 (*ϕ* = 31 wt.%) as the film used to prepare origami structures in the rest of this work.

### 2.3 Programmed Shape Morphing of MGO Origami

**Figure 3:**
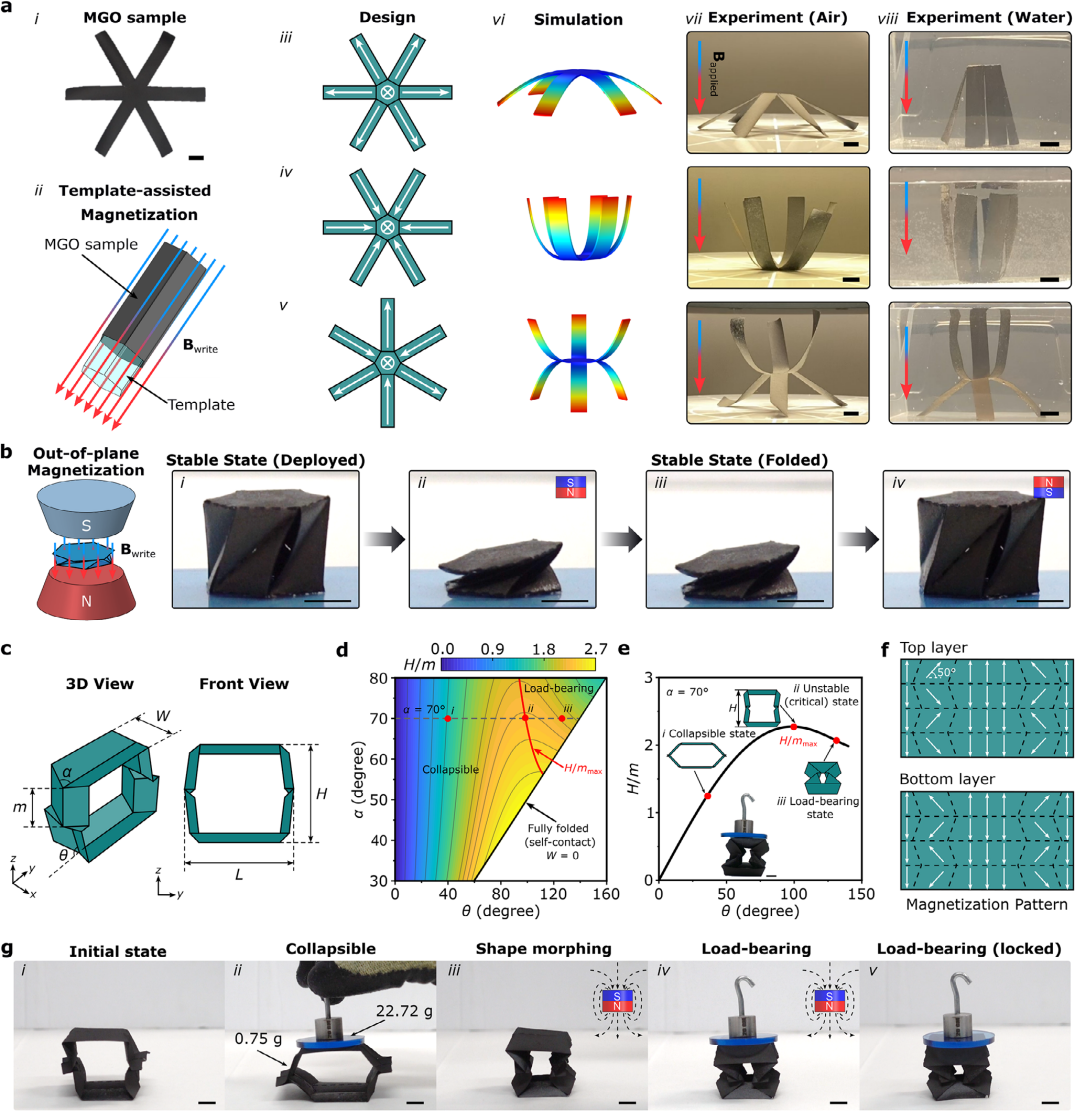
Magnetically‐actuated shape morphing of MGO origami structures. a) MGO samples, schematic designs, finite element simulation and experimental results (conducted both in air and water) for six‐armed MGOs. (i) Digital picture of a six‐armed MGO sample. (ii) A template‐assisted magnetization method is used to magnetize the designed MGO origami structures. The light blue represents a hexagonal prism template where the six arms of MGO are folded downward for magnetization. In the case of the “chair” MGO, a second identical template is used, where three arms are folded upward and three arms are folded downward for magnetization. (iii)‐(v) Schematics of magnetization patterns for six‐armed MGOs, which can deform into arm‐down, arm‐up, and chair configurations under a uniform magnetic field, respectively. (vi) Finite element simulation results. Experimental observation of magnetically‐actuated shape changes (vii) in air and (viii) in water. b) A bistable MGO Kresling is magnetized in its fully folded state. MGO Kresling unit cell switches from a stable deployed state to a stable folded state upon application of a magnetic field by a permanent magnet, and transitions back to the stable deployed state upon the application of a field in the opposite direction (i.e., after rotating the permanent magnet by 180°). Notably, the bistable MGO Kresling stays in the deployed or folded states after removing the magnetic field. The bottom panel of MGO Kresling origami is affixed to the ground using double sided tape to ensure transformations between bistable states c) A Tachi‐Miura Polyhedron (TMP) origami unit cell in the 3D view and front view. d) Contour plot of the height of the TMP unit cell normalized by the crease length (*H*/*m*) as a function of angle *α* and folding angle *θ*. The red curve represents the maximum height of TMP with given geometrical parameters during the folding/deploying process. The black line represents the critical folding angle where the TMP unit cell is fully folded in the *x*‐direction (i.e., *W* = 0). (i)‐(iii) represent the collapsible, unstable (critical), and load‐bearing states, respectively (see Figure 3e). e) The normalized height (*H*/*m*) of TMP characterized by *d* = *m* = *l*, and *α* = 70° as a function of folding angle *θ*. The inset shows the cross‐sectional geometry (front view) at three folding angles, corresponding to different configurational states: (i) collapsible, (ii) unstable (critical), and (iii) load‐bearing states. f) Magnetization patterns of TMP. MGO TMP is magnetized when it is fully folded in the x‐direction. g) Shape morphing of MGO‐TMP from a collapsible mode to a load‐bearing mode actuated by a permanent magnet. In its initial state, MGO‐TMP is collapsible and lacks load‐bearing capability ((i) and (ii)). A permanent magnet placed underneath the MGO‐TMP induces shape morphing (iii) to a load‐bearing configuration (iv), which can hold a weight even after removal of the permanent magnet (v).

To illustrate the magnetic actuation performance of MGO, we investigate the magnetically controllable shape morphing of the origami structures fabricated by MGO films. We first examine simple six‐armed MGOs, fabricated through laser cutting (**Figure** **3**a‐i). Using a template‐assisted magnetization method,^[^ ^40^ ^]^ the six‐armed MGO is first deformed into a desired temporary shape, then magnetized to saturation by a strong magnetic field (Figure 3a‐ii)^[^ ^40^ ^]^ (see magnetization in Experimental Section). Different magnetization patterns are implemented in the six‐armed MGOs (Figure 3a‐iii–v), resulting in distinctive configurations such as “arm‐up”, “arm‐down”, and “chair” under a uniform applied magnetic field (Figure 3a; and Movie S3, Supporting Information). Finite element analysis (FEA) is employed to facilitate the design of origami magnetization patterns, with simulation results matching experimentation, indicating FEA's effectiveness in predicting magnetically actuated shape morphing (see Figure 3a‐vi and Experimental Section). The MGO's stability in water and the increased softness after water absorption allow for even more dramatic and faster shape changes in water under the same magnetic field (Figure 3a‐viii; and Movie S3, Supporting Information).

Different from other fabrication methods for magnetic soft materials,^[^ ^16^, ^19^, ^33^, ^34^, ^36^, ^40^ ^]^ our approach, based on cutting, folding, and assembly, is remarkably simple and energy‐efficient. To demonstrate this, we create a Kresling origami out of the MGO film (Figure 3b; Figure S24, Supporting Information) and magnetize it in its fully folded state (Figure 3b). Originating from the buckling of thin shell cylinders,^[^ ^100^ ^]^ the Kresling is a non‐rigid foldable origami, allowing for unit cell bistability.^[^ ^32^, ^101^ ^]^ The bistable Kresling origami with the deployed stable state (Figure 3b‐i) and the folded stable state (Figure 3b‐iii) is achieved by geometrical design (Section S6, Supporting Information). The MGO Kresling origami can switch between its folded and deployed stable states upon application of magnetic fields in opposite directions using a permanent magnet (Figure 3b‐i–iv; and Movie S4, Supporting Information).

So far, we have studied the magnetically‐actuated behavior of origami structures, the ability to create complex deployed structures and program magnetization patterns allows achieving different functionalities. We showcase the remote‐actuated, in situ transition between different functionality modes in origami structures. Specifically, we fabricate a Tachi‐Miura Polyhedron (TMP) origami, a bellows‐like 3D origami based on Miura‐ori cells comprising two sheets,^[^ ^102^ ^]^ using MGO films (MGO‐TMP), which exhibit distinctive mechanical properties across different folded states (Figure 3c; Figure S25, Supporting Information). However, it is important to note that not all TMP unit cells exhibit bifurcated folding motions, this behavior depends on the geometrical parameters of the patterns (see Section S7B, Supporting Information). Through kinematic analysis, we derive closed‐form expressions for the critical folding angle *θ* (indicated by the red curved line in Figure 3d), which dictates the mechanical bifurcation between collapsible and load‐bearing configurations. Detailed theoretical studies on the mechanical behaviors of TMP, influenced by various geometrical parameters (e.g., *d*/*m* and *α*) and folding angles (*θ*), are extensively documented in Section S7B (Supporting Information). With specified geometrical parameters (e.g., *d* = *m* = *l*, and *α* = 70°), TMP origami can transition between collapsible and load‐bearing states by changing its configuration, which is determined by the folding angle *θ* (Figure 3e). Thanks to the programmed magnetization patterns (Figure 3f), the load‐bearing and collapsible configurations of an MGO‐TMP origami can be interchanged by a uniform magnetic field applied within a Helmholtz coil (Figure S27, Supporting Information) or through a non‐uniform magnetic field applied by a permanent magnet (Figure 3g; and Movie S5, Supporting Information). In its initial state, the MGO‐TMP origami is collapsible and cannot bear any load (i and ii in Figure 3g). However, a permanent magnet can remotely actuate the shape morphing of the MGO‐TMP from a collapsible state to a load‐bearing one (Figure 3g‐iii), enabling the MGO‐TMP origami to support a load 30 times higher than its own weight (Figure 3g‐iv). The MGO‐TMP can still support the load after removing the permanent magnet (Figure 3g‐v).

### 2.4 MGO Origami Soft Robots with Multimodal Motions

**Figure 4:**
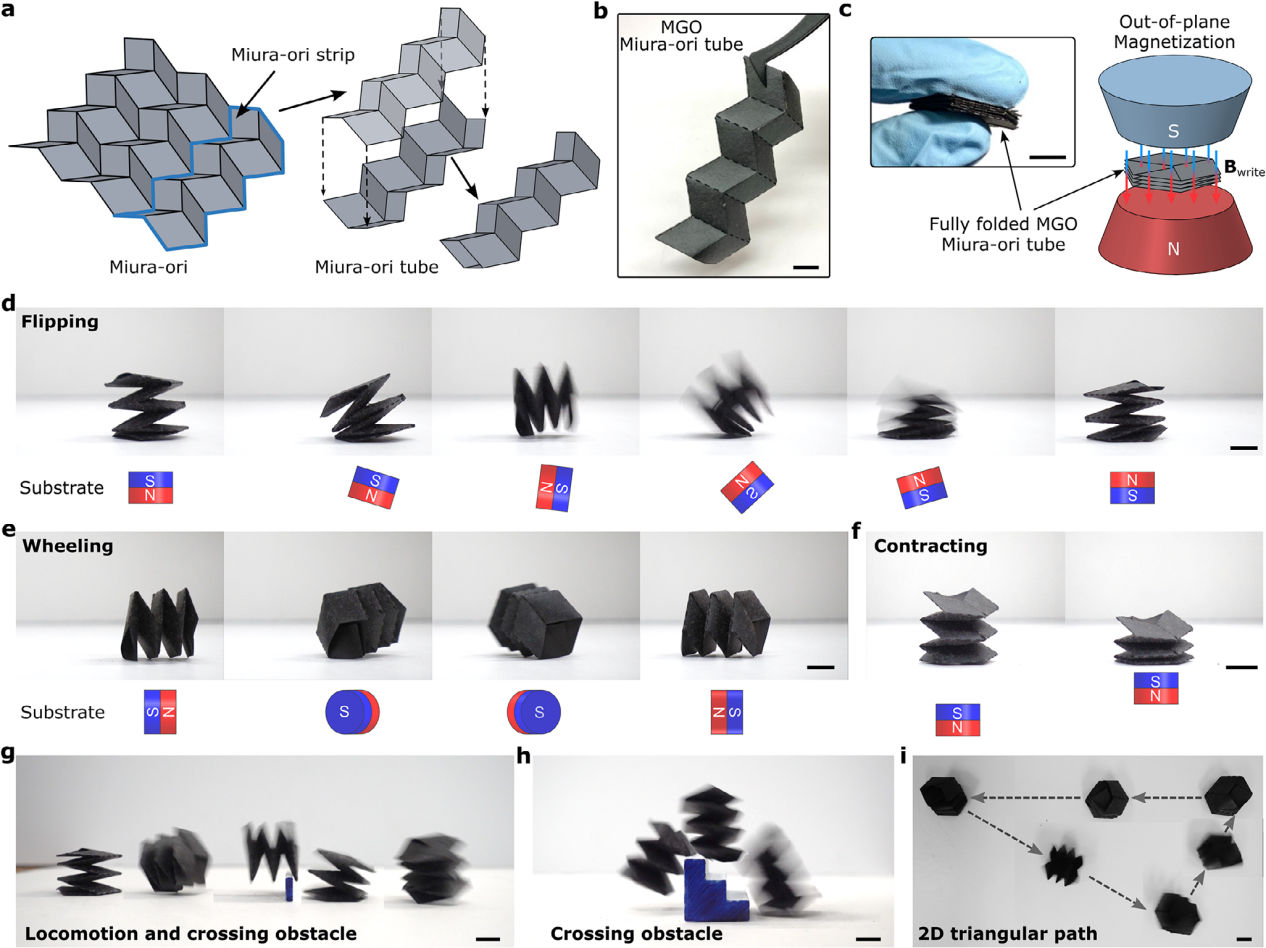
Locomotion of MGO Miura‐ori tube structure actuated by a permanent magnet. a) Fabrication of Miura‐ori tube, which is assembled from two mirrored Miura‐ori strips. b) Digital picture of Miura‐ori tube fabricated by MGO films through simple cutting, folding, and sticking process. c) The MGO Miura‐ori tube is magnetized at its fully folded state. Two locomotion modes: d) flipping and e) wheeling, achieved by rotating a permanent magnet beneath the MGO soft robot. f) Contraction achieved by vertically moving a permanent magnet beneath the MGO soft robot. Under a spatially varying magnetic field generated by a permanent magnet, the MGO soft robot navigates and overcomes various obstacles, including g) a wall and h) stairs. i) Demonstration of locomotion of the MGO soft robot along a 2D triangular path under a spatially varying magnetic field. Scale bar = 10 mm.

In addition to enabling shape morphing and transition between different functional modes in origami structures, MGO can be used to develop soft robots with multimodal locomotion capabilities. The fist demonstration of the soft robot is designed based on the Miura‐ori tube, which is constructed by assembling two mirrored Miura‐ori strips (**Figure** **4**a). Figure 4b shows a Miura‐ori tube fabricated out of MGO films, which is then magnetized at its fully folded state (Figure 4c). Subsequently, the Miura‐ori tube can be fully compressed under applied magnetic fields generated by a permanent magnet and return to an extended state upon application of magnetic fields in opposite direction (Figure S12 and Movie S2, Supporting Information). When moving and rotating the permanent magnet continuously beneath the MGO soft robot, the robot's magnetization tracks the magnetic field, resulting in various multimodal motions (see Movie S6, Supporting Information) including straight walking (Figure S28, Supporting Information), flipping (Figure 4d; Figure S29, Supporting Information), wheeling (Figure 4e), and contracting (Figure 4f). By combining vertical, horizontal, and rotational movements of a permanent magnet to create spatially varying magnetic fields for dynamic actuation, the MGO soft robot can navigate and overcome various obstacles on an unstructured ground, including a wall (10 mm height) and stairs (of similar size to the robot, 25 mm) (Figure 4g,h; Figures S30 and S31 and Movie S6, Supporting Information). Leveraging the feasible and convenient navigation enabled by the permanent magnet‐assisted actuation, Figure 4i showcases agile navigation along a triangular path through a multimodal locomotion, including flipping and wheeling (Movie S6, Supporting Information). It should be noted that the navigation path depends on the movement of the permanent magnet, enabling the MGO soft robot to navigate along complex trajectories.

**Figure 5:**
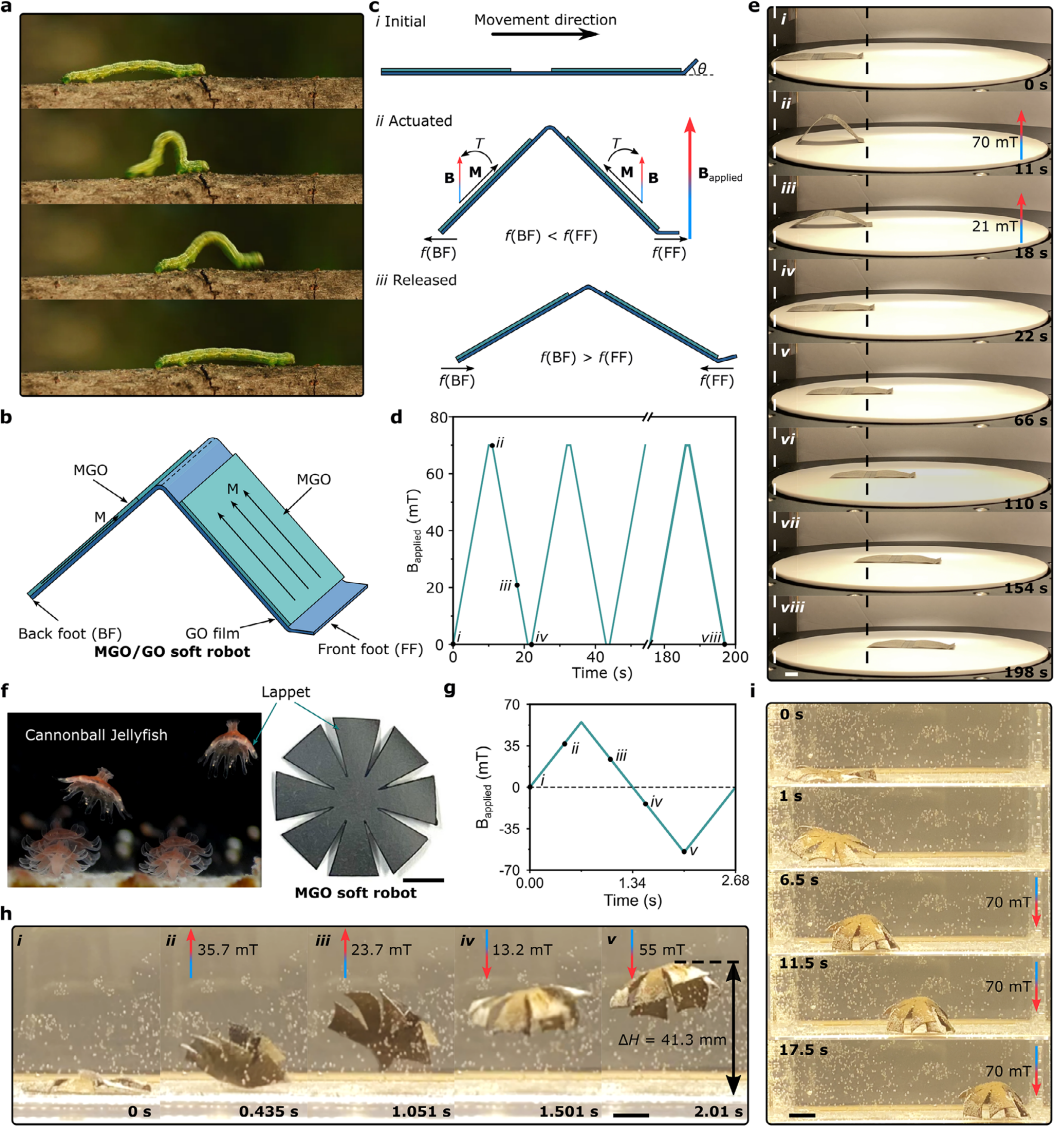
Magnetically induced motion of bio‐inspired MGO soft robots. a) The locomotion of an inchworm. Snapshots from the video “An Inch worm making its way across a tree branch” (Stock Video ID: 6 246 545), reproduced with Shutterstock Standard License. b) Design of an inchworm‐inspired MGO/GO soft robot. c) Schematics illustrating the locomotion mechanism of the inchworm‐inspired soft robot. d) Applied magnetic field as a function of time used to actuate the locomotion of the inchworm‐inspired soft robot. The specific motions of the MGO/GO soft robot under corresponding magnetic fields (i)‐(viii) are shown in (e). e) Magnetically‐driven locomotion of the MGO/GO soft robot, demonstrating a movement of ≈85 mm at a walking velocity of ≈0.43 mm s^−1^. f) Swimming of the ephyra of the cannonball jellyfish. Copyright 2020, Springer Nature; Graphs of cannonball jellyfish reproduced with permission.^[^ ^104^ ^]^ Design of the jellyfish‐inspired MGO swimming soft robot with eight lappets. g) Magnetic field applied as a function of time to actuate the swimming of the jellyfish‐inspired soft robot. The specific motions of the MGO soft robot under corresponding magnetic fields (i)‐(v) are shown in (h). h) Magnetically‐driven upward swimming of the fabricated MGO soft robot, which ascends to a height of 41.3 mm. i) A soft robot with asymmetric magnetization patterns swims from the left to the right side under a magnetic field. Scale bar = 10 mm.

Whereas Figure 4 highlights the multimodal locomotion capabilities enabled by MGO origami actuated by a permanent magnet, we then proceed to realize MGO‐based bio‐inspired soft robots actuated by a uniform magnetic field generated by a single‐axis Helmholtz coil. We first mimic the motion of an inchworm, a caterpillar of the geometer moth, which has two pairs of prolegs at its rear end and three pairs of true legs at the front (**Figure** **5**a).^[^ ^103^ ^]^ To mimic this locomotion, we design a soft robot with a body composed of GO and MGO (GO and MGO are colored in blue and turquoise, respectively, in Figure 5b) where MGO is characterized by in‐plane magnetization acting as the longitudinal muscle fibers of the inchworm to actuate body bending (dimensions can be found in Figure S32, Supporting Information). Upon application of a magnetic field perpendicular to the ground, the magnetic torque induces a bend in the robot's body like the inchworm's abdominal contractions (Figure 5c‐ii). The difference in the feet design results in asymmetric friction during the actuated and released states (see Section S8, Supporting Information). In the actuated state, the front foot develops higher friction and anchors the body, enabling the back foot to move forward. During the release state, the lower friction at the front foot allows it to slide forward, propelling the robot in the forward direction (Figure 5c‐iii). Under the applied magnetic field (Figure 5d), the inchworm‐inspired MGO/GO soft robot moves ≈85 mm after nine cycles of magnetic actuation at a walking velocity of ≈0.43 mm s^−1^ (Figure 5e; and Movie S7, Supporting Information). Although we utilize a relatively low magnetic field frequency (≈0.046 Hz), the robot maintains a competitive walking velocity, which can be increased by adjusting the magnetic field frequency.^[^ ^20^ ^]^

Following the demonstration of a soft robot designed for terrestrial applications, we introduce another application of the MGO films: a jellyfish‐like magnetically‐driven soft robot tailored for aquatic environments, which can produce controlled fluid flows around its body using its lappets actuated by a remote magnetic field (Figure 5f). This jellyfish‐like soft robot (dimensions can be found in Figure S34, Supporting Information) is capable of moving its eight lappets up and down in a non‐reciprocal manner like an ephyra, controlled by an external magnetic field. For upward swimming, the jellyfish‐like soft robot is magnetized in a contracted state with all lappets bent downward, creating rotationally symmetrical magnetization patterns (Figure S35a, Supporting Information). The propulsion phase of the jellyfish‐inspired MGO soft robot is characterized by two states of contraction and recovery,^[^ ^80^ ^]^ controlled by regulating the direction of the magnetic field (Figure 5g; and Movie S7, Supporting Information). Initially, the MGO soft robot is located at the bottom of the container (Figure 5h‐i, Supporting Information). It rapidly contracts and surges upward when an upward‐directed magnetic field is applied (Figure 5h‐ii). The quick swings of lappets generate a pressure differential, drawing water into the space beneath the robot and propelling it upwards. As the magnetic field direction reverses from upward to downward, the soft robot flaps its lappets downward to create an upward drift flow, further lifting the robot to a higher position (Figure 5h‐iv,v, Supporting Information). By mimicking the motion of a jellyfish, the MGO soft robot can ascend to a height of 41.3 mm through magnetic actuation. While rotationally symmetric magnetization patterns enable the soft robot to move upward in a straight path, asymmetric magnetization (Figure S35b, Supporting Information) causes different bending deformations of lappets under the magnetic field (two right lappets show more pronounced bending with their ends touching the bottom of the container), allowing the soft robot to swim from the left side to the right end of the container with a crawling velocity of ≈3.89 mm s^−1^ (Figure 5i; and Movie S7, Supporting Information).

### 2.5 Reprogrammability of Magnetization

**Figure 6:**
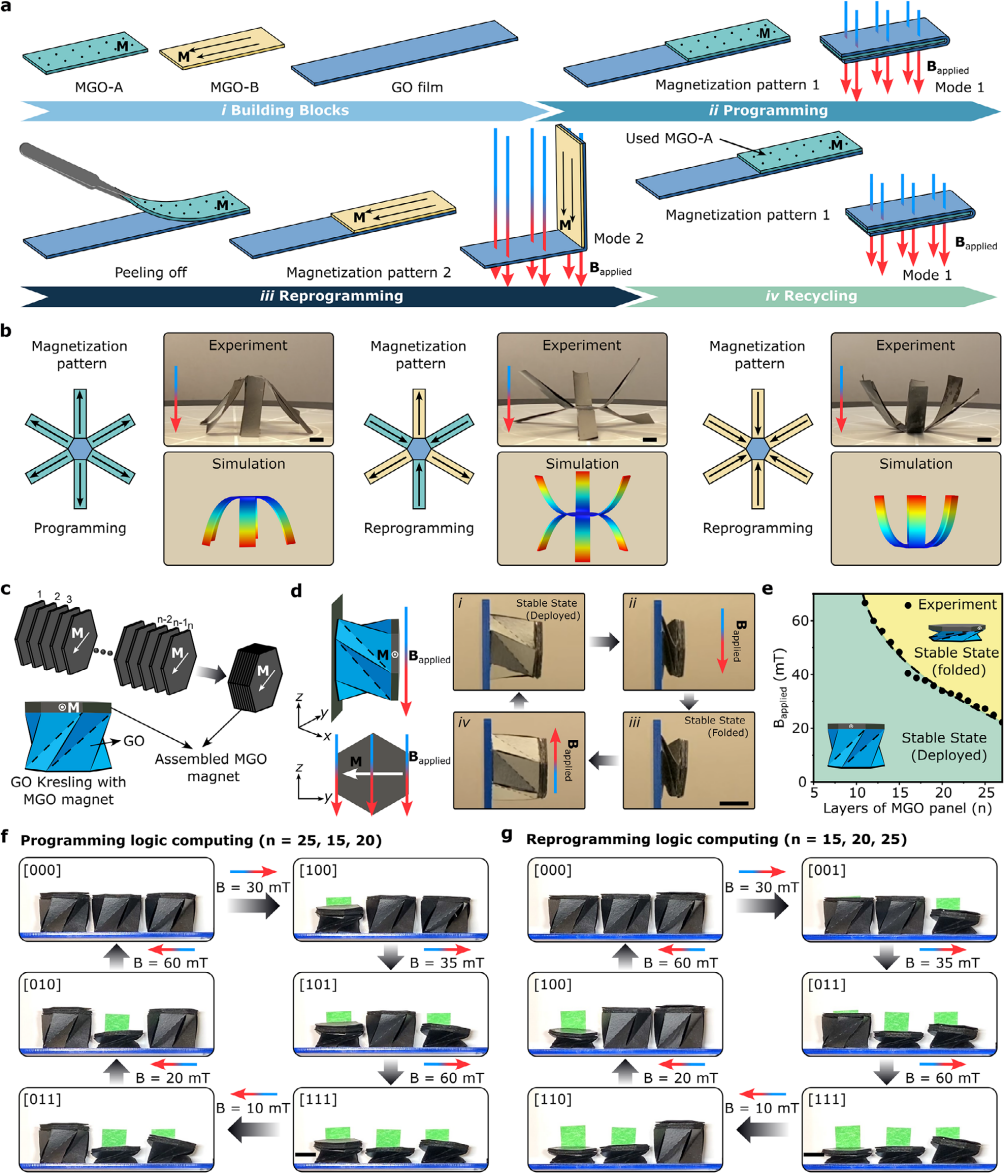
Programming and reprogramming magnetization in MGO. a) Schematic showing the steps for programming and reprogramming the magnetization patterns in a single GO film. (i) Two MGO stickers with different magnetization patterns. The magnetization patterns in MGO‐A and MGO‐B are characterized by out‐of‐plane magnetization (indicated by points) and in‐plane magnetization (indicated by arrows), respectively. (ii) The MGO‐A sticker is attached to the GO film to program its deformation of GO film under a magnetic field. (iii) The MGO‐A sticker is replaced with MGO‐B sticker to reprogram the deformation of GO film under the same magnetic field. (iv) The detached MGO‐A sticker is reusable. b) Illustration of programming and reprogramming magnetization patterns in a six‐armed GO structure, resulting in various configurations such as “arm‐up”, “arm‐down”, and “chair” under an applied magnetic field. Instead of fabricating three six‐armed MGO structures with different magnetization patterns, MGO stickers are used to program and reprogram the magnetization patterns in a single six‐armed GO structure. The turquoise and yellow represent the MGO stickers, and the blue represents the GO substrate. c) An assembled MGO magnet composed of multilayered hexagonal MGO panels is glued on top of a GO Kresling origami. The assembled MGO magnet is created by sticking successive layers of MGO panels using PAA glue. d) The resulting GO/MGO Kresling origami has an in‐plane magnetization M and is subjected to an external magnetic field B. When the magnetic torque is high enough to overcome the snapping torque threshold, the GO/MGO Kresling origami folds or unfolds switching between two stable states depending on the direction of the applied magnetic field generated by a Helmholtz coil. Scale bar = 10 mm. e) Contour plot showing the correlation between the number of attached MGO layers and the required magnitude of magnetic field necessary to overcome the magnetic torque threshold and result in transition of the GO/MGO origami from the deployed stable state [0] to the folded state [1]. Demonstration of f) programming and g) reprogramming logic computing in GO/MGO Kresling origamis with varying layers of MGO panels to induce stable state transitions. Initially, the three Kresling origamis are built with n_1_ = 25, n_2_ = 15, and n_3_ = 20 and a sequence of [000], [100], [101] [111], [011], [010], [000] is obtained upon application of magnetic fields of 30, 35, 60, −10, −20, and −60 mT (f). Then, ten layers of MGO panels are removed from the left Kresling unit cell and redistributed equally between the center and right Kresling units, resulting in n_1_ = 15, n_2_ = 20, and n_3_ = 25. The new sequence of state achieved with the same application of magnetic field is [000], [001], [011], [111], [110], [100] and [000] (g).

The on‐demand manufacturing capability of MGO films demonstrates its capacity to create diverse structures and magnetization patterns (Figure 3), facilitating the realization of various functionalities (Figures 4 and 5). However, MGO films produced with the methods described thus far have fixed magnetization patterns, limiting their configuration and functionality. Here we introduce a novel, convenient, and energy‐efficient strategy for programming and reprogramming magnetization patterns to fulfill the aforementioned requirements (**Figure** **6**a). Initially, two distinct sets of MGO rectangular strips (referred to as MGO‐A and MGO‐B) created by laser cutting are magnetized in different directions, one out of plane (MGO‐A) and the other in plane (MGO‐B). These MGO strips function as magnetic “stickers” affixed to different regions of a GO substrate, enabling the programming of magnetization patterns essential for generating a soft magnetic actuator. To reprogram the magnetization patterns, the removal of MGO‐A strips followed by the attachment of MGO‐B strips onto the GO substrate is all that is required to achieve a new configuration under a magnetic field. This reprogramming process, facilitated by the straightforward replacement of one MGO pattern with another with different magnetization characteristics, underscores the versatility of this approach, applicable not only to 2D patterns but also to 3D structures. The detached MGO stickers can be reused, contributing to the sustainable utilization and recycling of magnetic materials.

An example of a six‐armed magnetic structure showing different shape changes under the same magnetic field by reprogramming the magnetization illustrates the magnetic‐sticker‐assisted magnetization reprogramming (Figure 6b; and Movie S8, Supporting Information). Unlike the initial fabrication method shown in Figure 3a, where the three six‐armed structures were constructed using MGO films, we used a GO film for the body of the six‐armed structure and magnetized MGO stickers for the arms (Movie S8, Supporting Information). A specialized adhesive, PAA, ensures strong yet detachable bonding between the MGO layer and the GO substrates (Section S9, Supporting Information). To reprogram the magnetization pattern, the MGO magnetic stickers can be easily removed and replaced with new magnetic stickers featuring different magnetization patterns, allowing efficient controllable reconfiguration under a magnetic field. The detached MGO stickers can be reused, contributing to the sustainable and responsible use of magnetic materials (Movie S8, Supporting Information). In addition, MGO stickers can adhere to various substrates beyond GO films, such as cellulose paper, broadening the soft magnetic actuator fabrication capabilities. The MGO strips are powerful enough to move even heavier origami structures made from cellulose paper rather than GO paper (Figure S40, Supporting Information).

In addition to the shape reconfiguration, magnetic‐sticker‐assisted magnetization reprogramming strategy also enables tuning the amount of magnetic field required to achieve a specific deformation in magnetoactive structures. As illustrated in Figure 6c, we create a bistable Kresling origami using a GO film and add an assembled MGO magnet composed of multilayered hexagonal MGO panels on the top of the GO Kresling unit cell. The assembled MGO magnet is created by sticking successive layers of MGO panels using PAA glue. This multilayered MGO magnet generates a magnetic torque in the presence of an external magnetic field; the underlying GO Kresling origami transitions to a deployed or folded stable state depending on the direction of the applied magnetic field, when the induced magnetic torque is large enough to overcome the torque threshold for the state transformation (Figure 6d; Figure S41 and Movie S9, Supporting Information). This magnetic torque is determined by several factors: the magnitude of the applied magnetic field, the number of layers in the assembled MGO magnets (Figure 6c), and the angle between the directions of magnetic field and the magnetization (a constant 90° angle in our study). While the torque threshold is constant, the required magnetic field to overcome the torque threshold can be tuned by adjusting the number of layers in the assembled MGO magnet (Figure 6e). The ease associated with detaching MGO layers in the hexagonal panel can facilitate controlling the critical magnetic field magnitude for stable state transition.

Finally, we demonstrate the reprogramming capability of MGO films through the application of MGO Kresling origami in digital computing. We treat the applied magnetic field magnitude as the input signal, and the resultant mechanical states of the MGO Kresling as digital output [0] (deployed state) or [1] (folded state), effectively creating a mechanologic system. In Figure 6f, we design a device for three‐bit information storage using three MGO Kresling structures with identical geometrical parameters but different magnetization (**M**) due to varying number of MGO panel layers (n_1_ = 25, n_2_ = 15, and n_3_ = 20), starting from the mechanologic state [000] (three deployed states). We apply a uniform magnetic field that increases linearly from 0 to 60 mT, and then reduces to −60 mT. With the applied magnetic field, the global mechanologic states transition in the following order: [000] – [100] – [101] – [111] – [011] – [010] – [000] (Figure 6f; and Movie S10, Supporting Information). By programming the applied magnetic field, different mechanologic outputs can be achieved (Figure S42, Supporting Information).

The sequence of the global mechanologic states in a loop is determined by the number of MGO layers, offering a method to program logic computing; since the MGO layers can be easily detached and reused, reprogramming can be achieved by simply detaching and re‐attaching MGO layers in different configurations. As an example, we remove ten layers of MGO layers from the first Kresling and redistribute them equally between the second and third Kresling origami (n_1_ = 15, n_2_ = 20, and n_3_ = 25) to create a new logic computing device (Figure 6g). Using the same input conditions as adopted in Figure 6f, the newly reprogrammed configuration yields a different sequence of global mechanologic outputs (i.e., [000] – [001] – [011] – [111] – [110] – [100] – [000]) (Figure 6g). While this demonstration highlights the basic potential of MGO Kresling for logic computing, more complex logic circuits capable of performing Boolean operations like AND, OR, and NOT can be developed for applications in sensing, memory, and computation.^[^ ^32^, ^105^ ^]^

### 2.6 Proof‐of‐Concept Sensoriactuator Based on MGO

**Figure 7:**
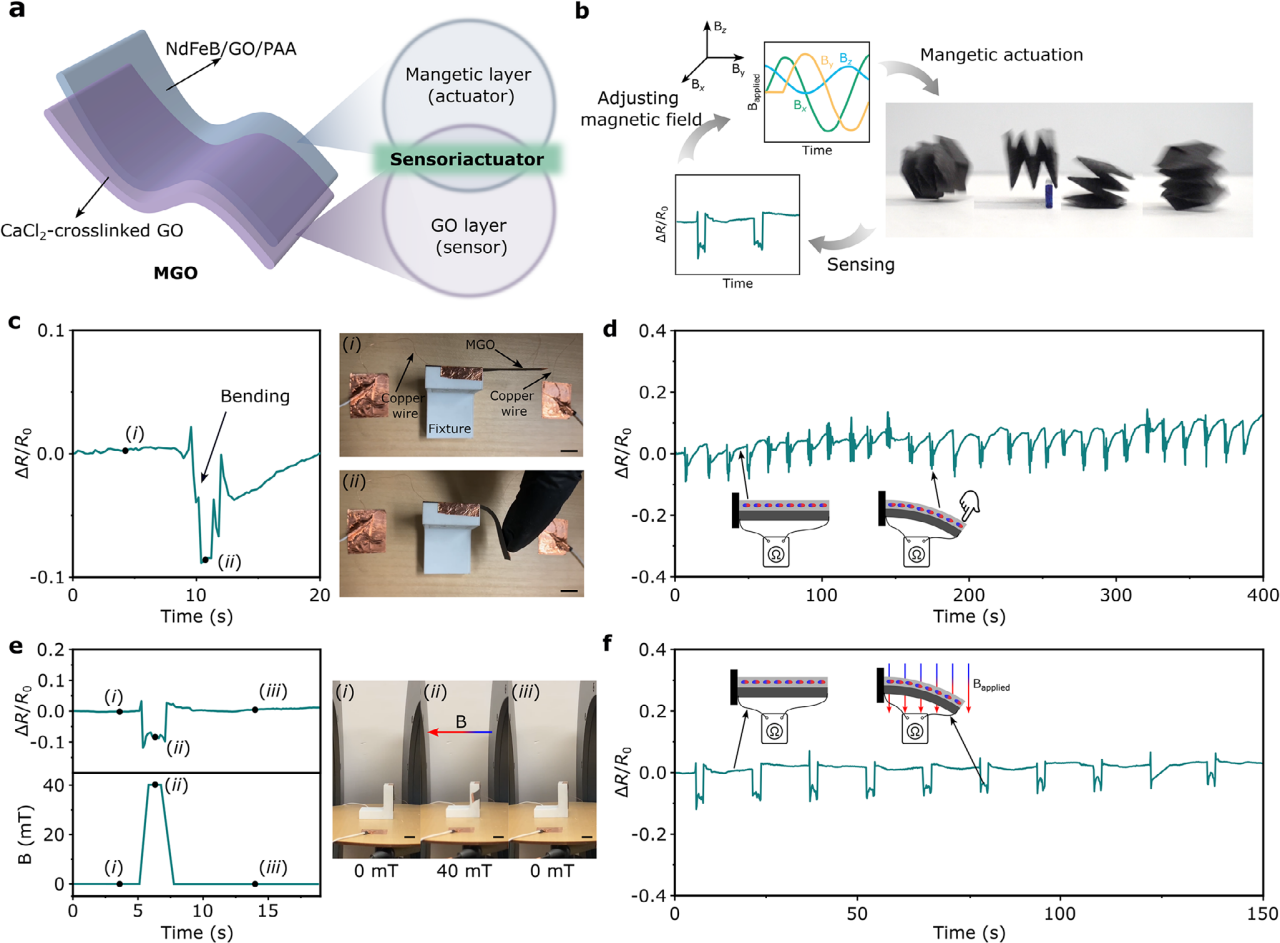
Integration of actuation and sensing in MGO films for a self‐sensing actuator. a) Schematic of the bilayer MGO films: the magnetic composite layer enables actuation under an external magnetic field, while the GO layer functions as a strain sensor through electrical resistance changes. b) Conceptual feedback control loop illustrating the integrated actuation‐deformation‐sensing‐magnetic adjustment cycle for closed‐loop operation. c) Resistance response of an MGO beam under manual bending, showing clear correlation between the bending deformation and relative resistance change (Δ*R*/*R*_0_, *R*_0_ is the electrical resistance of MGO film without deformation); scale bar = 10 mm. d) Cyclic manual bending test demonstrating stable and repeatable sensing behavior across multiple actuation cycles. e) Real‐time monitoring of relative resistance changes in the MGO beam under magnetically induced deformation using a Helmholtz coil; scale bar = 10 mm. f) Repeated magnetic actuation cycles showing consistent and reversible resistance responses, confirming the integrated actuation‐sensing functionality of the MGO film.

To demonstrate the integrated sensing‐actuation (a capability found in sensoriactuators) of our MGO films, we conduct proof‐of‐concept experiments showing simultaneous deformation and resistance feedback within the same platform (**Figure** **7**). The MGO film is composed of two functional layers (Figure 7a): a magnetically active composite layer that responds to applied magnetic fields (i.e., actuator), and a GO layer whose electrical resistance varies with mechanical deformation^[^ ^72^, ^73^, ^74^ ^]^ (i.e., sensor). By coupling actuation and sensing in a single material, the MGO platform enables enhanced multifunctionality for magnetoactive machines. Figure 7b illustrates the conceptual closed‐loop control enabled by the MGO sensoriactuator: magnetic fields are programmed to induce deformation, which is detected in real time through resistance changes in the GO layer. This feedback can then be used to dynamically adjust the actuation input, enabling an autonomous control over the system.

For experimental validation, an MGO beam is prepared with one end fixed to a fixture while the other end is kept free to bend. Two copper wires are attached to both ends of the GO layer using copper tape to allow resistance measurements. When the MGO beam is manually bent, a clear change in electrical resistance is observed (Figure 7c). Manual cyclic bending tests show reproducible electrical resistance change (Δ*R*/*R*_0_) synchronized with deformation cycles (Figure 7d), indicating a reliable strain sensitivity and good signal stability. Subsequently, the MGO beam is subjected to magnetic actuation using a Helmholtz coil setup. Under the applied magnetic field, the MGO beam bends, and the electrical resistance of the GO layer changes concurrently (Figure 7e,f). The resistance change signal closely tracks the bending cycle in both amplitude and timing, confirming the real‐time dual functionality of the MGO beam. Although preliminary, this demonstration highlights a key advantage of the MGO platform: self‐sensing actuation, where a single structure performs both actuation and sensing without the need for external sensors. This integrated functionality opens pathways for closed‐loop control, adaptive morphing, and autonomous soft robotic systems capable of real‐time feedback, self‐correction, and responsive behaviors in complex or dynamic environments.

## 3 Concluding Remarks

In summary, the developed MGO film represents a significant advancement in the field of responsive materials. By integrating hard‐magnetic microparticles into a GO substrate, lightweight MGO films demonstrate a range of functional properties, including magnetically‐driven rapid shape morphing, precise remotely‐controlled structural reconfigurations, and magnetization reprogramming for controllable magnetically‐actuated shape changing. These capabilities are complemented by material stability across various environmental conditions, including water. In addition, the simplicity and adaptability of MGO films facilitate the efficient on‐demand creation of complex reconfigurable architectures with programmable magnetization distributions tailored for specific application requirements. The ability to reprogram magnetization patterns enables real‐time shape morphing and integration of multiple functionalities in deployed systems. These properties make MGO films highly adaptable and suitable for a diverse range of applications.^[^ ^16^, ^17^, ^25^, ^32^, ^101^, ^106^, ^107^ ^]^ Moreover, the integration of magnetic actuation and strain sensing within an MGO sensoriactuator establishes a new class of self‐sensing actuators, paving the way for the closed‐loop control of adaptive soft robotic systems. With scalable and simple fabrication, and reconfigurable design, this work opens pathways toward MGO‐based self‐sensing robots of diverse shapes and sizes,^[^ ^33^, ^36^, ^79^, ^101^, ^107^ ^]^ from millirobots^[^ ^17^, ^25^ ^]^ to large‐size soft robots,^[^ ^71^, ^108^, ^109^ ^]^ with potential to improve the existing technologies with sensing and actuating characteristics.

## 4 Experimental Section

### Magnetic GO Film Preparation

1. *GO in RA suspension (GO/RA)*. GO paste, containing 25 wt.% of GO and 75 wt.% of water, was added to 40 mL RA to reach a GO concentration of 40 g L^−1^. GO was dispersed using a vortex shaker (Vortex‐Genie) (speed of 2700 rpm, 10 min). To remove water from the GO paste, the suspension was centrifuged (300 g force, 10 min) and the supernatant was discarded, followed by adding RA and re‐dispersing using the vortex shaker. This procedure was repeated three times. After the last time of centrifugation and removal of RA, GO was dispersed in RA using a vortex shaker to achieve a suspension with a volume of 39.2 mL. Then, 0.8 mL of glycerol were mixed with the suspension by shaking for 5 min using the vortex shaker.
2. *NdFeB/PAA in RA suspension (NdFeB/PAA/RA)*. PAA was dissolved in RA (20 g L^−1^); varying amounts of NdFeB particles were dispersed into the PAA solution through sonication for 5 min. NdFeB particle concentrations used to prepare different MGO films are shown in Table S1 (Supporting Information).
3. *MGO films*. MGO films with a diameter of 13 cm were prepared by mixing 3.5 mL of GO/RA suspension with 3.5 mL of NdFeB/PAA/RA using the vortex shaker for 10 min. The mixture was then cast onto the surface of a GO film prepared following a procedure previously described,^[^ ^66^ ^]^ in the presence of a PLA mold (Figure 1a‐iv). The resulting MGO film was obtained by air‐drying for 20 min. The mold was then removed, and the film was peeled off.

### Morphology and Composition Characterization

The cross‐section morphology of MGO films was characterized by SEM and X‐ray EDS mapping (Figure 1b; Figures S2–S6, Supporting Information) using FlexSEM 1000 SEM (Hitachi, Canada), under an accelerating voltage of 10 kV and a working distance of 10 mm. Thermogravimetric analysis (TGA) was performed using TGA Q50 (TA Instruments, Delaware, USA), by heating up the samples from room temperature to 800 °C at 20 °C min^−1^ under air purging (60 mL min^−1^).

### Mechanical Characterization

To measure the bending stiffness of MGO films, a three‐point bending test was conducted using an ADMET eXpert 7601 equipped with a 10 lbf load cell with 1 mm min^−1^ loading rate (Figure 2c; Figure S14, Supporting Information). Average values and standard errors were calculated using four samples for each MGO film type. To study the effect of moisture on the mechanical properties of MGO films, a uniaxial tensile test was performed using an ADMET eXpert 7601 equipped with a 250 lbf load cell with a loading rate set at 0.1 mm s^−1^. A dogbone sample with a gauge length of 20 mm and width of 2 mm was prepared by laser cutting (Speedy 100, Trotec Laser, CANADA) for tensile testing. Average values and standard errors were calculated using four samples for each MGO film type. To create low moisture condition (LM), the MGO dogbone samples were kept in a heating chamber at a temperature of 60 °C for 5 min to enable water desorption (Figure S19c, Supporting Information). For the room moisture (RM) condition, the MGO dogbone samples were kept in a desiccator with RH ≈55% (measured through a digital hygrometer with accuracy of ± 5 RH% (EEEKit, 750 958 488 428)) (Figure S19a, Supporting Information). For the high moisture (HM) condition, the MGO dogbone samples were kept in a sealed chamber with RH ≈70% for 2 min (Figure S19b, Supporting Information). Detailed steps can be found in Section S5D (Supporting Information).

### Fabrication of MGO Origamis

The MGO films were first perforated using a laser cutter (Speedy 100, Trotec Laser, CANADA. Laser power: 20 W; cutting speed: 3.5 inch/s) along prescribed patterns (e.g., six‐armed structures, Miura‐ori, Kresling, TMP, and jellyfish‐inspired designs). The creases were created as dashed lines, with fully perforated cut segments of length of 1.5 mm and spacing between each perforation of 1.5 mm. To fabricate the MGO Miura‐ori (Figure 1c‐i,d), Kresling (Figures 1c‐iii and 3b), TMP (Figures 1c‐iv and 3g), and Miura‐ori tube (Figures 1c‐*ii* and 4), the laser‐cut MGO films were manually folded along the designed “mountain” and “valley” creases. Double‐sided tape was used to assemble different parts (e.g., mirrored Miura‐ori strips in Figure S10b) to form Miura‐ori tube, TMP, and Kresling. In the magnetic‐sticker‐assisted magnetization programming/reprogramming strategy, PAA (10 g L^−1^) was used to bond GO and MGO (Figure 6; and Movie S8, Supporting Information). PAA solution was first applied to the surface of the GO or MGO films. After allowing ≈30 s for PAA to dry, the MGO stickers were carefully placed on the top of the GO or MGO films. Gentle pressure was applied to ensure strong adhesion. The PAA enables robust bonding between the films, while still allowing them to be easily detached for reprogramming when needed (Movie S8, Supporting Information).

### Magnetization

MGO structures were magnetized using a customized template‐aided magnetization method using an impulse magnetizer (Model DXMM‐20C70, Xiamen Dexing MagnetTech, China) to achieve the desired magnetization patterns. After charging the impulse magnetizer, a magnetic field exceeding 3T was applied to magnetize the NdFeB particles. The fixtures (templates) for magnetization were designed with diverse geometries and prepared by laser cutting (Speedy 100, Trotec Laser, CANADA) of plastic sheets (TroGlass, Trotec Laser, CANADA) and 3D printing (Ultimaker S3) of polyethylene terephthalate glycol (PETG). To apply the magnetization shown in Figures 3b,f, and 4c, two plastic sheets (TroGlass, Trotec Laser) were used to fix the samples and align them with the desired direction for magnetization. Origami designs (Miura‐ori, Miura‐ori tube, TMP, and Kresling) were magnetized in a fully folded state with the help of two plastic sheets. To apply the magnetization shown in Figure 3a‐iii–v, a hexagonal prism template (light blue hexagonal prism in Figure 3a‐ii) and a hollow hexagonal prism were used. The hollow hexagonal prism had an inner dimension slightly larger than the hexagonal prism template. Using these tools, the arms of the MGO structure were folded into the desired magnetization configurations (e.g., three arms up and three arms down, or all six arms down). This was achieved by placing the hexagonal prism template inside the hollow hexagonal prism, with the deformed MGO sample positioned between them, enabling precise magnetization.

### Magnetic Actuation

To magnetically actuate shape morphing and locomotion of MGO structures, a permanent magnet (NdFeB Magnet, McMASTER‐CARR) or a one‐axis Helmholtz coil system (Figure S13, Supporting Information Model DXHC17.5‐800, Xiamen Dexing MagnetTech, China) powered by a 6 kW DC power generator (Model DXKDP‐6000, Xiamen Dexing MagnetTech, China) were used to generate magnetic field. The magnitude of the uniform magnetic field generated by the one‐axis Helmholtz coil was controlled by programming the input current and was measured using a gaussmeter (Model DX‐150, Xiamen Dexing MagnetTech, China). The coil could produce a 2.7 mT A^−1^ uniform magnetic field, reaching up to 80 mT, with a spacing of 104 mm between the coils. The permanent magnet was moved by hand underneath the MGO structures to create spatially varying magnetic fields by combining vertical, horizontal, and rotational movements. The detailed movements of the permanent magnets used to actuate the MGO structures are shown in Figure 4d–f and Section S8 (Supporting Information). To study the effect of moisture on the magnetic actuation of MGO beams, the same methods described in the mechanical characterization section were applied to create the HM and LM conditions (details can be found in Section S54E, Supporting Information).

### Finite Element Analysis

The magneto‐elastic behavior of structures composed of MGO films was simulated using a quasi‐static finite element analysis with geometric nonlinearity carried out in COMSOL Multiphysics 5.5 (Section S10, Supporting Information). The coupled multiphysical problem, involving both elastic and magnetic domains, was simplified by assuming an ideal hard magnetic material model for the MGO film. This model assumes a constant remanent magnetization within the range of the actuating magnetic field and neglects the perturbation of the applied magnetic field caused by the presence of the hard magnetic particles. With this simplification, the magnetization effect can be represented as a deformation‐dependent surface boundary load per unit area. The MGO structure was then partitioned into different magnetic domains, each with its own distinctive magnetization direction, and the magnetic boundary load under the actuating magnetic field was applied to all boundaries enclosing a magnetic domain. For simulations, MGO was assumed to be nearly incompressible.

## Conflict of Interest

The authors declare no conflict of interest.

## Supporting information



Supporting Information

Supplemental Movie 1

Supplemental Movie 2

Supplemental Movie 3

Supplemental Movie 4

Supplemental Movie 5

Supplemental Movie 6

Supplemental Movie 7

Supplemental Movie 8

Supplemental Movie 9

Supplemental Movie 10

## Data Availability

The data that support the findings of this study are available from the corresponding author upon reasonable request.
