# Supplementary material for: Multifunctional and Reprogrammable Magnetoactive Graphene Oxide Origami
Source: Adv Sci (Weinh). 2025 Oct 30;13(1):e14597. doi: 10.1002/advs.202514597 (PMC12767127; doi:10.1002/advs.202514597)
Supplement: Supplementary file 1 — Supporting Information [file ADVS-13-e14597-s011.pdf]

Supporting Information

Multifunctional and Reprogrammable Magnetoactive Graphene Oxide Origami

Jun Cai[a]†, *Yiwen Chen*[b]†, *Alireza Seyedkanani*[a], *Guocheng Shen, Marta Cerruti*[b*], *Abdolhamid Akbarzadeh*[a,c**]

[a] Department of Bioresource Engineering, McGill University, Montreal, QC H9X 3V9, Canada
[b] Department of Mining and Materials Engineering, McGill University, Montreal, QC H9A 0C3, Canada
[c] Department of Mechanical Engineering, McGill University, Montreal, QC H3A 0C3, Canada

[*] Email: marta.cerruti@mcgill.ca
[**] Email: hamid.akbarzadeh@mcgill.ca
† Equal contribution

S1. Chemicals and Materials

Graphene oxide (GO) paste (25 wt%) was purchased from LayerOne and was synthesized by a modified Hummers' method (product number: 12001). Neodymium-iron-boron (NdFeB) particles (average diameter ~ 5 μm, containing 19.1 wt% neodymium, 6.3 wt% praseodymium, 0.9 wt% boron, and 73.7 wt% iron) were purchased from Neo Maqnequnch (product number: 20441-089). Poly(acrylic acid) (PAA, Mv ~450,000), calcium chloride dihydrate ($CaCl_2$, 99%), glycerol (99.5%), magnesium nitrate hexahydrate ($MgN_2O_6 \cdot 6H_2O$, 99%) were purchased from Sigma-Aldrich. The cellulose filter papers (Fisherbrand™ Qualitative Grade Plain Filter Paper Circles - P8 Grade, diameter = 15 cm) were purchased from Fisher Scientific. Reagent alcohol (RA, containing 88 to 91 % ethyl alcohol, 4 to 5% methyl alcohol, and 4.5 to 5.5% isopropyl alcohol) was purchased from Fisher Chemical (LOT 222195).

S2. MGO Preparation and Characterization

The GO film is prepared following the procedure outlined in our previous work [1]: briefly, a cellulose filter paper (diameter = 15 cm) is placed on a polyester substrate (30.5*22.9 cm^2), on a horizontal bench. 3.5 mL $CaCl_2$ solution (0.32 g/L) are drop-casted onto the cellulose filter paper. A 3D printed polylactic acid (PLA) mold (diameter = 13 cm) is placed and pressed against the wet cellulose paper to ensure good adherence between the paper and the mold. Then, 52 mL GO/$CaCl_2$ suspension (GO concentration of 10 g/L and $CaCl_2$ to GO weight percentages of 3.2 wt%) are drop-casted onto the wet cellulose filter paper within the mold. The film is dried at relative humidity around 54% for four days before the addition of GO/NdFeB/PAA suspensions onto the GO film (Fig. 1a (*iv*) in the main text). After drying, a freestanding MGO film consisting of the Ca-crosslinked GO film with on top a magnetic GO/NdFeB/PAA layer is obtained by removing the mold and peeling off the film from the cellulose paper. The thickness of the MGO films characterized by different particle weight concentrations is determined by SEM (Fig. 1b in the main text, and Figs. S2-S6) and reported in Table S2.

The amount of NdFeB loaded in the MGO films is evaluated by thermogravimetric analysis (TGA) (Figure S1). When heated in air flow, the NdFeB particles gain weight continuously starting from 400 ºC due to oxidation, reaching a plateau corresponding to 36.6% weight gain at 780 ºC because of completed oxidation [2-4]. On the other hand, GO undergoes a stepwise weight loss, related to first water desorption, functional group decomposition, and then complete decomposition in air above 620 ºC (Figure S1). The weight of MGO films decreases following the same steps observed for GO but does not go to zero after 620 ºC due to the formation of stable oxides from the NdFeB particles. Therefore, the weight fraction (ϕ, wt%) of NdFeB in different MGO films can be estimated using the following equation:

$$\phi = \frac{R_{MGO}}{R_{NdFeB}} \times 100\% \qquad \text{(Eq. S1)}$$

where R_{MGO} and R_{NdFeB} represent the residue weight percentages at 800 ºC of MGO film and NdFeB particles, respectively. As the results shown in Table S2, the obtained MGO films are named as MGO1, MGO2, MGO3, MGO4, MGO5, and MGO6, with the weight concentration increasing progressively

from MGO1 to MGO6. The maximum NdFeB weight fraction obtained in MGO6 corresponds to 31 wt%.

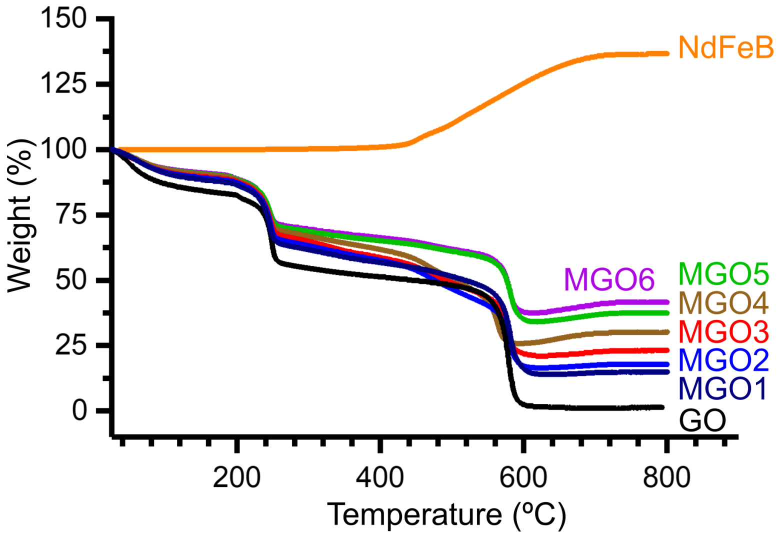

Figure S1. Thermogravimetric analysis (TGA) curves of NdFeB, MGO1 to MGO6 films, and GO films.

Table S1. NdFeB concentration in the NdFeB/PAA in RA suspensions for different MGO films. The limit for NdFeB concentration is 113 g/L, beyond which particle precipitation occurs.

MGO film	NdFeB concentration (g/L)
MGO1	25
MGO2	37
MGO3	52
MGO4	70
MGO5	90
MGO6	113

Table S2. Residue weight percentage (wt%) of NdFeB and MGO films measured by performing TGA in air up to at 800 ºC, and the corresponding estimated NdFeB weight fraction in the MGO films (*ϕ*). The thicknesses of MGO1 to MGO6 are determined by SEM and represented as the mean ± standard deviation.

Sample	Residue (wt%)	*ϕ* (wt%)	Thickness (μm)
NdFeB	137	–	–
MGO1	15	11	29.6 ± 0.2
MGO2	18	13	30 ± 2
MGO3	23	17	32 ± 2
MGO4	30	22	33 ± 1
MGO5	38	28	36 ± 1
MGO6	42	31	39 ± 2

Table S3. Comparison of estimated surface density (mg/cm^2), specific surface areas (cm^2/g) and multifunctional characteristics among representative magnetoactive composites. Surface densities for literature examples are approximated based on the reported matrix materials (excluding fillers), which are estimated using $\rho \times t$, where ρ is the density of the matrix and t represents the thickness of the fabricated magnetoactive films or composites.

Matrix	Magnetic Filler	Surface density (mg/cm^2)	Functionalities	References
Graphene oxide (This work)	NdFeB	6.26	Magnetic actuation, Humidity tuning, Sensing, Magnetization reprogramming	This work
Silicone elastomer	$SrFe_{12}O_{19}$, NdFeB	21.6 – 122.1	Magnetic actuation only	[5-17]
Silicone elastomer + Phase change polymer	NdFeB	107.99 – 122.1	Magnetic actuation, Magnetization reprogramming	[18]
Shape memory polymer (SMP)	NdFeB and Fe_3O_4	10 – 17.7	Magnetic actuation, Magnetization reprogramming, Shape-locking, Sensing (additional sensor foil)	[19, 20]
Polydimethylsiloxane (PDMS)	NdFeB	7.21 – 338.98	Magnetic actuation, Biocompatible, Thermal actuation, Sensing (additional silver nanowires)	[21-29]
PDMS	CrO_2	20.6 – 22.6	Magnetic actuation, Magnetization reprogramming	[30]
Hydrogels	NdFeB, Iron oxide	100 – 165.02	Magnetic actuation, Light actuation, Biocompatible, Biodegradable	[31-33]
Squid ring teeth (SRT) protein	NdFeB	–	Magnetic actuation, Self-healing	[34]
Silicone + Polycaprolactone	NdFeB	12 – 14.64	Magnetic actuation, Magnetization reprogramming	[35]
Cellulose paper	NdFeB	7.2 – 124.07	Magnetic actuation only	[36-39]
liquid crystalline elastomer	Fe_3O_4	–	Magnetic and thermal actuations	[40]
Tapioca starch + GO	Fe_3O_4	–	Humidity and magnetic actuation	[41]

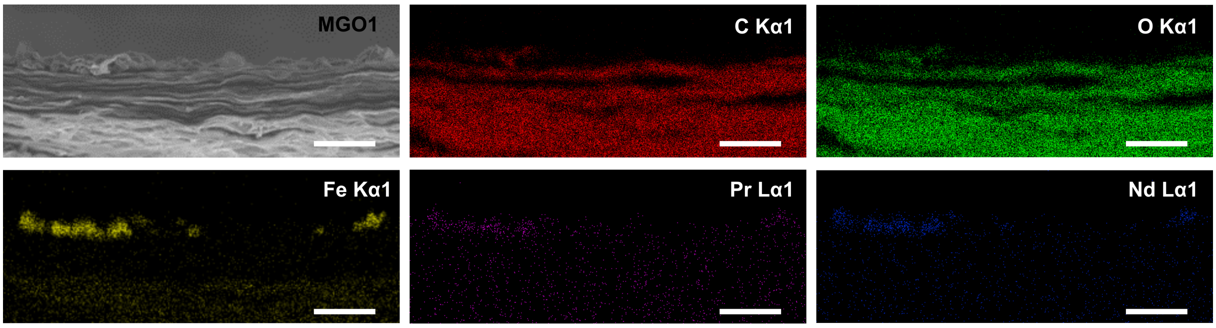

Figure S2. SEM and EDS elemental mapping images of an MGO1 film (ϕ = 11 wt%). Scale bar = 25 µm.

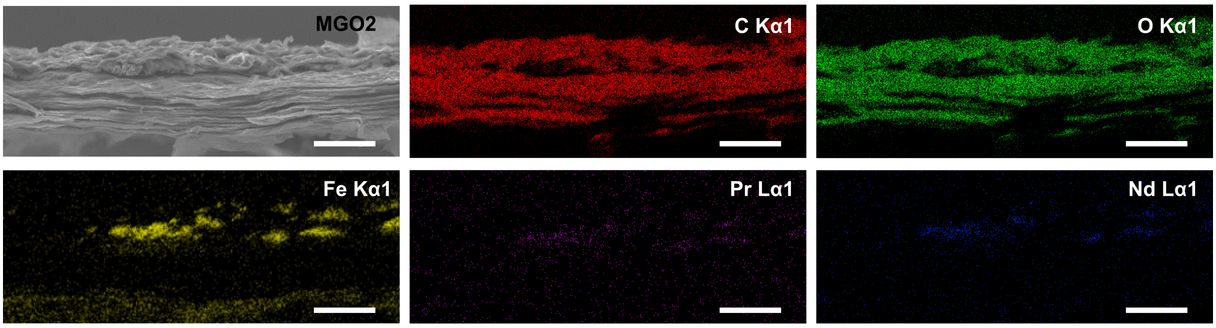

Figure S3. SEM and EDS elemental mapping images of an MGO2 film (ϕ = 13 wt%). Scale bar = 25 μm.

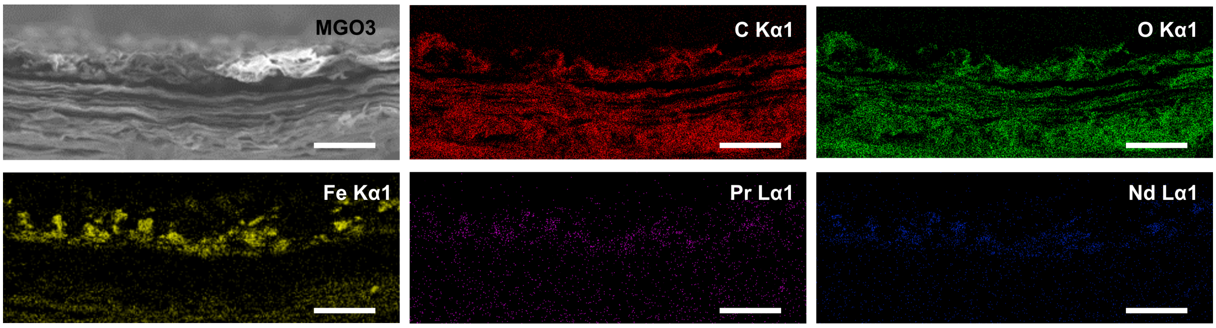

Figure S4. SEM and EDS elemental mapping images of an MGO3 film (ϕ = 17 wt%). Scale bar = 25 μm.

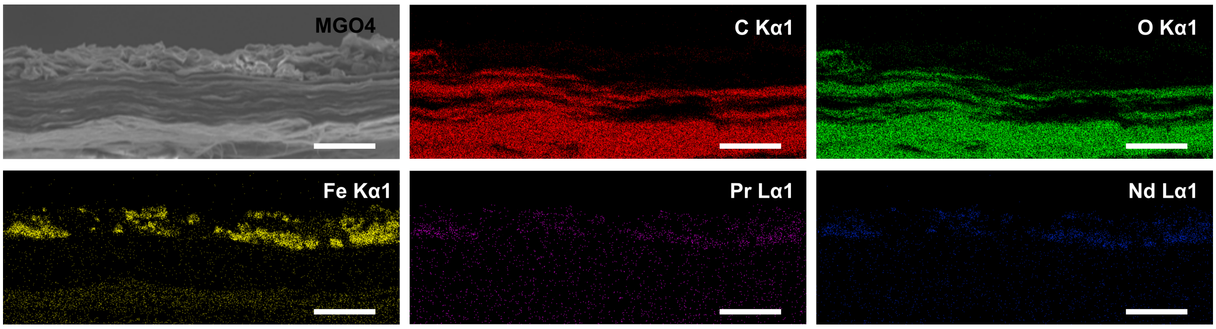

Figure S5. SEM and EDS elemental mapping images of an MGO4 film (ϕ = 22 wt%). Scale bar = 25 μm.

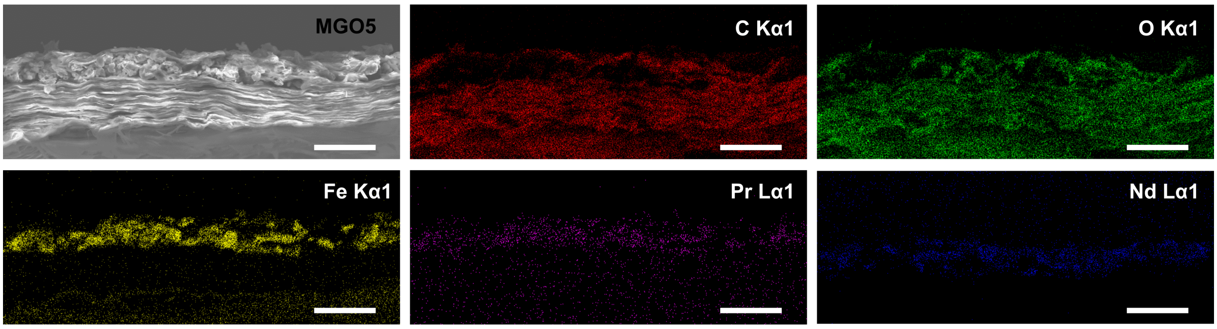

Figure S6. SEM and EDS elemental mapping images of an MGO5 film (ϕ = 28 wt%). Scale bar = 25 μm.

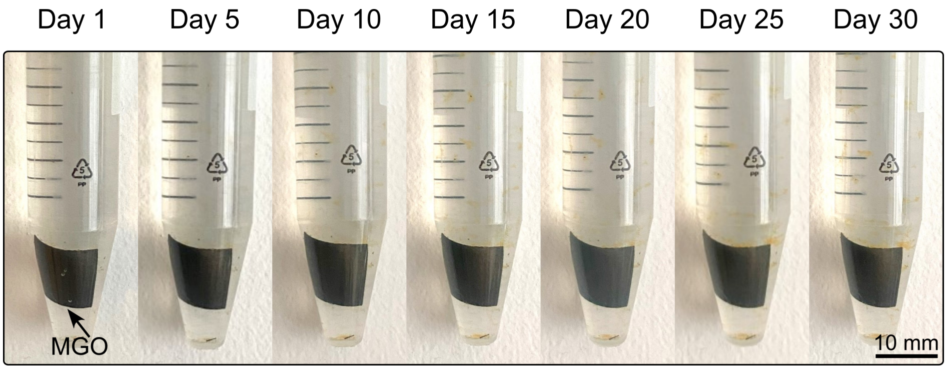

Figure S7. MGO6 film is stable in water for at least 30 days.

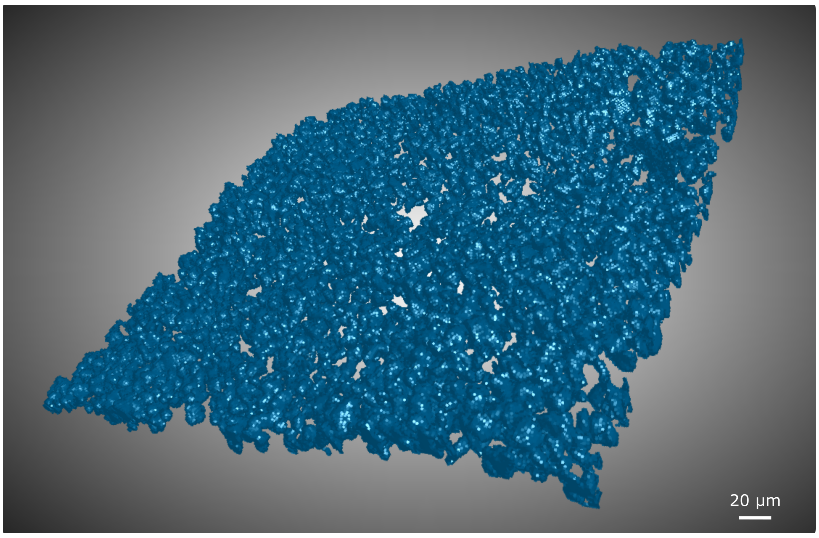

Figure S8. Micro-computed tomography image of MGO sample. A micro-computed tomography specimen scanner, Zeiss Xradia Versa 520 (Zeiss, Jena, Germany), was used to explore the magnetic distribution on the MGO sample. The image acquisition was performed with a voltage of 80 kV and a current at 87 µA, ensuring a light transmission of 30% – 40%, as well as an LE1 filter provided by Zeiss. The micro-computed tomography images had a resolution of 364 pixels × 354 pixels, and the pixel size was 1.03 µm. The exposure time was 7 s per step. Dragonfly (educational version) was used to perform the subsequent reconstruction.

S3. Oxidation/Corrosion Effects on Magnetic Proeprties

In our study, magnetic actuation experiments were performed in water immediately after fabrication. Under these short-term conditions, no obvious oxidation was observed. However, NdFeB is intrinsically prone to oxidation and corrosion in humid or aqueous environments, particularly under acidic (low-pH) environments, leading to rapid deterioration of magnetic properties such as remanence and coercivity [42-45]. For example, uncoated NdFeB particles exposed to an acidic environment (pH ≈ 1.35) for 24 h showed a decrease in saturation magnetization from ~111.5 emu/g to ~98 emu/g [44]. Severe degradation is also observed under high humidity conditions (95% RH and 80 °C for 100 h), saturation magnetization dropped from ~111.5 emu/g to ~32 emu/g in uncoated samples [44].

To evaluate the practical stability of our MGO samples, we tested MGO6 beam samples stored in sealed polyethylene bags for ~19 months. It is worth mentioning that these samples had previously been tested across different humidity levels (low, room, and high moisture conditions, Figs. 1e and 1f; cyclic adsorption/desorption, Fig. 2g in the manuscript). When retested after long-term storage, the MGO6 beams showed no obvious decrease in actuation performance, as the normalized deflection at $B_{applied}$ = 70 mT remained nearly identical (Fig. S9). This demonstrates that proper storage can effectively preserve magnetic performance over extended periods.

Nevertheless, to ensure long-term stability of NdFeB-based actuators in humid or aqueous conditions, several protective strategies have been demonstrated in the literature: (1) *Surface modification*. Phosphoric acid treatment forms protective layers, reducing coercivity loss from > 40% to ~1% after high-temperature/humidity exposure [46]. (2) *Adhesive coatings*. Thin epoxy coatings (e.g., 3M Scotch-Weld DP100 and J-B Weld epoxy coating) [44, 47-49] effectively block acidic attack, preserving saturation magnetization (~113 emu/g) compared to severe degradation (~32 emu/g) in uncoated samples [44] under high humidity. (3) *Electrophoretic deposition and composite epoxy coatings*. Epoxy films [49, 50], especially those filled with Zn or Al powders [51], provide durable protection. For example, Zn/epoxy coatings maintained magnetic properties and showed no visible corrosion after ~1000 h of neutral salt-spray exposure [51]. (4) *Metallic barrier or sacrificial coatings*. Metallic coatings such as Al or Zn/Al films serve as sacrificial barriers, lowering corrosion rates by nearly an order of magnitude, thereby extending the stability of magnetic properties [52, 53].

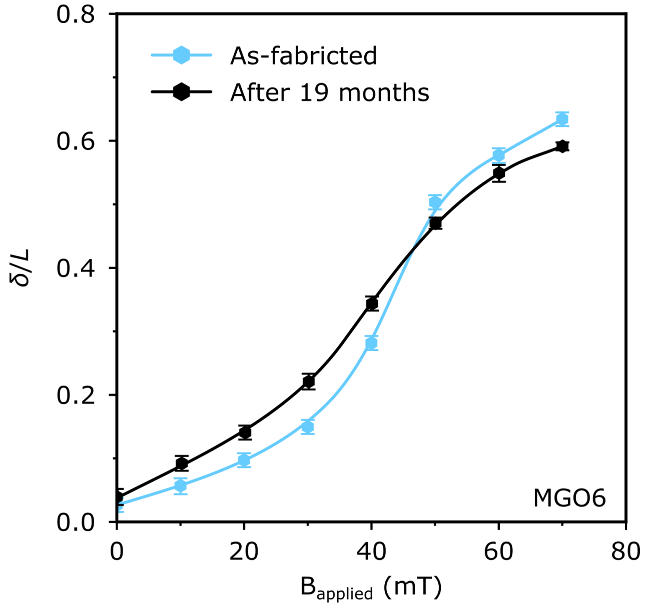

Figure S9. Normalized free-end deflection of MGO beams as a function of applied magnetic field. The blue curves represent the samples tested immediately after fabrication and magnetization, while black curve represents the same samples tested after 19 months of storage.

S4. MGO Miura-ori

A. Pattern geometry. The Miura-ori origami is composed of parallelogram panels connected by edges that can be folded into "mountain" and "valley" creases (Fig. S10) [54]. The unit cell of Miura-ori, as shown in Fig. S10a, consists of four identical parallelograms with sides of length a and b, and an acute angle θ between them. The Miura-ori tube is constructed by assembling two mirrored Miura-ori strips, enabling foldability in two directions (Fig. S10b) [55, 56]. Fig. S10c shows Miura-ori and Miura-ori tube origamis fabricated by MGO films. All Miura-ori structures and Miura-ori tubes in this study are characterized by $a = b = 10$ mm and $\theta = 60°$. The creases in the MGO Miura-ori and Miura-ori tube are created by laser cutting (refer to the Experimental Section in the main text).

B. Kinematics. Assuming the panels are rigid in comparison to the creases, the Miura-ori exhibits only one degree of freedom. The dimensions of the Miura-ori structure, as illustrated in Fig. S10b, can be expressed as [54, 57, 58]

$$l = 2b\sin(\beta/2) \quad \text{(Eq. S2)}$$

$$w = 2a\frac{\cos\theta}{\cos(\beta/2)} \quad \text{(Eq. S3)}$$

$$h = \frac{a\sqrt{\sin^2\theta - \sin^2(\beta/2)}}{\cos(\beta/2)} \quad \text{(Eq. S4)}$$

$$\cos\alpha_1 = 1 - \frac{2\sin^2(\beta/2)}{\sin^2\theta} \quad \text{(Eq. S5)}$$

$$\cos\alpha_2 = 1 - 2\cot^2\theta\tan^2(\beta/2) \quad \text{(Eq. S6)}$$

where $\beta \in [0^\circ, 2\theta]$ represents the angle between two valley creases, and $\beta = 0^\circ$ and 2θ correspond to the completely collapsed state and planar state, respectively.

C. Magnetic Actuation of MGO Miura-ori. With a template-assisted magnetization strategy, more complex magnetization patterns can be programmed, yielding complex 3D shapes under an applied magnetic field. As an example, we design a Miura-ori pattern with programmed magnetization patterns (Fig. 1d (*i*) in the main text). To achieve this magnetization pattern, the MGO Miura-ori is magnetized in its fully folded state, with the magnetic field applied parallel to the Miura-ori panels. MGO Miura-ori demonstrates rapid folding/unfolding under an applied magnetic field, either a uniform field generated by a Helmholtz coil or a non-uniform magnetic field applied by a permanent magnet (NdFeB Magnet, McMASTER-CARR; Movie S2). The larger folding observed in response to the permanent magnet indicates that a higher magnetic field magnitude is generated by the permanent magnet. MGO Miura-ori exhibits a larger deformation in water than in air (Fig. S11 and Movie S2). This is likely because the MGO creases become softer when exposed to water, as they absorb water.

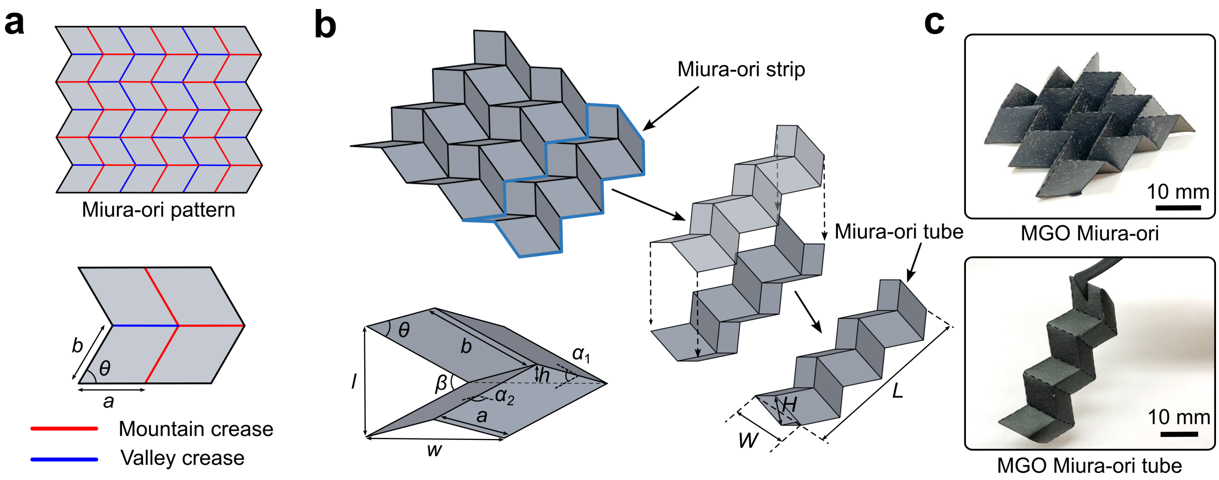

Figure S10. **(a)** Miura-ori pattern. $a = b = 10$ mm and $\theta = 60^{\circ}$. **(b)** Fabrication of Miura-ori tube and the geometry of Miura-ori unit cell. **(c)** Digital pictures of Miura-ori and Miura-ori tube fabricated by MGO films.

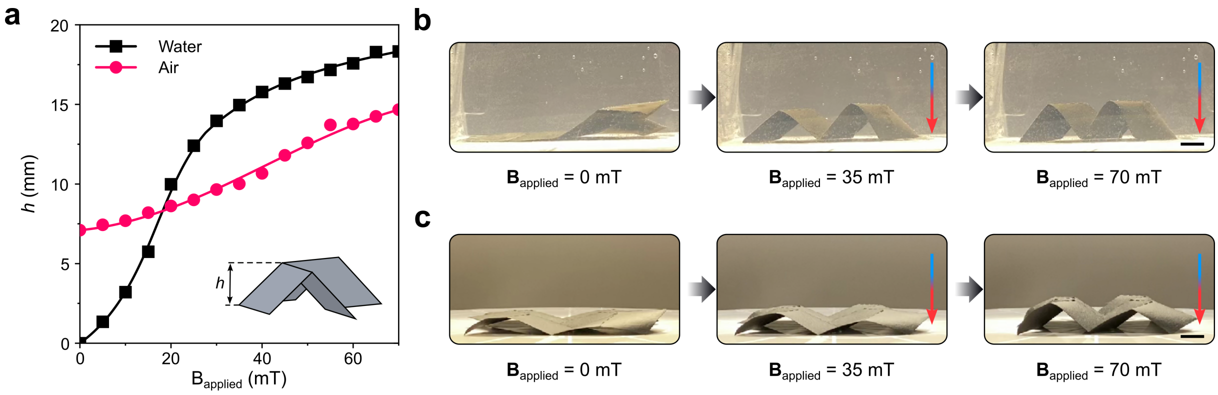

Figure S11. **(a)** Height of the MGO Miura-ori structure as a function of applied magnetic field in water and in air. Digital pictures of magnetic field-induced shape morphing of MGO Miura-ori in **(b)** water and **(c)** in air.

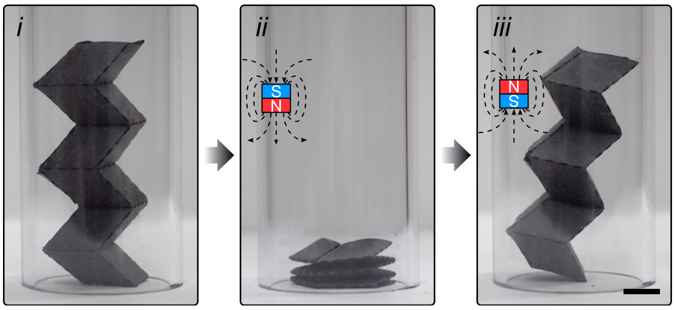

Figure S12. Reversible contraction and expansion of MGO Miura-ori tube under a magnetic field generated by a permanent magnet. Scale bar: 10 mm.

S5. Magnetic Actuation Experiment

A. Helmholtz Coil and Permanent Magnet. The MGO samples are actuated within a Helmholtz coil system (Model DXHC17.5-800, Xiamen Dexing MagnetTech, China) powered by a 6 kW DC power generator (Model DXKDP-6000, Xiamen Dexing MagnetTech, China). This Helmholtz coil system can generate a one-dimensional (1D) uniform magnetic field (Fig. S13a). The magnitude of the magnetic field can be precisely controlled by adjusting the electric current in the coil. The coil can produce a 2.7 mT/A uniform magnetic field, reaching up to 80 mT, with a spacing of 104 mm (i.e., spacing between the coils). For the non-uniform magnetic field generated by a permanent magnet, several well-estimated methods are utilized to characterize the magnetic fields: (1) *Gauss meter mapping* [59-61]: A Hall probe (axial or transverse) is placed at a known location on or near the magnet's surface. At each point, the Hall sensor records the magnetic flux density (B), which is proportional to the voltage induced in the sensor. Accurate mapping requires careful alignment of the probe's orientation and tip position. The collected discrete data is then used to construct continuous 2D contour maps or full 3D vector field distributions, which reveal both the field magnitude and the gradient. This method is relatively simple, widely accessible, and can be validated by repeating measurements at reference locations. However, it is time consuming for high-resolution mapping. (2) *Advanced magnetic field scanning system*. To overcome the limitations of manual point-by-point mapping, commercial systems such as the Magcam Combi Scanner have been developed. This system with a 4-axis motorized scan state can measure the magnetic field distributions of permanent magnets in different types, shapes, and sizes (more than 16000 measurement points). The output is a full 3D magnetic flux density map that includes field magnitude, direction, and gradients. (3) *Finite element modeling tools* [62-64]. Finite element methods (e.g., COMSOL Multiphysics and Finite Element Method Magnetics) are widely used to simulate spatially varying magnetic fields. In these approaches, the magnet geometry and material properties (e.g., remanent magnetization) are defined, and the magnetostatic equations are solved. The computed field maps and gradients can then be exported for analysis or used directly to predict forces and torques on magnetoactive structures. These computed gradients are often validated experimentally and then used to predict the deformations of structures.

B. Magnetization of MGO beam. To characterize the actuation performance of MGO films, rectangular MGO samples (40 mm × 10 mm) are first prepared and magnetized in a uniform magnetic field exceeding 3 T (Model DXMM-20C70, Xiamen Dexing MagnetTech, China) oriented along the sample length, as indicated by the white arrow in Fig. S13b. The magnetization process is completed in a very short time, taking less than one second.

C. Cantilever Bending Actuation. The MGO samples are attached to a polyethylene terephthalate glycol (PETG) fixture (SKU: PT1004TQ, Kimya) that constrains one end of the MGO sample while the other end remains completely free (Fig. S13b). The surface of the MGO samples is perpendicular to the magnetic field direction. To eliminate the contribution of gravity, the surface of the MGO beam and the uniform magnetic field direction are perpendicular and parallel to the ground, respectively (Fig. S13b),

so that the induced magnetic torque leads to the MGO sample bending parallel to the ground. The magnetic field magnitude increases from 0 mT to 70 mT at a rate of 4.67 mT/s. The deflection of the MGO sample tip under different magnitudes of magnetic field is then recorded by the camera (Fig. S13a).

The deflection observed as a function of magnetic field is non-linear (Fig. 2b in the main text). Under a uniform magnetic field, the generated distributed torque magnitude ($d\tau$) is given by

$$d\tau = \left|\mathbf{M} \times \mathbf{B}_{\text{applied}}\right| dv \quad \text{(Eq. S7)}$$

where $\mathbf{M}$ is the magnetization of the MGO beam, $\mathbf{B}_{\text{applied}}$ is the applied magnetic field, and dv is an infinitesimally small volume element. Initially, the magnetic field is perpendicular to the magnetization; however, the angle decreases with increasing applied magnetic fields due to bending of the GMO beam. Consequently, the magnetically induced torque varies as the MGO beam bends, resulting in a nonlinear relationship between the deflection and magnetic field magnitudes. This can be clearly seen by comparing the behavior of MGO beams with high vs. low ϕ values. The response of MGO6 (ϕ = 31 wt%) is characterized by three distinct regions (Fig. 2b in the main text): i) an initial linear response at low magnetic field magnitudes; ii) a sudden transition to a large change in deflection; iii) a final minimal increase in deflection due to the decreased magnetic torque resulting from the small angle between the magnetic field and the magnetization. In contrast, for MGO films with lower particle weight concentrations, the deflection under the uniform magnetic field is small, and the relative angle between the magnetization and magnetic field remains large, so that the third response region cannot be reached.

To better quantify the trade-off between magnetization and stiffness, we analyzed the deflection of uniformly magnetized MGO beams using scaling relations. Assuming the magnetization M of the MGO film is proportional to the particle weight fraction ϕ [65]:

$$M = \phi M_{\mathrm{P}} \quad \text{(Eq. S8)}$$

where M_{P} represents the magnetization of an individual particle. For an MGO beam subjected to small bending under a magnetic field, the deflection δ can be described by the Euler-Bernoulli beam theory:

$$\frac{\delta}{L} \sim \left(\frac{MB}{E}\right)\left(\frac{L^2}{\mathrm{I}}\right) \quad \text{(Eq. S9)}$$

where B is the applied magnetic field, L is the beam length, E is the Young's modulus, and $\mathrm{I} \sim t^3 b$ is the area moment of inertia (t and b are beam thickness and width). The dimensionless parameter MB/E characterizes the competition between magnetic torque and elasticity of the MGO film, serving as a figure of merit for actuation performance. Since the thickness changes due to particle addition is small and the magnetoactive layer alone cannot form a freestanding film, we assume the thickness is identical in MGO beams and approximate $E \sim K$ (bending stiffness, Fig. 2c of the manuscript). Accordingly, Eq. S8 can be simplified to

$$\frac{\delta}{L} \sim \left(\frac{MB}{K}\right) \quad \text{(Eq. S10)}$$

Experimentally, we found that the bending stiffness K increases nonlinearly with ϕ (Fig. 2c of the manuscript). Note that our MGO films consist of two layers: a pure GO layer and a magnetoactive layer containing NdFeB particles. For MGO films with different particle loadings, the GO base layer remains unchanged, while only the magnetoactive layer varies in particle content. In addition, due to the large spacing between GO interlayers, we use weight fraction rather than volume fraction to characterize the particle content. Hence, the nonlinear relationship between the K and ϕ can be fitted using a Mooney-type model [66, 67]:

$$K = K_0 \exp\left(\frac{2.5\phi}{1-1.35\phi}\right) \tag{Eq. S11}$$

where K_0 represents the bending stiffness of the pure GO film (see Fig. S15a). Substituting Eqs S8 and S11 into Eq. S10, we can get

$$\frac{\delta}{L} \sim \left(\frac{\phi M_{\mathrm{P}} B}{K_0 \exp\left(\frac{2.5\phi}{1-1.35\phi}\right)}\right) \tag{Eq. S12}$$

Based on Eq. S12, the optimal particle loading around 20 wt% (see Fig. S15b), which aligns with our experimental observation that MGO4 (22 wt%) achieves the best balance of torque generation and stiffness among the fabricated MGO films.

D. Frequency-dependent actuation performance. we conducted cantilever bending experiments on the MGO6 beams (experimental setup identical to Fig. 2a in the manuscript) under a uniform magnetic field at different frequencies ranging from 0.05 Hz to 1 Hz. The frequency was varied by adjusting the time required for the magnetic flux density to increase from 0 to 70 mT and then return to 0 mT. Based on the results shown in Fig. S18, we can conclude that: (1) At low frequencies, the normalized deflection-magnetic field curves nearly overlap, indicating that the actuation behavior is unaffected by the magnetic field frequency (Fig. S18a). (2) The actuation speed increases with the increased magnetic field frequency (Fig. S18b). (3) As the frequencies increase (e.g., 0.5 Hz and 1 Hz) the maximum normalized deflection decreases (Fig. S18c). For example, at B_{applied} = = 70 mT, the normalized deflection is ~0.6 at low frequencies but falls below 0.45 at 1 Hz. The experimental results indicate that while higher frequencies increase the actuation speed, the deformation amplitude is reduced due to the dynamic lag effect. We believe two main factors may contribute to this reduction in performance: (1) a dynamic lag effect, where the material deformation cannot fully keep pace with the rapidly increased magnetic field; and (2) limitations of the Helmholtz coil and its power supply. During high-frequency tests, we observed that the electric current displayed on the monitor did not reach the set value within very short actuation times (e.g., 0.5 s). This may either reflect the limited response time of the monitoring system or, more fundamentally, the inability of the power supply to deliver the required current (around 27 A) within such a short duration. These results highlight a general trade-off: *Low-to-moderate frequencies*: deformation amplitude is maintained, and faster cycling primarily increases the actuation

speed. *High frequencies*: deformation speed continues to increase, but the deformation amplitude decreases, reducing the degree of actuation [22, 68, 69].

This trade-off is also important for the locomotion of magnetic soft robots. For example, in the magnetically actuated fiber-based soft robots. Higher frequency actuation increases cycle numbers and can enhance crawling velocity, but friction and viscoelastic lag reduce the effective deformation, leading to a plateau in locomotion speed [69]. Another example of a two-arm trapezoid-shaped soft robot, high frequency magnetic actuation, although increase the deformation speed, reduces the bending deformation [22]. The phenomena observed in the locomotion/deformation of soft robots under varying actuation frequencies is consistent with the conclusions drawn from our experiments (Fig. S18).

E. Moisture Effect on Magnetic Actuation. To study the effect of moisture on the magnetic actuation of MGO beams, three distinctive moisture conditions are considered: high moisture (HM), room moisture (RM), and low moisture (LM). For RM, following the fabrication of the MGO film, the MGO samples are stored in a desiccator with a $MgN_2O_6 \cdot 6H_2O$ saturated solution that allows maintaining a relative humidity (RH) of approximately 55%, as measured by a hygrometer (EEEKit, 750958488428) (Fig. S19a). For HM, boiling water is initially placed in a sealed chamber to elevate the RH (Fig. S19b). Once the RH surpasses 70%, the MGO sample is placed inside the chamber for two minutes, allowing it to absorb moisture (Fig. S19b). For LM, the MGO sample is placed in a heating chamber at a temperature of 60 ˚C for five minutes to reduce its moisture content (Fig. S19c). After humidifying or dehydrating the MGO samples, magnetic actuation tests on the MGO beams are conducted immediately in room humidity (RH ~ 30%) and at room temperature (~23 °C). The same procedure is followed for the mechanical testing of MGO dogbone samples under RM, LM, and HM conditions.

To test whether the actuation performance of MGO beams is repeatable, we first subject an MGO sample to magnetically-driven deflection under HM conditions (see black curve in Fig. 2g in the main text); we then heat it up to 60 ℃ to release water, re-expose it to a high level of humidity by locating it in a humid chamber (RH > 70%) for two minutes, and finally perform the magnetically-driven deflection again under HM conditions (see pink curve in Fig. 2g). The results, as shown in Fig. 2g, confirm the consistency of MGO actuation performance regardless of the previous moisture conditions.

To better explain the moisture effect on MGO, we perform molecular dynamics (MD) simulations focusing on GO layer stacking with different water contents. Because the GO layers dominate the hygroscopic response, we modeled four stacked GO sheets (2.42 nm × 4.18 nm each) with only carbon atoms in sp^2 geometry. Epoxy (~10%) and hydroxyl (~15%) functional groups were randomly distributed on both sides of the graphene sheets, and the resulting GO sheets were initially separated by a distance of 0.7 nm [70]. Water molecules were randomly inserted between the sheets to create systems with varying water contents. MD simulations were conducted using the large-scale atomic molecular massively parallel simulator (LAMMPS) [71]. The Open Visualization Tool (OVITO) [72] was employed to visualize the evolution of atomic structures. The ReaxFF reactive force field [73, 74] was used in this MD simulation, as it provides bond breaking and bond forming capabilities and has been

widely used for studying GO and GO-water systems [70, 75-77]. The potential parameters applied in this study were adopted from Ref. [73]. The integration time step was set as 0.025 fs. Periodic boundary conditions were applied in all three directions. After energy minimization with the conjugate gradient algorithm, the GO-water system was first equilibrated with the isothermal-isobaric (NPT) ensemble at a constant temperature of 10 K and zero pressure for 625 fs. The structure was then gradually heated from 10 to 1000 K over 625 fs, maintained at 1000 K for 625 fs, and subsequently quenched to 300 K over a time span of 625 fs. Finally, the GO-water molecule system was equilibrated in the NPT ensemble at a constant temperature of 300 K and zero pressure in all three directions for 4.25 ps. After the simulation reached equilibrium, the spacing between the four GO layers was measured in the supercells containing different water contents.

The MD simulation results in Fig. S22 confirm that increasing water content expands the interlayer spacing: from 4.48 Å (0 wt% water) to 7.54 Å at 30 wt% water, consistent with previous experimental and simulation studies [70, 75, 78]. According to the van der Waals force (*F*) between two planar surfaces [78]:

$$\frac{F}{S} = \frac{A_{132}}{6\pi d^3} \quad \text{(Eq. S13)}$$

where A_{132} is the Hammaker constant for surfaces “1” and “2” interacting across medium “3”, d is the separation distance between the surfaces “1” and “2”, and S is the area of the surface. In our system, “1” and “2” represent GO, and “3” represent water. Based on MD simulation, when the water content increases from 10 wt% to 30 wt%, the *d*-spacing grows from 5.03 Å to 7.54 Å, reducing the interlayer van der Waals force per unit area to 29.7% of the original force (i.e., GO with 10 wt% water).

F. Magnetic Actuation of creased MGO. The fabrication of MGO origamis involves introducing creases. Typically, in origami, the creases are softer than the panels, which is especially evident in rigid origami where the panels remain rigid during folding and unfolding, and strain energy is stored primarily in the creases [79]. In our case, the shape change of MGO origami is actuated by the magnetic field, with magnetic torque playing a crucial role in the deformation process. As mentioned in Section 2.1 in the main text, the magnetic actuation performance of MGO beam is determined by the trade-off between increasing magnetic torque and bending stiffness as a function of magnetic particle loading. Due to magnetic torque, the magnetization of the creased MGO beam tends to align with the magnetic field, resulting in bending. Different from the bending of the MGO beam observed in the Figs. 2a and 2f in the main text, the deformation of creased MGO beams under a magnetic field transition from a pure bending to a torsion-spring-like folding mechanism, where the deformation is concentrated at the crease, while the MGO panels remain relatively rigid.

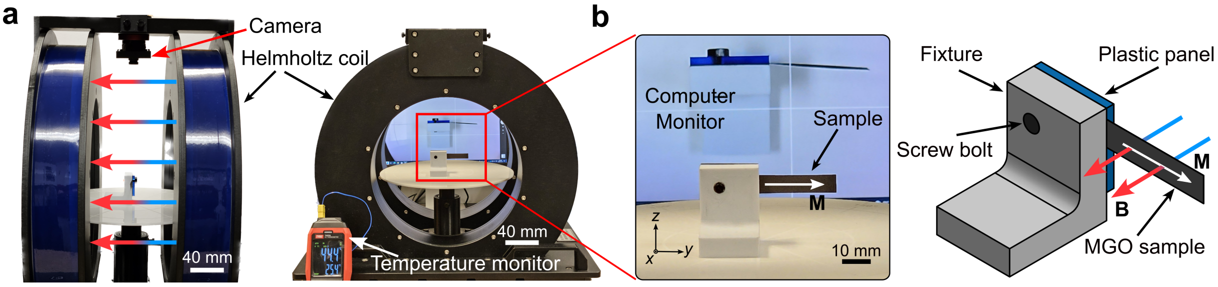

Figure S13. (a) Front and left views of the Helmholtz coil. A camera captures the magnetically-actuated deflection of the MGO beam. A temperature monitor is used to measure the coil temperature, preventing potential damage to the Helmholtz coil due to excessive heat. **(b)** Experimental setup for the cantilever bending actuation of MGO under a uniform magnetic field. A uniform magnetic field is applied along the *x*-direction, perpendicular to the magnetization direction of the MGO sample. The white arrow and colored arrows represent the magnetization (M) and the applied magnetic field (B), respectively.

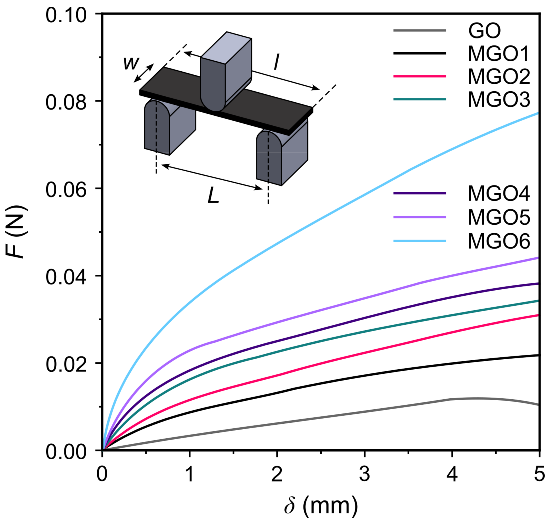

Figure S14. Force-displacement responses of MGO samples with different particle weight concentrations under three-point bending. The MGO sample dimensions are set to L = 20 mm and W =20 mm. The bending test is performed using an ADMET eXpert 7601 equipped with a 10 lbf load cell, with a loading rate set at 1 mm/min.

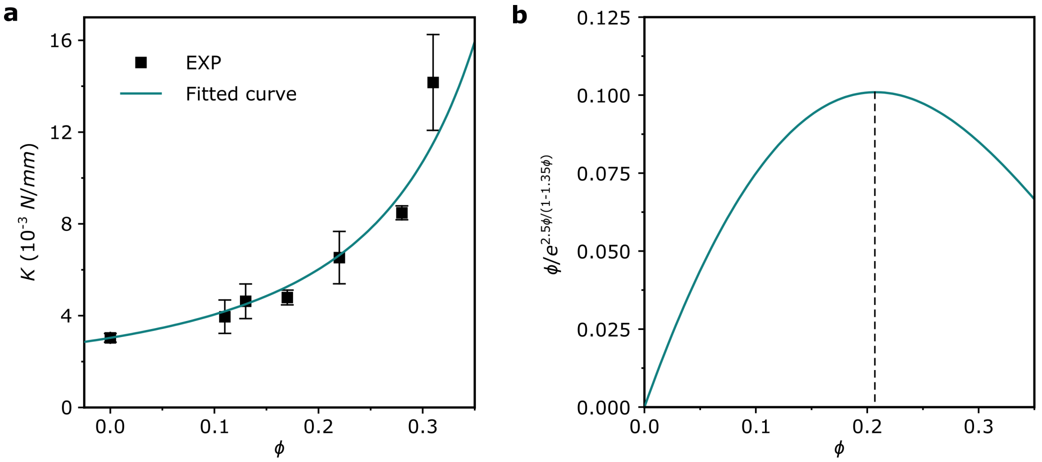

Figure S15. (a) Bending stiffness of the MGO films with different particle weight concentrations. **(b)** Predicted magnetically driven free-end deflection of MGO beams as a function of particle weight concentration.

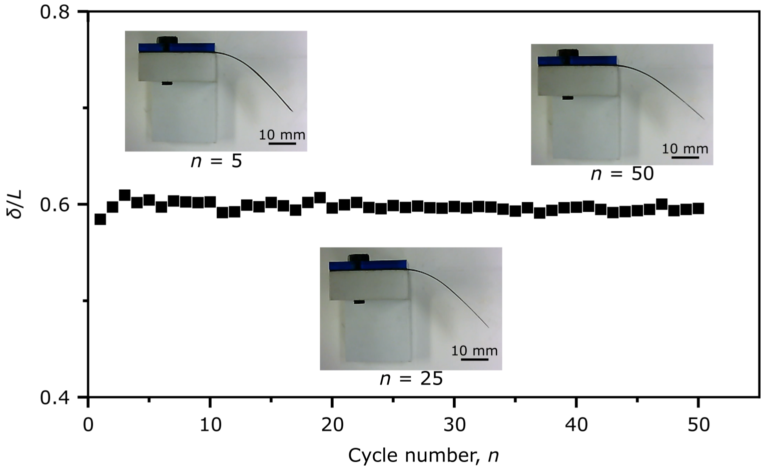

Figure S16. Cyclic stability of the magnetically driven deflection of an MGO6 actuator over 50 loading cycles at $B_{applied}$ = 70 mT. The normalized deflection remains stable, confirming reliable magnetic performance.

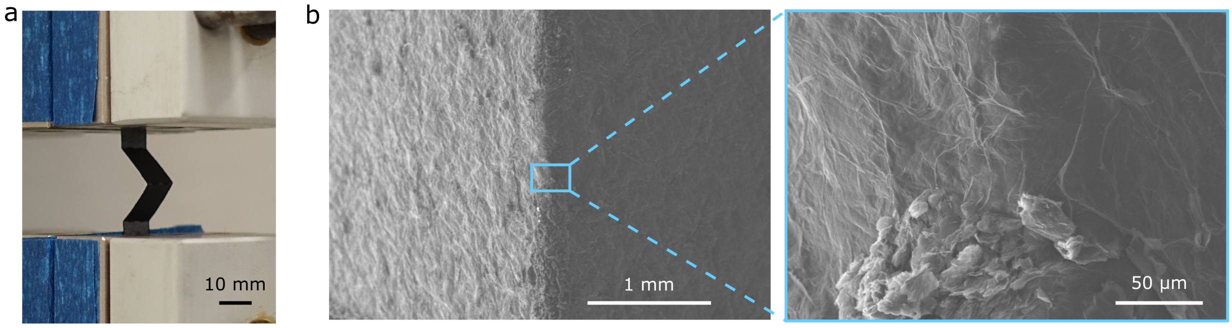

Figure S17. (a) Experimental setup for the cyclic folding/unfolding. (b) SEM image of the crease region after 60 bending-release cycles, showing no crack formation and confirming the structural integrity of the GO layer.

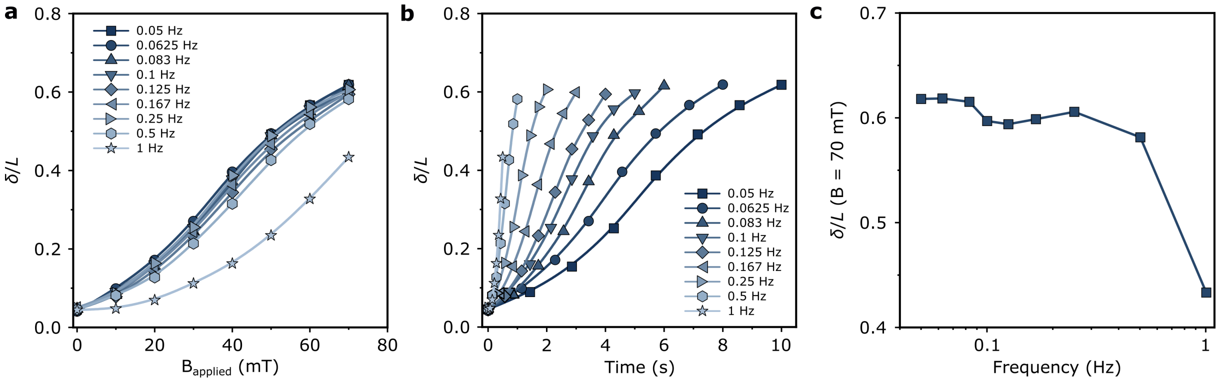

Figure S18. Frequency-dependent actuation performance of MGO6 beams under a uniform magnetic field. (a) Normalized free-end deflection as a function of applied magnetic field at different actuation frequencies. (b) Time-dependent deflection profiles for different frequencies. (c) Comparison of normalized deflection at Bapplied = 70 mT as a function of frequency.

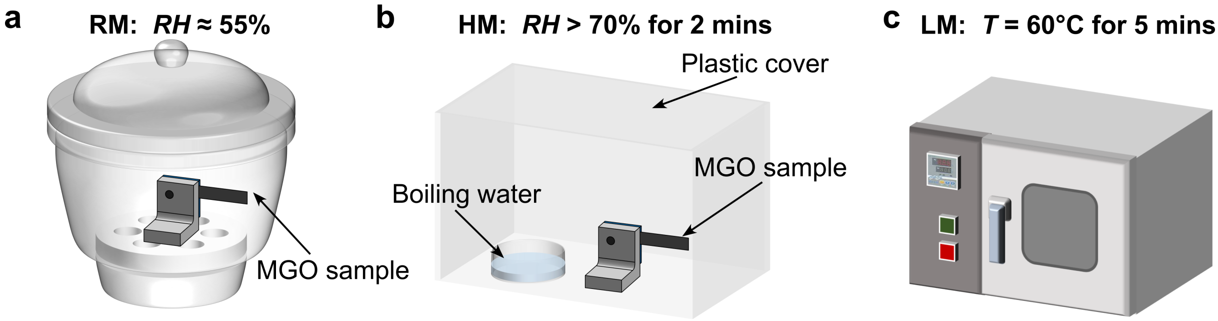

Figure S19. Experimental setup to prepare MGO samples preconditioned at **(a)** room moisture (RM), **(b)** high moisture (HM), and **(c)** low moisture (LM) conditions. After humidifying or dehydrating the MGO samples, magnetic actuation tests on the MGO beams are performed immediately at room humidity (RH ≈ 30%) and room temperature (~23 °C).

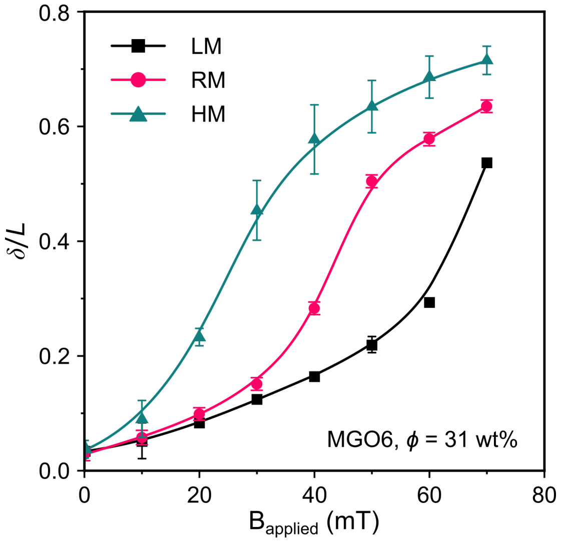

Figure S20. Normalized free-end deflection of MGO6 (ϕ = 31 wt%) under different moisture conditions and magnetic field magnitudes**.**

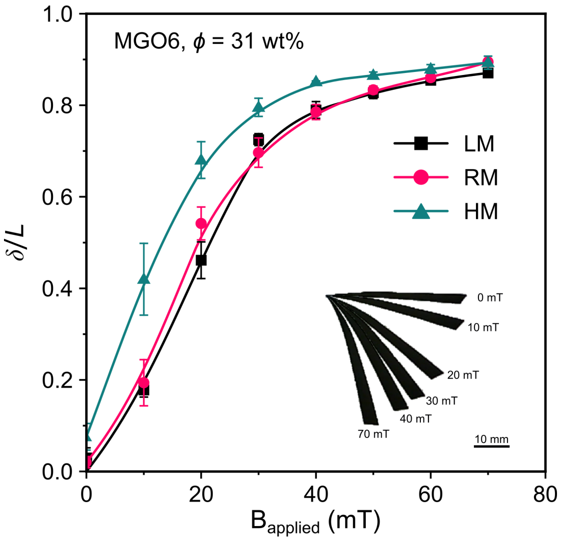

Figure S21. Actuation performance of creased MGO6 (ϕ = 31 wt%) under various moisture conditions and magnetic field magnitudes. Digital picture shows the bending of the creased MGO6 under magnetic actuation at RM conditions.

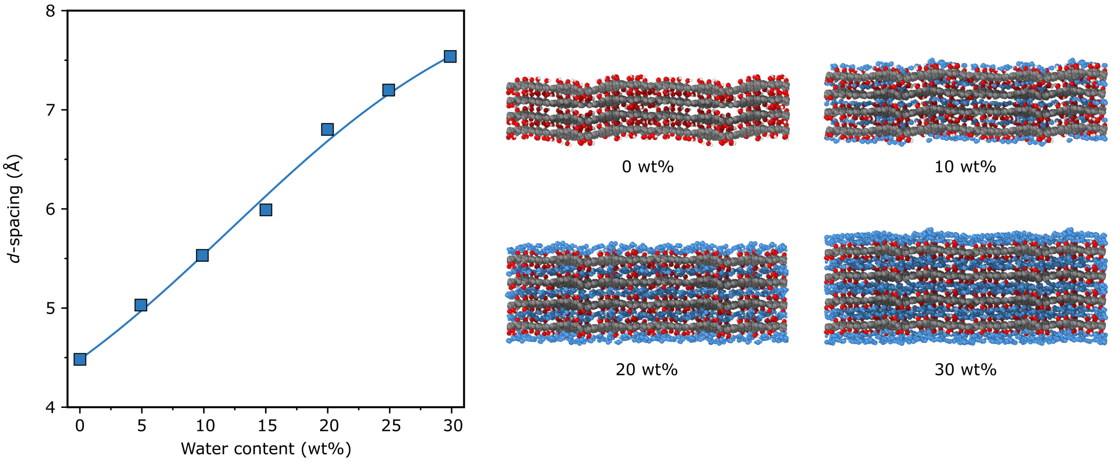

Figure S22. MD simulation results correlating *d*-spacing as a function of water content. The GO structures calculated at 0, 10, 20, and 30 wt% water obtained through MD simulation. Grey, red, and white atoms represent the carbon, oxygen, and hydrogen atoms from GO. The water molecules are colored in blue.

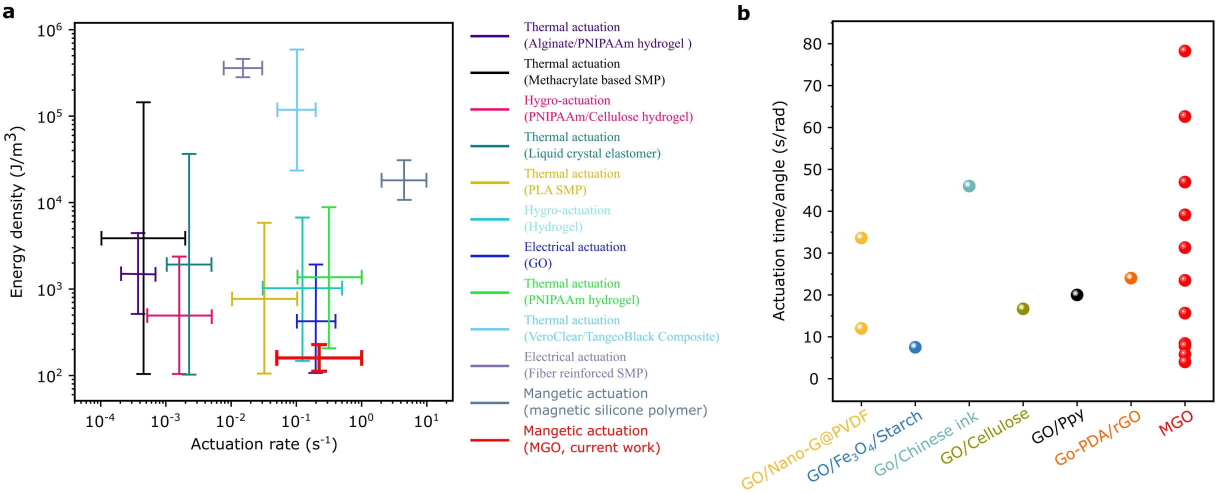

Figure S23. Comparative performance of MGO actuators and conventional actuators. **(a)** Energy density versus actuation rate for MGO actuators compared with representative conventional actuators [11, 80-91]. Actuation density is defined as $f = 1/t$, where t is the time for one completed actuation cycle. For MGO beams (Figure 2a in the manuscript), t represents the time for bending from flat to maximum deflection and then returning to the flat state. For actuators with irreversible deformation, such as shape memory polymers (SMPs) , t is taken as the time for completing the first deformation cycle [11]. Energy density calculations follow Ref. [11]. PNIPAAm = poly(N-isopropylacrylamide); PLA = poly(lactic acid). **(b)** Normalized actuation time of GO-based actuators, comparing humidity-driven, light-driven, and magnetic (MGO) actuation [41, 92-96]. The actuation time is normalized by the angle of the GO-based beam under the actuation.

S6. Pattern Geometry and Bistability of Kresling

A unit cell of the Kresling origami, also known as poly-twist origami [97], comprises an n-sided (n = 6 in this study) base prism, which is tessellated with triangulated identical panels characterized by lengths a and b at an angle α (Fig. S24). In this study, α = 102.77°, a = 13 mm, and b = 19.08 mm. This geometrical configuration ensures the bistability of the fabricated MGO Kresling origami [98]. To produce an MGO Kresling origami, we deviate slightly from the traditional Kresling pattern as we incorporate cuts to replace the "mountain" creases. The cuts are introduced to reduce panel stiffness, allowing the MGO Kresling origami to deform easily under magnetic actuation.

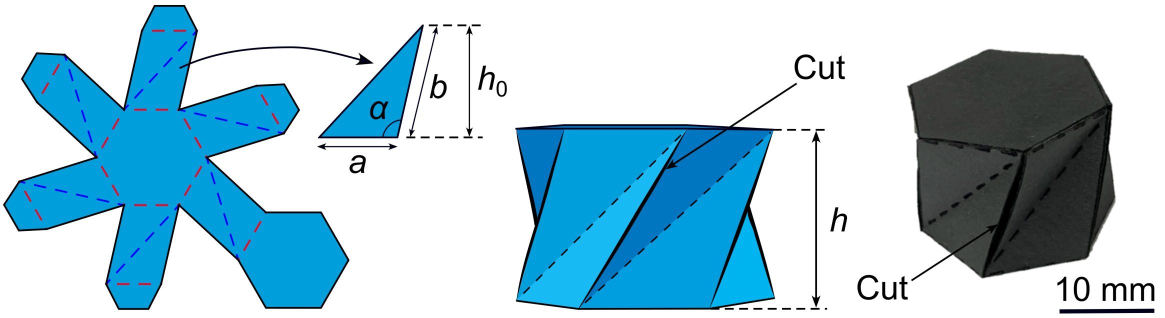

Figure S24. Modified Kresling pattern and the resulting MGO Kresling origami. The blue and red lines represent "valley" and "mountain" creases.

S7. TMP Pattern Geometry and Kinematics

A. Pattern Geometry. The Tachi-Miura polyhedron (TMP) is a bellows-like 3D origami structure based on Miura-ori cells [99-103]. The TMP unit cell is comprised of two sheets, a top layer and a bottom layer as depicted in Fig. S25a. Each sheet is constructed with two symmetric layers and characterized by length parameters l, m, and d and angle α. The top and bottom layers are folded along crease lines (red lines representing mountain creases and blue lines identifying valley creases in Figure S25a). The two folded layers are then bonded together to create a TMP unit cell. Fig. S25b shows a schematic of TMP origami fabrication. Fig. S25d shows the TMP origami fabricated with MGO films, characterized by $m = l = 15$ mm, $d = 10.5$ mm, $\alpha = 70^{\circ}$, and $\theta = 120^{\circ}$. The two folded MGO layers are bonded together using the double-sided tape.

B. Kinematics. Since the TMP origami has a single degree of freedom dictated by the four design parameters (l, m, d, and α), the geometrical characteristics of TMP, denoted as L, W and H, can be expressed as functions of the folding angle θ, defined as the angle between the horizontal and inclined creases (Fig. S25c) [99]:

$$L = 2l + \frac{d}{\tan\alpha} + 2m\cos\theta \qquad \text{(Eq. S14)}$$

$$W = 2d\frac{\sqrt{\tan^2\alpha - \tan^2(\theta/2)}}{\tan\alpha} \qquad \text{(Eq. S15)}$$

$$H = 2m\sin\theta + d\frac{\tan(\theta/2)}{\tan\alpha} \qquad \text{(Eq. S16)}$$

It is crucial to note that $\theta \in [0^{\circ}, 2\alpha]$ and the range of α should satisfy $2l - d\cot\alpha + 2m\cos 2\alpha > 0$ [102]. When $\theta = 0^{\circ}$, the TMP unit cell is folded into a flat state, while at $\theta = 2\alpha$, the TMP unit cell is in a fully folded state ($W = 0$).

In this study, two TMP unit cells are considered, each characterized by different folding angles (TMP-1: $m = l = 15$ mm, $d = 10.5$ mm, $\alpha = 70^{\circ}$, and $\theta = 120^{\circ}$; TMP-2: $m = l = 15$ mm, $d = 10.5$ mm, $\alpha = 70^{\circ}$, and $\theta = 85^{\circ}$, see Fig. S26). To assess the loading capacities of the designed TMP samples, we analyze the height (H) of TMP unit cells at different folding angles (the height H is normalized by the crease length m, as illustrate in Fig. S26a). Assuming rigid origami, where deformation occurs only in the creases while the panels remain rigid without deformation, it is found that the height initially increases with the folding angle, reaching a maximum height at a critical folding angle, and then decreases with further folding. If the folding angle is smaller than the critical folding angle at the initial state (e.g., TMP-2), the structure collapses with an applied compressive load along the z-direction. However, if the initial state of the TMP is beyond the critical folding angle (e.g., TMP-1), the height of the structure is maintained kinematically, preventing it from returning to the flat state, indicating a load-bearing capability.

TMP origami demonstrates mechanical bifurcation between collapsible and load-bearing configurations, determined by the initial posture of the TMP [99]. It is important to note that not all TMP unit cells exhibit bifurcated folding motion, dependent on the geometrical parameters of patterns. Based on Fig. S26a, for TMP unit cell with bifurcated folding motion, a critical transition from a

collapsible configuration to a load-bearing configuration can be found, corresponding to the maximum height of the TMP unit cell. Therefore, the existence of the critical folding angle (θ_c) is examined by solving $dH/d\theta = 0$, leading to

$$\theta_c = \arccos\left(\frac{1}{2}\left(\sqrt{1-2\frac{d}{m}\frac{1}{\tan\alpha}}-1\right)\right) \quad \text{(Eq. S17)}$$

Based on Equation (S10), the critical folding angle (θ_c) is found dependent on the geometrical parameters of the patterns, specifically d/m and α. Given the geometrical parameters, if the obtained critical folding angle (θ_c) falls within a reasonable range (i.e., $[0°, 2\alpha]$), the TMP unit cell exhibits the mechanical bifurcation between collapsible and load-bearing configurations. Conversely, if the critical folding angle (θ_c) falls outside this range, the TMP unit cell only demonstrate collapsible capability.

In Fig. 26b, we present a contour plot illustrating the evolution of height (H) normalized by the creased length (m) as a function of the folding angle (θ) and angel between crease (α) for a TMP unit cell characterized by d/m = 0.7. The red curve in the contour plot (Fig. S26b) is driven from Equation (S10) and represents the critical folding angle (θ_c), while the black line demotes another critical folding angle where the TMP unit cell is fully folded based on $\theta = 2\alpha$. Our results highlight that, when d/m = 0.7, the geometrical parameter (i.e., angel between creases (α)) of the TMP pattern significantly affects the mechanical responses, which are further influenced by the folding angle of the TMP. In Fig. S26b, an intersection between the red and black lines is observed, representing the critical angle ($\alpha_c \approx 56.26^\circ$) for pattern design. When the angle between creases (α) is smaller than α_c, the TMP will exclusively exhibit a collapsible mode, regardless of the initial posture setting. While the angle between creases (α) exceeds α_c, the TMP can display both collapsible and load-bearing behaviors, contingent upon its folding angle (θ). Notably, from Fig. S26b, it is evident that TMP-1 possesses load-bearing capability, while TMP-2 is collapsible without load-bearing capacity.

To validate our theoretical analysis of the mechanical response of TMP origami, we conduct a compressive testing on the TMP samples (TMP-1 and TMP-2 as shown in Fig. S26c). The stress-strain responses (Fig. S26c) indicate that TMP-1 enables loading-bearing capacity, and TMP-2 displays collapsible behavior under compression. These experimental findings align with the mechanical behaviors predicted through our theoretical analysis.

In addition to the angle between creases (α), the mechanical behaviors of TMP can also be tuned by varying the ratio between crease lengths, denoted as d/m. In Figs. S26d-S26f, we report as contour plots the normalized height (h/m) as a function of the ratio between crease lengths (d/m) and the folding angle (θ) for TMPs characterized by different angles between creases (α = 45°, 60°, and 70°). The results indicate that, when α = 45° and $0.2 \le d/m \le 1.2$, the TMP exclusively exhibits collapsible folding motion, irrespective of the folding angle (θ). When α = 60° and 70°, the TMP can demonstrate both collapsible and loadbearing behaviors by selecting an appropriate ratio between crease lengths (d/m).

C. Magnetically-actuated state change. Fig. S27 shows the magnetization pattern of the MGO-TMP origami, which is magnetized in its fully folded state with the magnetic field aligned parallel to the panels. In Fig. 3g of the main text, the shape morphing of MGO-TMP is shown, transitioning from a collapsible to a load-bearing state under actuation by a permanent magnet. Fig. S27a shows the transition of MGO-TMP from a collapsible to a load-bearing configuration under a uniform magnetic field in water. Fig. S27b illustrates the height (H) of the MGO TMP normalized by the crease length (m = 15 mm), plotted as a function of the applied magnetic field magnitude.

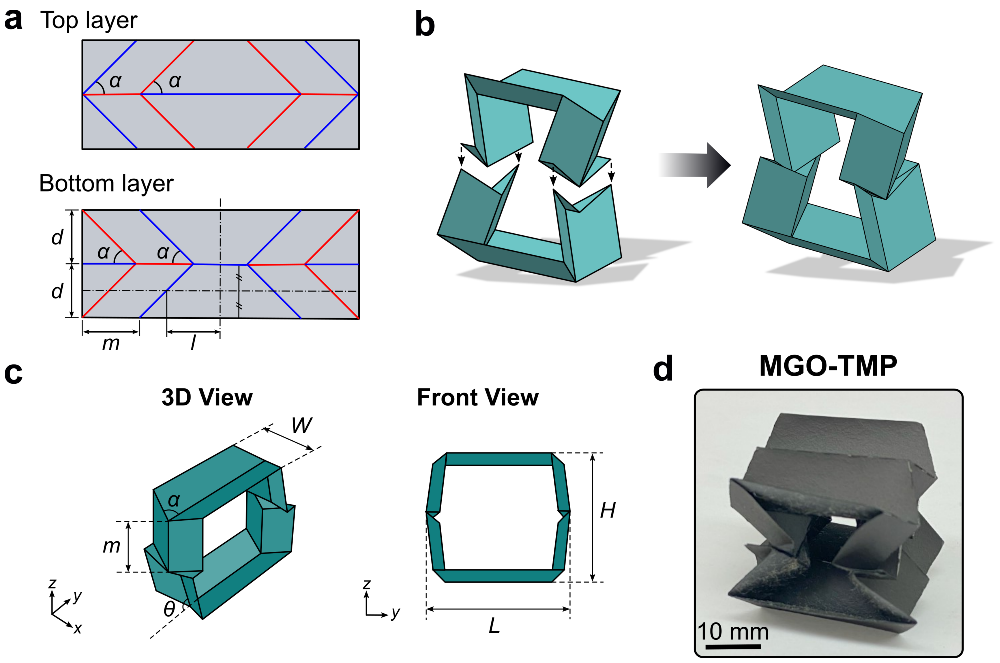

Figure S25. (a) Tachi-Miura Polyhedron (TMP) patterns. Red and blue creases represent "mountain" and "valley" creases, respectively. **(b)** Fabrication process of a TMP origami. **(c)** A TMP origami unit cell in the side view and front view. **(d)** A TMP origami made of MGO film.

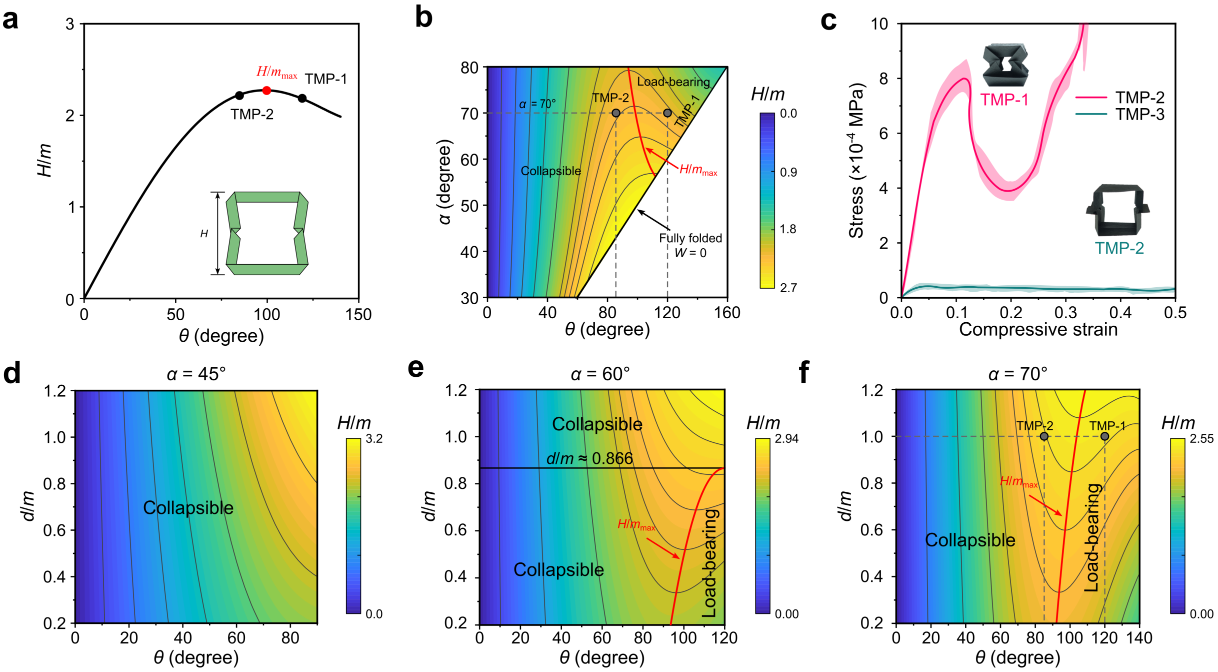

Figure S26. Kinematic analysis of the TMP unit cell. **(a)** The height of the TMP unit cell as a function of the folding angle. **(b)** Contour plot of the height of the TMP unit cell as a function of angle α and folding angle θ. The red curved line represents the maximum height of TMP with given geometrical parameters during the folding/deploying process. The black line represents the critical folding angle where the TMP unit cell is fully folded. **(c)** Stress-strain curves of MGO TMP origami characterized by different folding angles θ. Contour plot of the height of TMP unit cells characterized by **(d)** $\alpha = 45^{\circ}$, **(e)** $\alpha = 60^{\circ}$, **(f)** $\alpha = 70^{\circ}$ as a function of the crease length ratio (d/m) and folding angle θ.

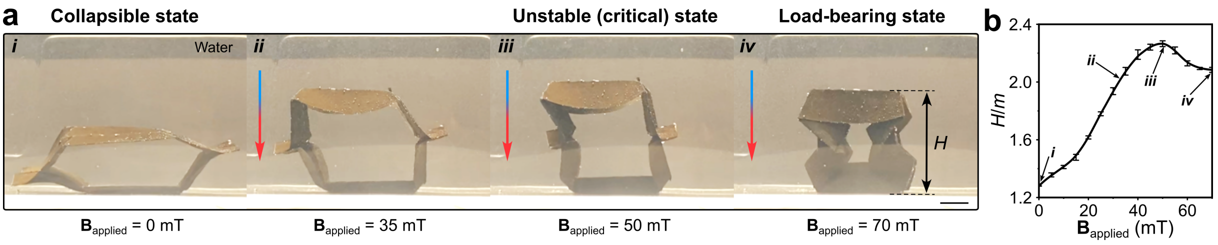

Figure S27. **(a)** Magnetically-actuated state change of MGO-TMP structure in water under a uniform magnetic field generated by a Helmholtz coil. Scale bar: 10 mm. **(b)** Normalized height (H/m) of MGO-TMP as a function of applied magnetic field magnitude. Here m = 15 mm represents the crease length, as shown in Fig. S25c.

S8. MGO Soft Robots

A. Inchworm-inspired soft robot. The inchworm's distinctive mode of locomotion involves two primary steps: (*i*) gripping the substrate with the true legs while the prolegs advance via pulling forces from abdominal contractions, actuated by longitudinal muscle fibers; (*ii*) forming an "omega" shape with its body, followed by the prolegs gripping the substrate as the front part of its body extends forward (Fig. 5a) [104]. Fig. S32 shows a schematic of the inchworm-inspired MGO/GO soft robot with geometrical dimensions and magnetization patterns. The body of the soft robot is made by GO film with a mountain crease (dashed red line in Fig. S32). Two MGO films with in-plane magnetization are used as the longitudinal muscle fibers of the inchworm to induce bending of the soft robot by magnetic actuation. The mountain crease, introduced by laser cutting, facilitates easier bending of the soft robot's body.

The difference in the feet design results in asymmetric friction during the actuated and released states. As shown in Fig. S33, in the *actuated state*, when a magnetic field is applied, the body bends upward, and the front foot changes its contact with the ground from edge contact to surface contact. While in Coulomb friction model for rigid and dry surface, friction force is independent of the contact area. This increases the effective real contact area is known experimentally to increase the actual frictional force due to more asperities and adhesive interactions [25]. As a result, the front foot develops higher friction and remains nearly stationary, while the back foot moves forward (ΔL_A(BF)) as the body contracts. In the *release state*, when the magnetic field decreases, the robot returns to its flat configuration under gravity. At the beginning of this recovery process, the back foot has a larger contact angle (θ(BF)) than the front foot (θ(FF)), which gives it a higher static friction (Fig. S33(*iv*)). Consequently, the front foot begins sliding forward first. Since dynamic friction is much smaller than static friction, the front foot continues to slide forward (ΔL_R(FF)), resulting in net locomotion. Occasionally, small backward motion of the back foot is observed, but overall, the asymmetry ensures forward displacement of the robot. At the same time, as the contact angle of the front foot (θ(FF)) decreases, the GO surface briefly transitions to surface contact with the ground (middle figure in the released state, Fig. S33). This contact occurs only for a very short duration before reverting back to edge contact, as shown in the final stage of the released state.

B. Jellyfish-inspired soft robot. Ephyra (juvenile scyphomedusae [105]) exhibit distinctive morphological features that differ significantly from their adult counterparts. Unlike the circular disks of adult medusae, ephyra possess bells with deep clefts between lappets (Fig. 5f), which are instrumental in efficient aquatic locomotion by controlling fluid flow. Inspired by these ephyra, we propose a jellyfish-inspired soft robot fabricated from MGO films. Different from the previous fabrication method of jellyfish-like soft robot that replies on multi-step assembly [106], our approach using MGO films enables rapid construction by simply cutting the MGO films into ephyra-like pattern (Fig. 5f). Fig. S34 illustrates the dimensions of the jellyfish-inspired soft robot. Fig. S35 details the magnetization of the jellyfish-inspired soft robots for different movements in water. For upward swimming (as shown in Fig.

5h of the main text), the soft robot is magnetized with a strong magnetic field (> 3T) along the negative *z*-direction when the soft robot is in a contracted state, where all eight lappets are bent downward in a rotationally symmetric configuration (Fig. S35a). For walking from the left side to the right side in water, two of the lappets are bent more than the other six lappets during magnetization (Fig. S35b). After this, the soft robot's magnetization pattern is no longer rotationally symmetric. As a result, under the same magnetic field, the lappets that are bent more during magnetization show more pronounced bending, with their ends touching the bottom of the container (e.g., $t = 6.5$s in Fig. 5i). This results in asymmetric water flow, causing the soft robot to move from the left side to the right side of the container. Fig. S36 shows the magnetic field applied to actuate the jellyfish-inspired soft robot for walking from the left to the right side of the container.

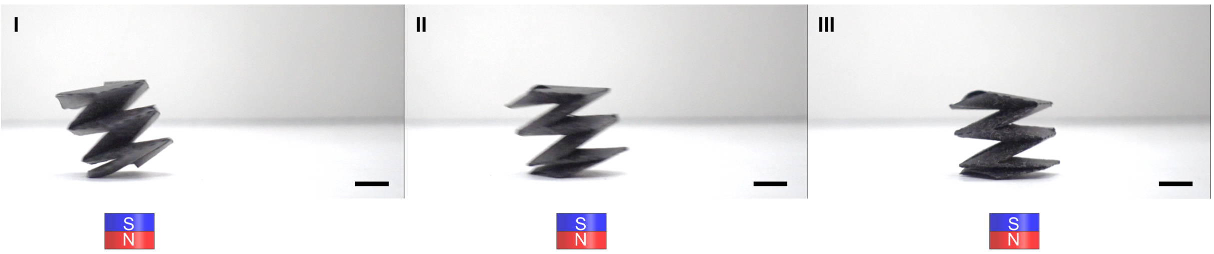

Figure S28. Horizontal movement of the permanent magnet enables straight walking of MGO Miura-ori tube soft robot. Scale bar: 10 mm.

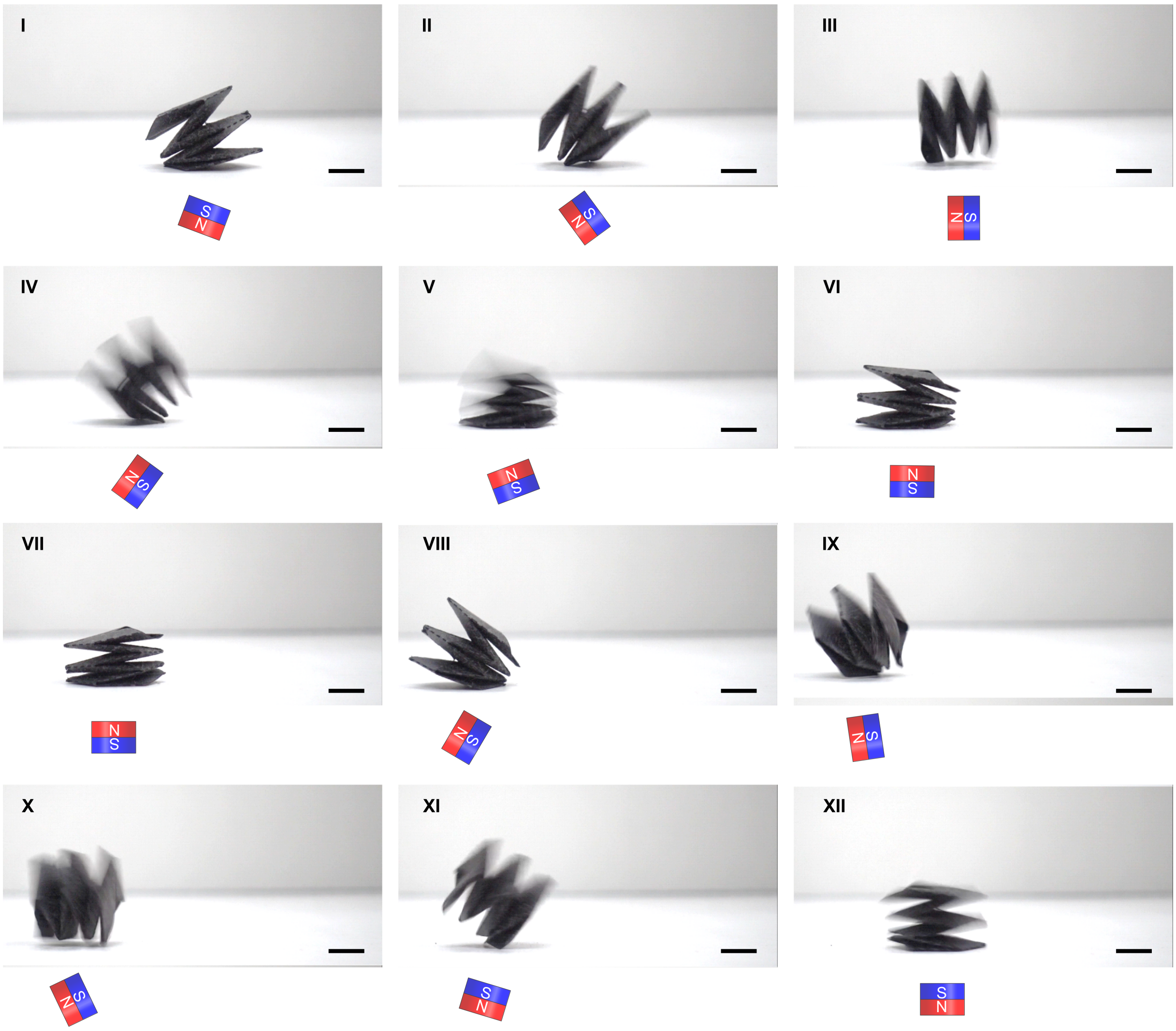

Figure S29. Rotational movement of a permanent magnet makes the MGO Miura-ori tube soft robot flip. The Miura-ori tube soft robot can roll clockwise and counterclockwise by rotating the permanent magnet counterclockwise and clockwise, respectively. Scale bar: 10 mm.

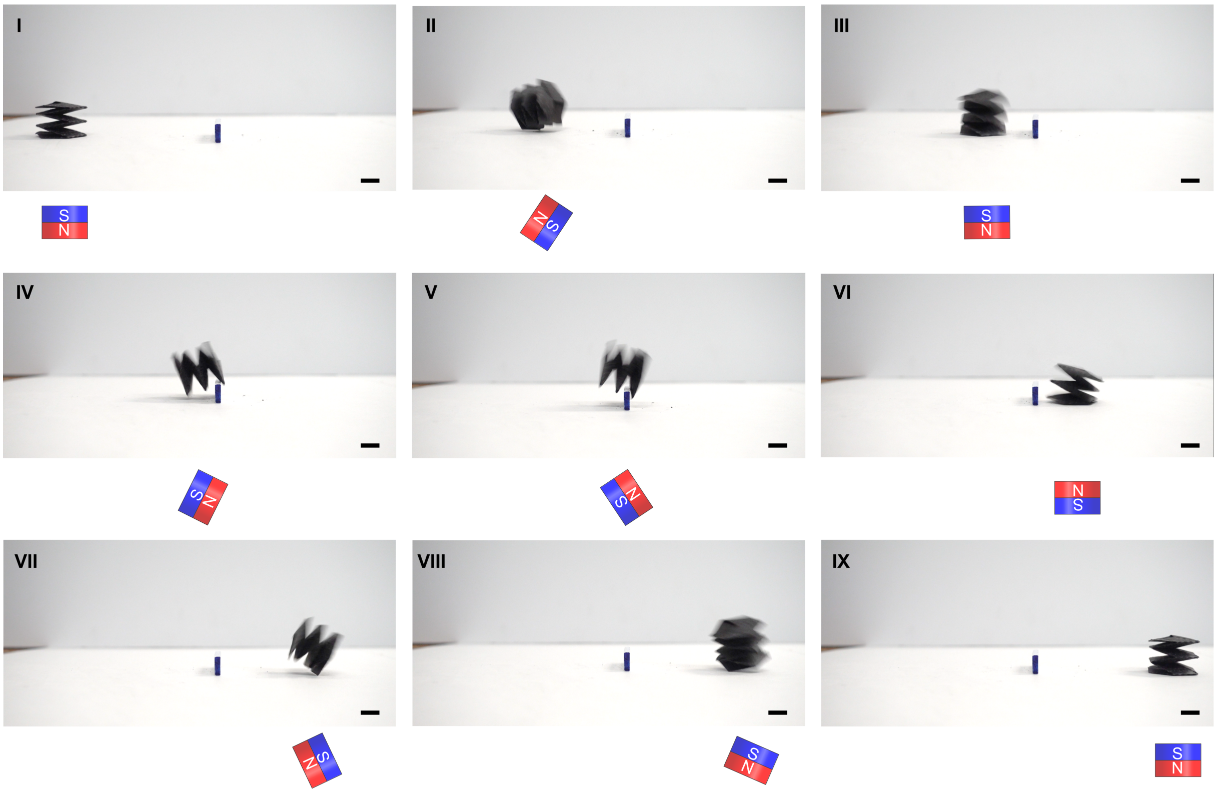

Figure S30. With combined rotational and horizontal movements of a permanent magnet beneath the soft robot, the MGO Miura-ori tube soft robot climbs over a wall. Scale bar: 10 mm.

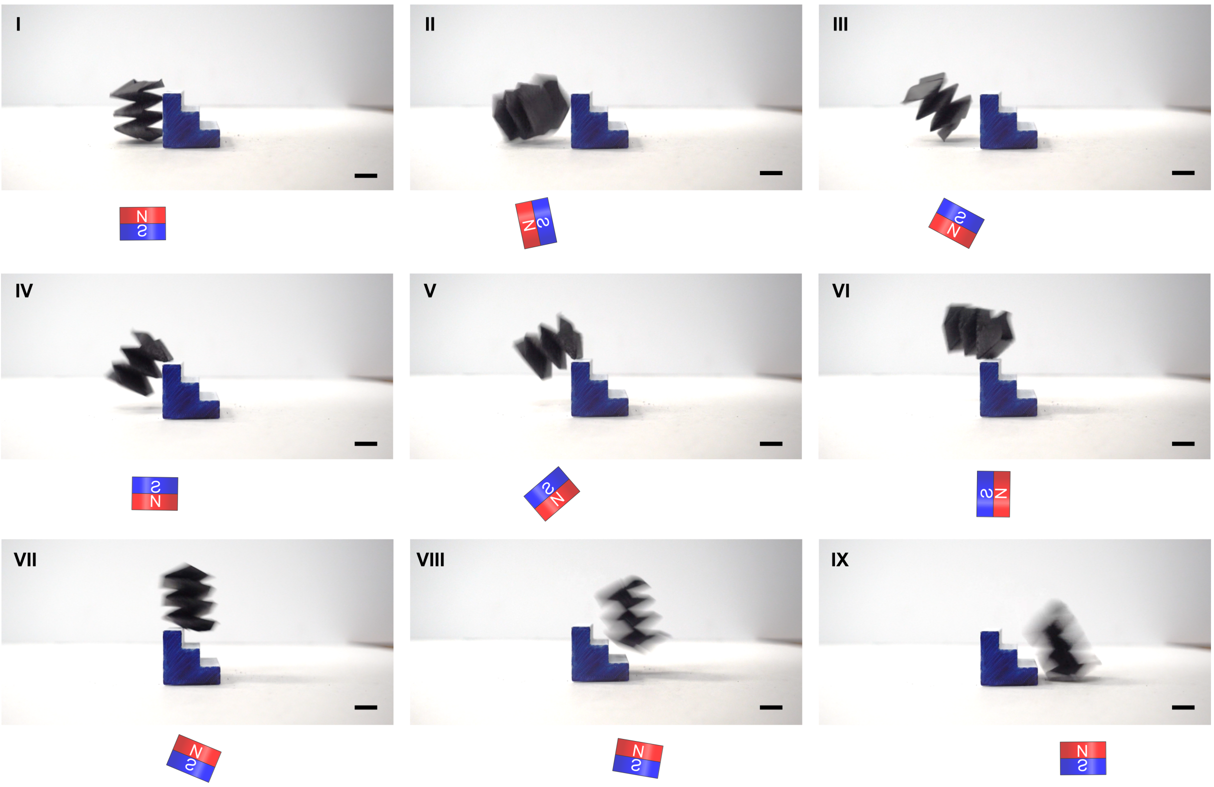

Figure S31. With combined rotational and horizontal movements of a permanent magnet, the Miura-ori tube soft robot can overcome stairs. Scale bar: 10 mm.

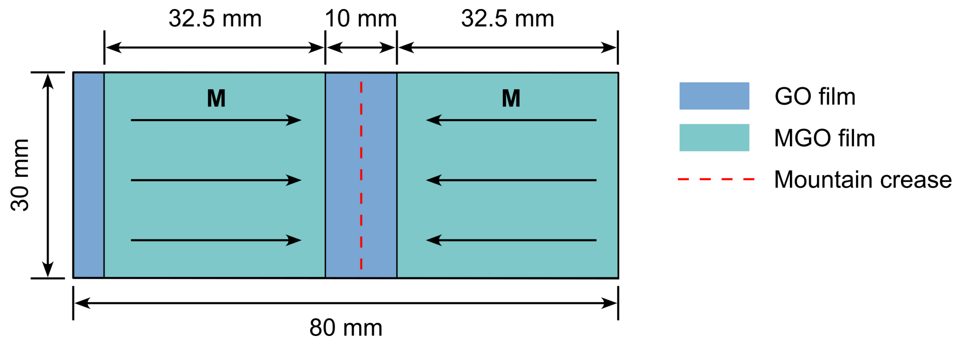

Figure S32. Geometrical dimensions of the inchworm-inspired MGO/GO soft robot.

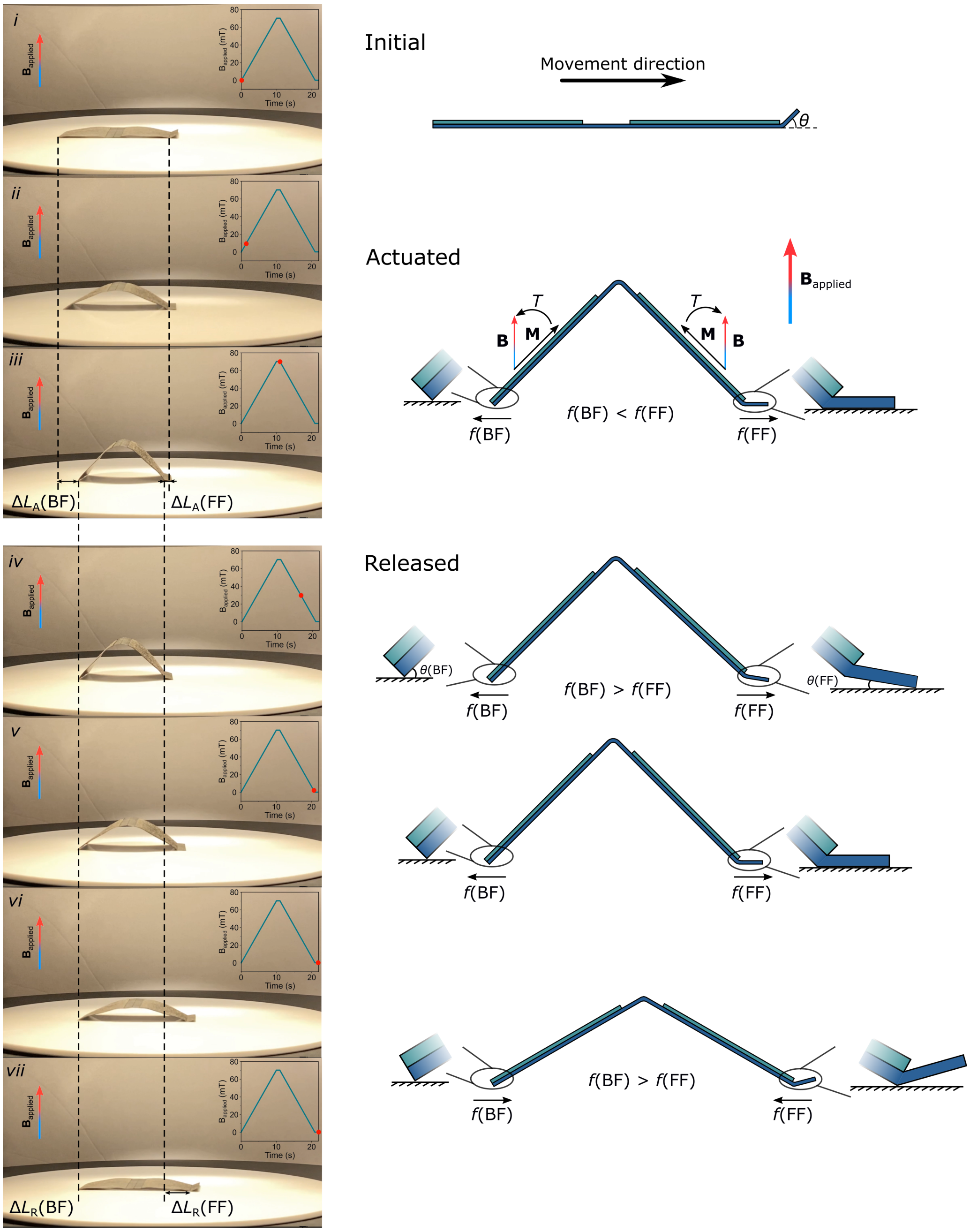

Figure S33. Magnetically-driven locomotion of the MGO/GO soft robot. Schematics illustrating the locomotion mechanism of the inchworm-inspired soft robot.

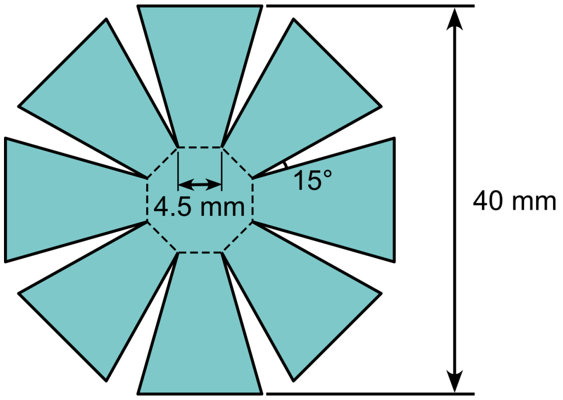

Figure S34. Geometrical dimensions of the jellyfish-inspired MGO soft robot.

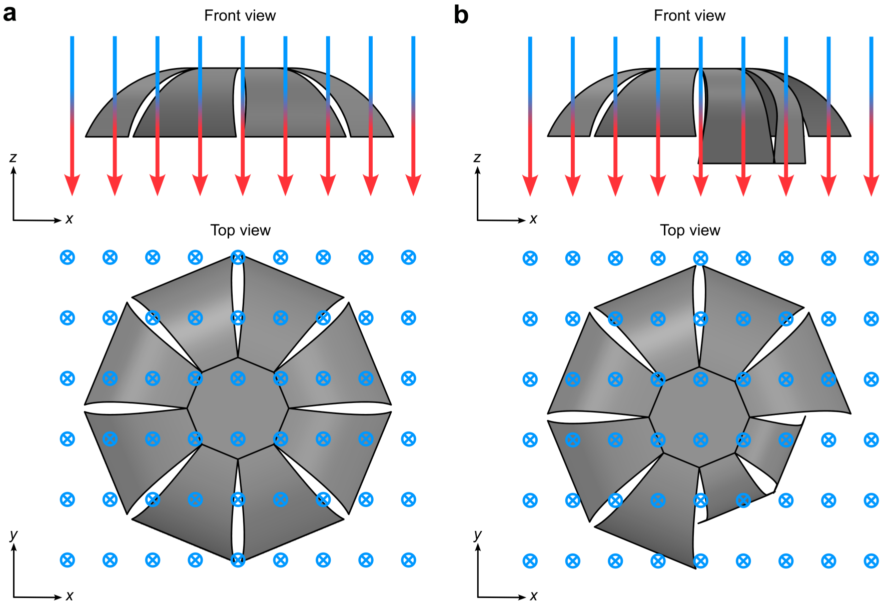

Figure S35. Magnetization of Jellyfish-inspired MGO soft robots for **(a)** upward swimming and **(b)** swimming from the left to the right side of the container.

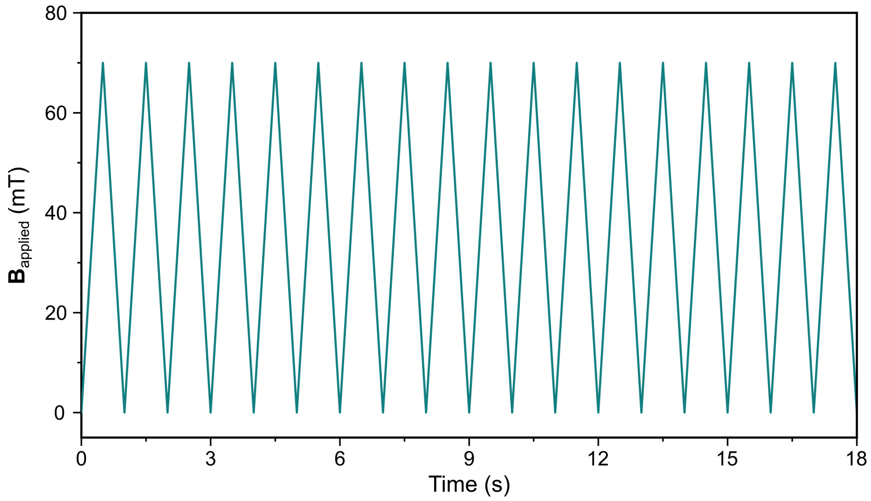

Figure S36. Magnetic field applied for actuating the jellyfish-inspired soft robot to swim from the left to the right side of the container.

S9. Programming and Reprogramming of Magnetization Patterns

There are a lot of conventional magnetization reprogramming strategies reported in the literature [18, 26, 30, 35, 40, 107-109]. ***Thermal demagnetization and remagnetization***. A common strategy is to heat the material above its Curie temperature (globally or locally with lasers), then remagnetize it during cooling [30, 33]. This method is generally limited to magnetic materials with relatively low Curie temperatures (e.g., CrO_2), which are incompatible with high-remanence magnetic materials such as NdFeB or SmCo [26]. Moreover, low-Curie-temperature particles typically exhibit lower remanence and coercivity, CrO_2 microparticles, for example, have a remanence of ~75 kAm^{-1} (8× lower than NdFeB particles with similar size) and a coercivity of ~50 mT (12× lower than NdFeB particles with similar size), leading to a maximum torque density nearly two orders of magnitude smaller [65]. This method is also highly energy-consuming, and for large-scale structures, localized heating is impractical when target regions are hidden within the structure. Additionally, magnetizer chambers often have limited size (e.g., ~60 mm diameter × 100 mm length in our setup), making remagnetization of large structures impossible. ***Particle reorientation during phase transition***. Another strategy involves physically rotating magnetic particles in a polymer matrix under an applied field during a solid-to-liquid transition, or by cleavage of dynamic linkages at elevated temperatures [107]. While this method can use high-remanence particles such as NdFeB, it still requires elevated temperatures (high energy input), local heating in some complex 3D structures is challenging, and the inclusion of the meltable solid phase (i.e., the phase-changing beads encasing the magnetic particles) increases in the average size of particulate fillers, may affect the mechanical properties and behavior of the composite [65, 110, 111]. ***Assembly/disassembly of modular magnetic units***. A third approach assembles multiple magnetic modules via magnetic attraction or electrostatic anchoring . While this allows reconfigurability, it suffers from mechanical instability, as the modules can shift under external perturbations [26, 112]. The modular interfaces may also weaken structural robustness, and achieving precise, repeatable alignment in complex or dynamic environments can be challenging.

In contrast, our strategy uses a thin GO substrate onto which detachable, pre-magnetized MGO stickers can be easily attached or rearranged. This approach has several advantages: (*i*) No need for global re-magnetization: Pre-magnetized stickers with defined magnetization directions are directly applied, avoiding energy-intensive heating or re-magnetization. (*ii*) Scalability to large structures: Even if a structure exceeds the physical size of a magnetizer chamber, multiple smaller stickers can be attached across the surface to achieve the desired global magnetization pattern. (*iii*) Programmable heterogeneity: magnetic stickers with different magnetization orientations or magnetizations (modified by particle concentrations) can be combined to achieve spatially varying actuation responses. (*iv*) Origami-enabled accessibility: Complex structures can be unfolded, allowing stickers to be detached and reattached more easily. Alternatively, new origami structures can be fabricated and reprogrammed by reusing existing stickers. (*v*) Reconfigurability with durability: Our adhesion and cycling tests show that the magnetic stickers can be attached/detached repeatedly with stable adhesion and magnetization retention. We

performed lap-shear tests on samples bonded with different adhesives, including poly(acrylic acid) (PAA), poly(ethyleneimine) (PEI), and borax (Fig. S37). The glued joint area was controlled at 4 mm × 2 mm to ensure consistent contact. After air-drying for 2 hours and pressing under a plastic panel for 24 hours, the bonded specimens were tested in tension using an ADMET eXpert 7601 machine equipped with a 10 lbf load cell, at a loading rate of 0.01 mm/s. The results show that PAA provides the highest adhesion force (~3.5 N), outperforming PEI and borax (~2-2.5 N), while still enabling detachability (the ultimate tensile force for pristine GO is ~6 N). We also tested GO solution and water as potential "glues", but they failed to form effective bonds between GO films and were easily detached. To evaluate the mechanical stability of PAA adhesion, dogbone samples bonded with PAA were subjected to cyclic tensile load-unload testing (20 cycles, 1.5 mm displacement). The stable force-displacement responses shown in Fig. S38 confirm the mechanical durability of the adhesive interface. These results confirm that PAA provides an optimal balance between reliable bonding and reusability for detachable MGO systems.

Fig. S39 illustrates the principle of detachable MGO-sticker-assisted programming and reprogramming of magnetization patterns. In general, our reprogramming approach offers two key functions: (*i*) generating different deformation modes under the same magnetic field (Fig. 6b in the manuscript), and (*ii*) tuning the magnetic field strength required to reach a target deformation (Figs. 6c-6g in the manuscript). In Fig. S39a-b, we demonstrate the first function using a four-armed GO substrate with detachable MGO stickers. Each arm is defined by a discrete state: "+1" for upward deformation, "-1" for downward deformation, and "0" for no deformation. Based on this coding, the system can realize up to $3^4 = 81$ possible configurations. As an example, we show the 27 configurations achievable when arm 1 is fixed in the "+1" state. Reprogramming between configurations is straightforward: simply detaching and reattaching stickers redefines the magnetization pattern. Moreover, achieving the "0" state (no deformation) can be done by removing the sticker, something not feasible in conventional reprogramming methods. For example, in the case of particle reorientation during a solid–liquid phase transition (strategy *ii*), achieving a "no magnetization" state is particularly challenging: the magnetic particles would need to remain randomly distributed after resolidification, with each particle retaining a random magnetization direction, which is difficult to ensure in practice. While modular assembly strategies require disassembly and reassembly of entire units. Also, in our approach, all stickers are reusable, and only a small amount of material is needed to realize a wide range of configurations. This modularity suggests potential applications in mechanical computing or logic gate operations, where distinct magnetization patterns correspond to defined logical outputs. Furthermore, in addition to programming along the in-plane magnetization direction shown in Fig. S36a, stickers can be magnetized in other orientations (Fig. S36c), enabling even richer deformation modes and shape transformations.

In Fig. S36d, we highlight the second function: tuning the magnetic field threshold for actuation. By fabricating stickers with different magnetic particle concentrations, their remanent magnetization can be systematically varied. As an example, for a four-armed structure, the stickers attached to arms 1-4 were

prepared with magnetizations of 4M, 3M, 2M, and M, respectively. The free-end deflection curves show distinct deflection-magnetic field relationships, with arm 1 bending most easily and arm 4 requiring the strongest field. To achieve the same target deflection of 9 mm, the required magnetic fields were 35, 47, 70, and 140 mT for arms 1, 2, 3, and 4, respectively. As the external magnetic field ramps linearly, the arms deform and reach the target displacement sequentially. This tunable, sequential actuation highlights an important function of our approach, with potential applications in timed robotic motion, programmable switches, or sequential release systems.

In summary, compared with conventional reprogramming strategies, our detachable MGO-sticker approach offers a low-energy, scalable, and reusable method to program and reprogram magnetization patterns. It enables not only diverse deformation modes (including the "0" state) but also tunable actuation thresholds, which are difficult to realize with existing approaches. These unique capabilities highlight the novelty and potential of our strategy for sustainable and reconfigurable soft magnetoactive systems.

In this study, we employ MGO stickers with different magnetization to program and reprogram the magnetization patterns. Each MGO sticker consists of a GO layer and a magnetically active layer. In most cases, the GO side is placed in contact with the GO substrate during attachment and detachment (e.g., Fig. 6b), ensuring that the magnetic layer remains unexposed to direct adhesive interaction. However, in certain configurations, such as the Kresling origami structures shown in Figs. 6f and 6g, direct contact between the magnetic layer and the underlying substrate is occasionally unavoidable. In these cases, we carefully detach the MGO stickers and have not observed any visible damage or delamination of the magnetic layer. Based on these experimental observations, we conclude that the magnetization of the MGO stickers remains stable and is not affected by repeated cycles of attachment and detachment, supporting their robustness for reprogrammable actuation applications.

In the experiment shown in Fig. 6a of the main text, we employ a six-armed structure to illustrate the magnetic-sticker-assisted reprogramming of MGO soft machines. Unlike the initial fabrication method shown in Fig. 3a, where the six-armed structure was constructed using MGO film, we used a GO film for the body of the six-armed structure. The GO six-armed structure is first perforated using a laser cutter (Speedy 100, Trotec Laser, CANADA. Laser power: 19 W; cutting speed: 5.95 inch/s) along the prescribed pattern. The MGO strips (40 mm × 10 mm) functioning as stickers are prepared by laser cutting a rectangular pattern (Laser power: 20 W; cutting speed: 3.5 inch/s). Through the attachment, detachment, and arrangement of magnetized MGO stickers, we effectively programmed and reprogrammed the magnetization patterns while reusing and recycling the detached MGO stickers. The integration of a specialized adhesive, namely PAA (10 g/L), promotes the bonding between MGO and GO layers, allowing both strong adhesion and facile detachment of MGO from the GO substrate (see Experimental Section in the main text and Movie S8). The six-armed magnetic structure showed different configurations under an applied magnetic field, consistent with the predictions derived from FEA simulations (Movie S8).

Fig. S41a shows a Kresling origami with an assembled MGO magnet (marked by a red dashed line) glued on the hexagonal panel of the GO Kresling origami using PAA (10 g/L). This MGO/GO Kresling origami system is bistable, with two different states: the deployed state and the folded state. The black hexagonal plate represents the assembled MGO magnet with an in-plane magnetization (**M**). The transition between these bistable states can be triggered by applying a magnetic field, as shown in Fig. S41b. The angle between the magnetization (**M**) and applied magnetic field ($\mathbf{B}_{\mathbf{applied}}$) is represented by $\alpha_{\text{M-B}}$. $\mathbf{B}_{\text{applied}}$ is applied normally to the magnetization direction (i.e., $\alpha_{\text{M-B}} = 90°$). $\Delta\theta$ indicates the rotation angle of the assembled MGO magnet due to magnetic torque. When the induced magnetic torque ($\boldsymbol{T} = V(\mathbf{M} \times \mathbf{B}_{\text{applied}})$, where V is the volume of the assembled MGO magnet) is large enough to overcome the required torque (T_r, $T_r^{'}$) for the transition from a deployed stable state to a folded stable state or vice versa, the MGO/GO Kresling origami transitions from one state to the other. If the magnetic torque falls below the required torque at any angle during the folding/unfolding process, MGO/GO Kresling origami fails to transition to the next stable state and returns to the initial stable state upon removal of the magnetic field. Figs. S41c and S41d show stable states transition of MGO/GO Kresling by a magnetic field. The insets show a schematic of the MGO/GO Kresling origami with the left side fixed to a support and the right side attached to an assembled MGO magnet with in-plane magnetization (**M**), as depicted in the picture in Fig. S41a. The sudden change in length (L) occurring when $\mathbf{B}_{\text{applied}}$ is between 25 and 30 mT represents the transition between the two stable states (Fig. S41c).

Fig. S42 introduces a strategy for programming the mechanologic output, allowing for different outcomes based on the applied magnetic fields. Using three MGO/GO Kresling structures, the logic computing device can achieve all possible global mechanologic outputs by programming the applied magnetic field.

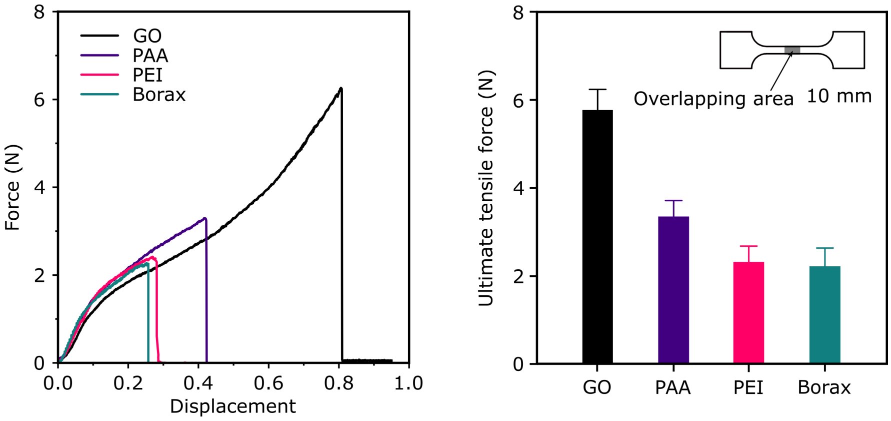

Figure S37. Adhesion performance of detachable MGO stickers using different bonding agents. (Left) Force-displacement curves of lap-shear tests for samples bonded with PAA, PEI, and borax, compared with pristine GO. (Right) Ultimate tensile force extracted from the curves, showing that PAA provides higher bonding strength than PEI and borax, while still enabling detachability.

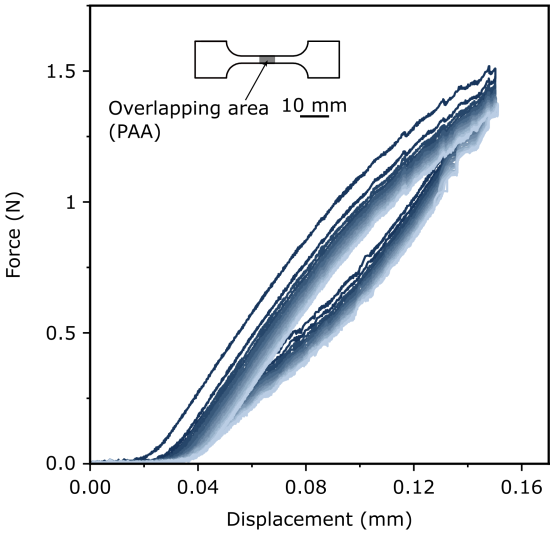

Figure S38. Cyclic (tensile load-unload) responses of dogbone sample bonded by PAA.

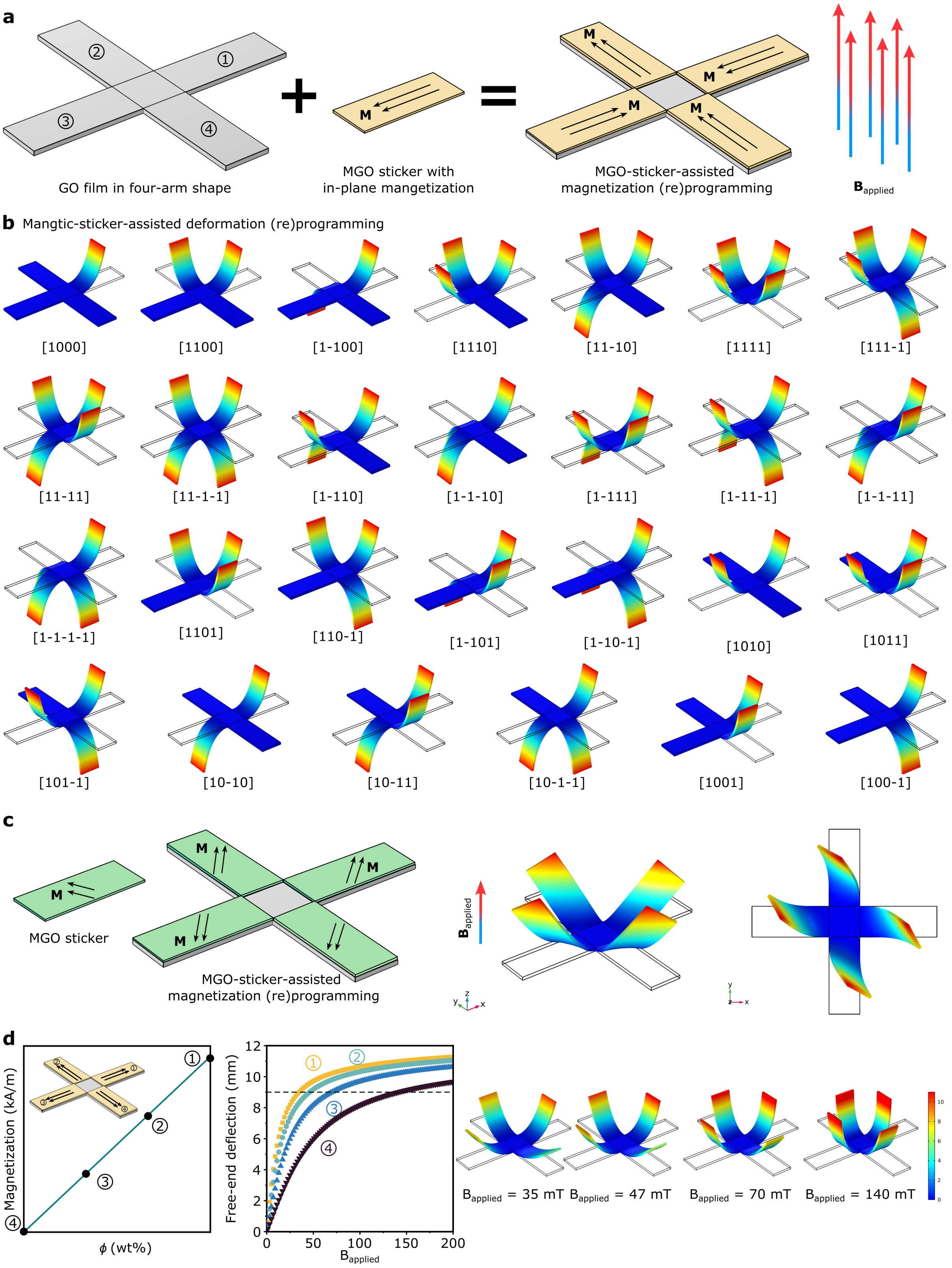

Figure S39. MGO-sticker-assisted magnetization programming and reprogramming. (a) Principle of using magnetic stickers for magnetization (re)programming. (b) Example of deformation coding using a four-armed GO substrate. Each arm can take one of three states (up = +1, down = –1, flat = 0), allowing up to 3^4 = 81 configurations; here, 27 configurations are shown when arm 1 is fixed in the “+1” state. (c) Reprogramming using stickers magnetized in different orientations, enabling richer deformation modes. (d) Tuning actuation thresholds by varying the magnetization of stickers through different particle concentrations. Sequential

actuation of four arms is demonstrated, with required magnetic fields of 35, 47, 70, and 140 mT to reach a target free-end deflection of 9 mm.

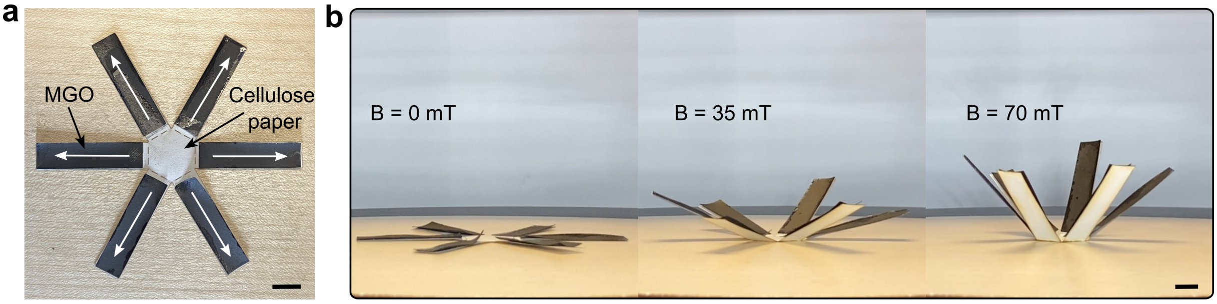

Figure S40. (a) A six-armed structure fabricated from MGO film and cellulose paper, with rectangular MGO strips attached to cellulose paper using PAA. White arrows indicate the direction of magnetization. **(b)** Configurations of MGO/cellulose six-armed structure under different magnetic field magnitudes. When subjected to a uniform magnetic field, the structure shows an "arm-up" configuration with the magnetization direction aligning with the direction of the magnetic field.

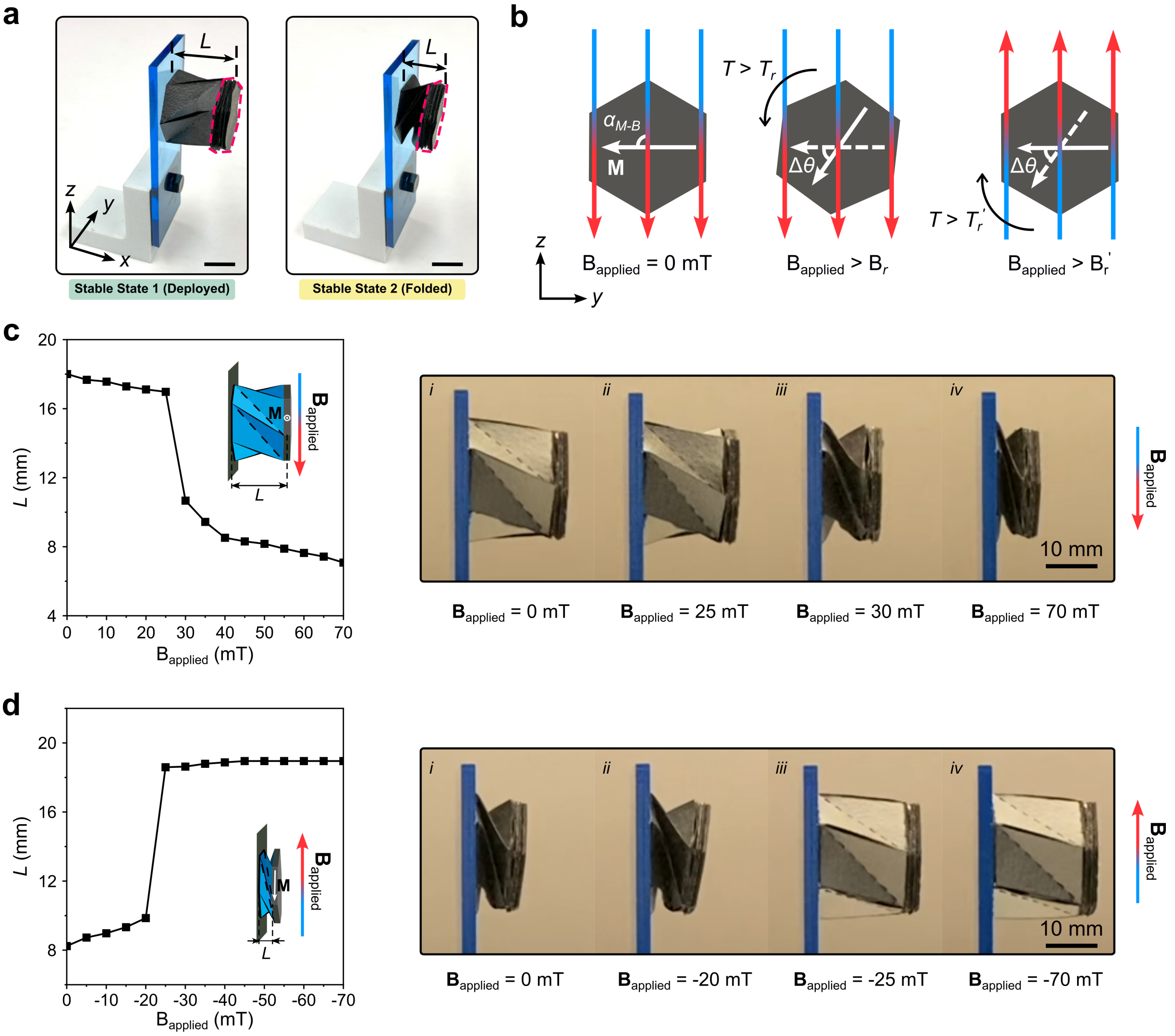

Figure S41. (a) Digital picture of a bistable GO Kresling origami with an assembled MGO magnet composed of multilayered hexagonal MGO panels on the right side (marked by dashed line). The MGO/GO Kresling unit cell exhibits two stable states: deployed and folded. Scale bar: 10 mm. **(b)**

magnetic torque on the assembled MGO magnet. **(c)** Folding and **(d)** deploying of MGO/GO Kresling origami induced by a uniform magnetic field (**B**$_{applied}$). Changes in length (*L*) and corresponding pictures of the MGO/GO Kresling origami under increasing magnitude of applied magnetic field.

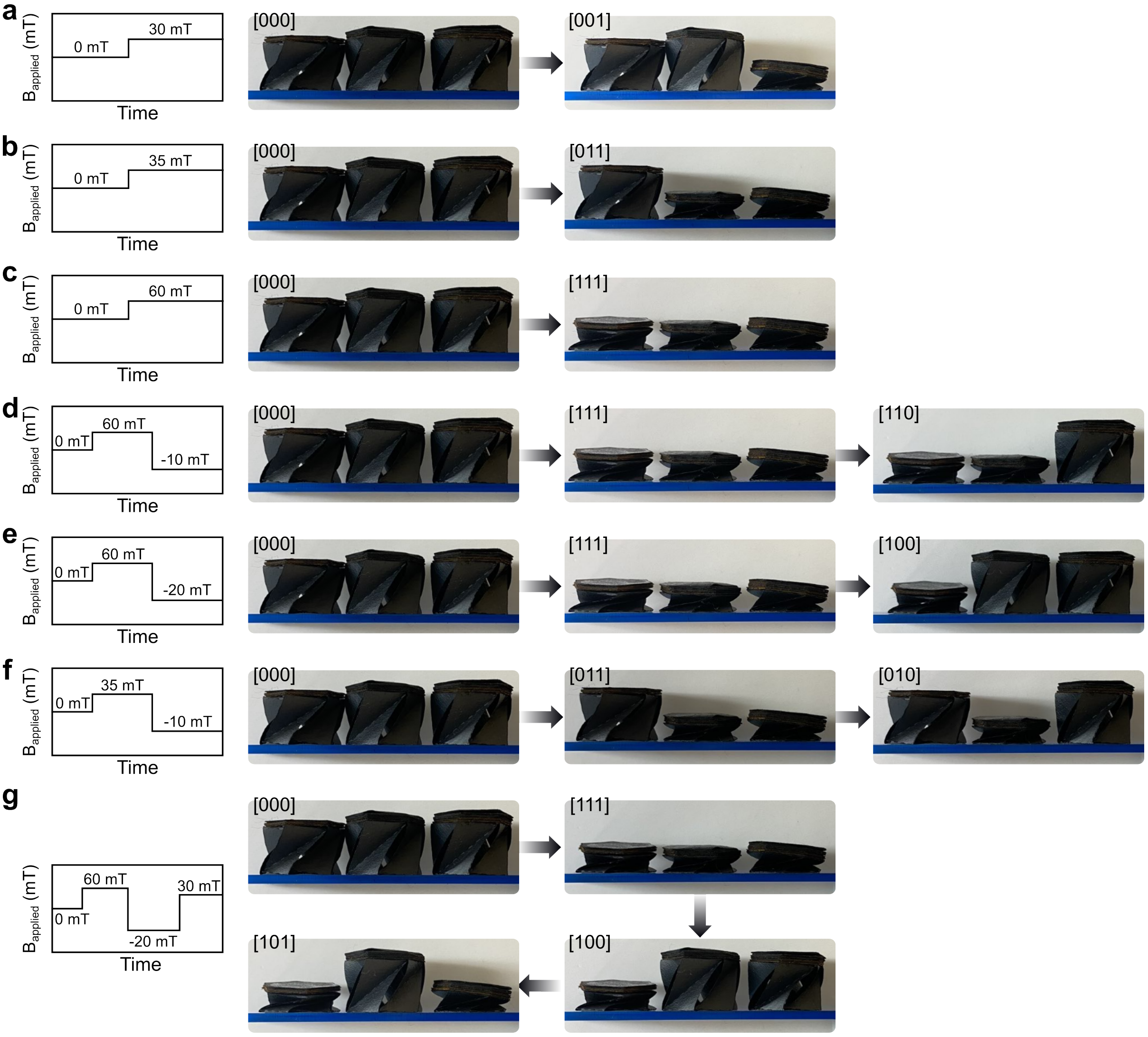

Figure S42. By changing the applied magnetic field, different global mechanologic states can be achieved. In this example, the three MGO/GO Kresling origamis consist of 15, 20, and 25 layers of MGO panels (from left to right, respectively).

S10. Analytical Calculation of Magnetic Actuation

The magneto-elastic behavior of structures composed of MGO films is simulated using finite element analyses in COMSOL Multiphysics 5.5. This involves solving a coupled multi-physical problem that includes both elastic and magnetic domains. The magneto-elastic interaction is modeled through the Maxwell surface stress tensor, satisfying both equilibrium conditions and Maxwell's magnetic equations [113]. This multi-physical problem is computationally expensive to be numerically solved. However, by assuming an ideal hard magnetic material model for the MGO film, the numerical analysis can be greatly simplified [114]. Specifically, we assume that the remanent magnetization remains constant within the range of the actuating magnetic fields and the presence of the hard magnetic material does not perturb the applied actuating magnetic field [65]. This assumption, which is valid only if the actuating magnetic field magnitude is below the material's coercivity, simplifies the general magnetoelastic problem to a single elastic domain, where the magnetization effect of the magnetic domain appears solely as a boundary load per unit area, $\boldsymbol{f}_{mag}$, applied to the bounding surface of the magnetic domain [113]. This boundary load equation can be expressed as follows [114]:

$$\boldsymbol{f}_{\text{mag}} = (J^{-1}\mathbf{F}\boldsymbol{M}) \cdot \hat{\boldsymbol{n}}\, \boldsymbol{B}_{\text{applied}} \quad \text{(Eq. S11)}$$

In this notation, vectors are represented by bold italicized letters, whereas tensors are denoted by bold non-italicized letters: $\boldsymbol{M}$ is the magnetization vector of the magnetic domain, $\boldsymbol{B}_{\text{applied}}$ is the applied magnetic field, $\hat{\boldsymbol{n}}$ is the unit normal to the bounding surface of the magnetized domain, $\mathbf{F}$ is the deformation gradient tensor, and J is the deformation Jacobian, which is equal to the determinant of the deformation gradient. Evidently, $\boldsymbol{f}_{\text{mag}}$ depends on the deformation, the applied magnetic field, and the magnetization; the inclusion of the deformation gradient accounts for the magnetization in the deformed state. We also neglect J by assuming the MGO material to be a nearly incompressible ($J = 1$).

Our finite element analysis is quasi-static with geometric nonlinearity (large deformation) where the elastic domain is modeled using an isotropic linear elastic constitutive equation. We assume a Young's modulus of 5 GPa (Fig. 2d) and a Poisson's ratio of 0.3 for the MGO film. Additionally, we assume a uniform remanent magnetization of 50 kA/m for the MGO film [11]. In the model, we partition the structure into different domains, each with its own distinctive magnetization direction. For each domain, to account for its magnetic load on the structure when subject to an external magnetic field, we define and apply a magnetic surface boundary load $\boldsymbol{f}_{mag}$. Each boundary load is applied to all the boundaries enclosing a magnetic domain. Tetrahedral elements are used with an initial element size set to 0.5 mm. The element size is subsequently updated through one level of adaptive mesh refinement, based on the L2 norm of the squared error, to enhance the accuracy of the results. To validate our FEM simulation, we used the material properties reported in the literature [6] and followed the same procedures as in our previous COMSOL simulations. The results (**Figure S43**) show good agreement, indicating that our FEM simulation is reliable.

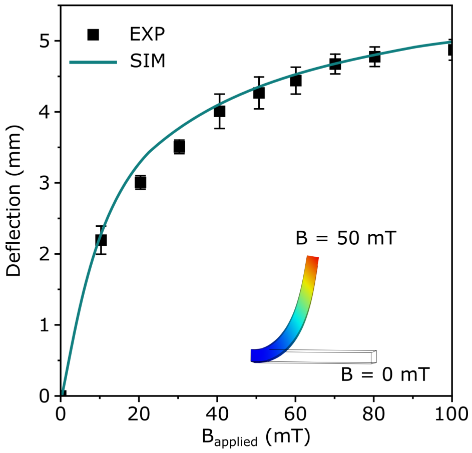

Figure S43. Comparison of FEM and experimental results [6] of the deflection of beam (dimension 5.2 × 3 × 0.5 mm) under the applied magnetic fields.

S11. Supplementary Movies

Movie S1. Stability of MGO film in water.

Movie S2. Magnetically actuated shape morphing of MGO Miura-ori and Miura-ori tube.

Movie S3. Magnetically actuated shape morphing of six-armed MGOs.

Movie S4. Magnetically driven transition between bistable states in MGO Kresling.

Movie S5. Magnetically driven mode transition in MGO-TMP origami.

Movie S6. On-ground locomotion of Miura-ori tube-inspired soft robot.

Movie S7. Bioinspired MGO soft robots.

Movie S8. Programming and reprogramming of magnetization patterns.

Movie S9. Stable state transition in MGO/GO Kresling actuated by Helmholtz coil.

Movie. S10. Multifunctional MGO Kresling for digital computing.

References

[1] Y. Chen, J. Cai, A. Seyedkannai, A. Akbarzadeh, M. Cerruti, Strong and flexible graphene oxide paper for humidity responsive origami metamaterial, Materials Horizons, accepted (2025).
[2] On the praseodymium + oxygen system, Philosophical Transactions of the Royal Society of London. Series A, Mathematical and Physical Sciences 259(1106) (1997) 583-614.
[3] N. Leventis, S. Donthula, C. Mandal, M.S. Ding, C. Sotiriou-Leventis, Explosive versus Thermite Behavior in Iron(0) Aerogels Infiltrated with Perchlorates, Chemistry of Materials 27(23) (2015) 8126-8137.
[4] G.D. Dhamale, V.L. Mathe, S.V. Bhoraskar, S.N. Sahasrabudhe, S. Ghorui, Synthesis and characterization of Nd2O3 nanoparticles in a radiofrequency thermal plasma reactor, Nanotechnology 27(8) (2016) 085603.
[5] P. Duhr, Y.A. Meier, A. Damanpack, J. Carpenter, A.R. Studart, A. Rafsanjani, A.F. Demirors, Kirigami Makes a Soft Magnetic Sheet Crawl, Adv Sci (Weinh) 10(25) (2023) e2301895.
[6] H. Zhu, Y. Wang, Y. Ge, Y. Zhao, C. Jiang, Kirigami-Inspired Programmable Soft Magnetoresponsive Actuators with Versatile Morphing Modes, Adv Sci (Weinh) 9(32) (2022) e2203711.
[7] D. Lin, F. Yang, D. Gong, R. Li, Bio-inspired magnetic-driven folded diaphragm for biomimetic robot, Nat Commun 14(1) (2023) 163.
[8] L. Wang, Y. Chang, S. Wu, R.R. Zhao, W. Chen, Physics-aware differentiable design of magnetically actuated kirigami for shape morphing, Nat Commun 14(1) (2023) 8516.
[9] Q. Ze, S. Wu, J. Dai, S. Leanza, G. Ikeda, P.C. Yang, G. Iaccarino, R.R. Zhao, Spinning-enabled wireless amphibious origami millirobot, Nat Commun 13(1) (2022) 3118.
[10] W. Hu, G.Z. Lum, M. Mastrangeli, M. Sitti, Small-scale soft-bodied robot with multimodal locomotion, Nature 554(7690) (2018) 81-85.
[11] Y. Kim, H. Yuk, R. Zhao, S.A. Chester, X. Zhao, Printing ferromagnetic domains for untethered fast-transforming soft materials, Nature 558(7709) (2018) 274-279.
[12] T. Chen, Programming material logic using magnetically controlled bistability, Proc Natl Acad Sci U S A 120(17) (2023) e2304391120.
[13] L.S. Novelino, Q. Ze, S. Wu, G.H. Paulino, R. Zhao, Untethered control of functional origami microrobots with distributed actuation, Proc Natl Acad Sci U S A 117(39) (2020) 24096-24101.
[14] A. Pal, M. Sitti, Programmable mechanical devices through magnetically tunable bistable elements, Proc Natl Acad Sci U S A 120(15) (2023) e2212489120.
[15] S. Wu, Q. Ze, J. Dai, N. Udipi, G.H. Paulino, R. Zhao, Stretchable origami robotic arm with omnidirectional bending and twisting, Proc Natl Acad Sci U S A 118(36) (2021).
[16] C. Buranabunwong, X. Li, S. Nong, B. Sun, Y. Sun, S. Zhang, M. Li, Magnetic Bistable Dome Actuators for Soft Robotics with High Volume Capacity and Motion Stability, ACS Appl Mater Interfaces 17(2) (2025) 3930-3940.
[17] J. Sim, S. Wu, J. Dai, R.R. Zhao, Magneto-Mechanical Bilayer Metamaterial with Global Area-Preserving Density Tunability for Acoustic Wave Regulation, Adv Mater 35(35) (2023) e2303541.
[18] H. Song, H. Lee, J. Lee, J.K. Choe, S. Lee, J.Y. Yi, S. Park, J.W. Yoo, M.S. Kwon, J. Kim, Reprogrammable Ferromagnetic Domains for Reconfigurable Soft Magnetic Actuators, Nano Lett 20(7) (2020) 5185-5192.
[19] Q. Ze, X. Kuang, S. Wu, J. Wong, S.M. Montgomery, R. Zhang, J.M. Kovitz, F. Yang, H.J. Qi, R. Zhao, Magnetic Shape Memory Polymers with Integrated Multifunctional Shape Manipulation, Adv Mater 32(4) (2020) e1906657.

[20] M. Ha, G.S. Canon Bermudez, J.A. Liu, E.S. Oliveros Mata, B.A. Evans, J.B. Tracy, D. Makarov, Reconfigurable Magnetic Origami Actuators with On-Board Sensing for Guided Assembly, Adv Mater 33(25) (2021) e2008751.
[21] Z. Chen, S. Kong, Y. He, S. Chen, W. Wang, L. Jin, S. Zhang, Y. Hong, L. Pan, H. Wu, Y. Xie, C. Linghu, Z. Mao, Z. Yang, C.H. Chan, J. Song, J. Lu, A Magnet‐Driven Soft Bistable Actuator, Advanced Functional Materials (2024).
[22] X. Wang, G. Mao, J. Ge, M. Drack, G.S. Cañón Bermúdez, D. Wirthl, R. Illing, T. Kosub, L. Bischoff, C. Wang, J. Fassbender, M. Kaltenbrunner, D. Makarov, Untethered and ultrafast soft-bodied robots, Communications Materials 1(1) (2020).
[23] Z. Chen, Y. Wang, H. Chen, J. Law, H. Pu, S. Xie, F. Duan, Y. Sun, N. Liu, J. Yu, A magnetic multi-layer soft robot for on-demand targeted adhesion, Nat Commun 15(1) (2024) 644.
[24] S. Jiang, B. Li, J. Zhao, D. Wu, Y. Zhang, Z. Zhao, Y. Zhang, H. Yu, K. Shao, C. Zhang, R. Li, C. Chen, Z. Shen, J. Hu, B. Dong, L. Zhu, J. Li, L. Wang, J. Chu, Y. Hu, Magnetic Janus origami robot for cross-scale droplet omni-manipulation, Nat Commun 14(1) (2023) 5455.
[25] S. Zhang, X. Ke, Q. Jiang, H. Ding, Z. Wu, Programmable and reprocessable multifunctional elastomeric sheets for soft origami robots, Sci Robot 6(53) (2021).
[26] X. Zhao, H. Yao, Y. Lv, Z. Chen, L. Dong, J. Huang, S. Mi, Reprogrammable Magnetic Soft Actuators with Microfluidic Functional Modules via Pixel-Assembly, Small (2024) e2310009.
[27] L. Li, H. Yao, S. Mi, Magnetically Driven Modular Mechanical Metamaterials with High Programmability, Reconfigurability, and Multiple Applications, ACS Appl Mater Interfaces 15(2) (2023) 3486-3496.
[28] W. Li, M. Sang, C. Lou, G. Liao, S. Liu, J. Wu, X. Gong, Q. Ma, S. Xuan, Triple-Responsive Soft Actuator with Plastically Retentive Deformation and Magnetically Programmable Recovery, ACS Nano 17(23) (2023) 24042-24054.
[29] S.M. Montgomery, S. Wu, X. Kuang, C.D. Armstrong, C. Zemelka, Q. Ze, R. Zhang, R. Zhao, H.J. Qi, Magneto‐Mechanical Metamaterials with Widely Tunable Mechanical Properties and Acoustic Bandgaps, Advanced Functional Materials (2020).
[30] Y. Alapan, A.C. Karacakol, S.N. Guzelhan, I. Isik, M. Sitti, Reprogrammable shape morphing of magnetic soft machines, Sci Adv 6(38) (2020).
[31] X. Liu, Y. Yang, M.E. Inda, S. Lin, J. Wu, Y. Kim, X. Chen, D. Ma, T.K. Lu, X. Zhao, Magnetic Living Hydrogels for Intestinal Localization, Retention, and Diagnosis, Adv Funct Mater 31(27) (2021).
[32] S.R. Goudu, I.C. Yasa, X. Hu, H. Ceylan, W. Hu, M. Sitti, Biodegradable Untethered Magnetic Hydrogel Milli‐Grippers, Advanced Functional Materials 30(50) (2020).
[33] M. Li, Y. Wang, A. Chen, A. Naidu, B.S. Napier, W. Li, C.L. Rodriguez, S.A. Crooker, F.G. Omenetto, Flexible magnetic composites for light-controlled actuation and interfaces, Proc Natl Acad Sci U S A 115(32) (2018) 8119-8124.
[34] Z. Zhang, J.T. Heron, A. Pena‐Francesch, Adaptive Magnetoactive Soft Composites for Modular and Reconfigurable Actuators, Advanced Functional Materials 33(26) (2023).
[35] H. Deng, K. Sattari, Y. Xie, P. Liao, Z. Yan, J. Lin, Laser reprogramming magnetic anisotropy in soft composites for reconfigurable 3D shaping, Nat Commun 11(1) (2020) 6325.
[36] S. Yi, L. Wang, Z. Chen, J. Wang, X. Song, P. Liu, Y. Zhang, Q. Luo, L. Peng, Z. Wu, C.F. Guo, L. Jiang, High-throughput fabrication of soft magneto-origami machines, Nat Commun 13(1) (2022) 4177.
[37] H. Yang, B.S. Yeow, Z. Li, K. Li, T.H. Chang, L. Jing, Y. Li, J.S. Ho, H. Ren, P.Y. Chen, Multifunctional metallic backbones for origami robotics with strain sensing and wireless communication capabilities, Sci Robot 4(33) (2019).

[38] X. Li, Z. Shang, Y. Wang, J. Liu, Y. Xie, J. Li, Y. Liu, W. Gan, Programmable, Changeable, Origami Cellulose Films for Magnetically Controllable Soft Robots, ACS Appl Mater Interfaces 15(23) (2023) 28442-28452.
[39] S. Wu, Q. Ze, R. Zhang, N. Hu, Y. Cheng, F. Yang, R. Zhao, Symmetry-Breaking Actuation Mechanism for Soft Robotics and Active Metamaterials, ACS Appl Mater Interfaces 11(44) (2019) 41649-41658.
[40] Y. Wu, S. Zhang, Y. Yang, Z. Li, Y. Wei, Y. Ji, Locally controllable magnetic soft actuators with reprogrammable contraction-derived motions, Sci Adv 8(25) (2022) eabo6021.
[41] H. Chathuranga, I. Marriam, S. Chen, Z. Zhang, J. MacLeod, Y. Liu, H. Yang, C. Yan, Multistimulus-Responsive Graphene Oxide/Fe(3)O(4)/Starch Soft Actuators, ACS Appl Mater Interfaces 14(14) (2022) 16772-16779.
[42] A. Damnjanovic, I. Milosev, N. Kovacevic, Enhanced mechanical properties and environmental stability of polymer-bonded magnets using three-step surface wet chemical modifications of Nd-Fe-B magnetic powder, Heliyon 10(4) (2024) e26024.
[43] A.-M. Popescu, J. Calderon-Moreno, K. Yanushkevish, A. Aplevich, O. Demidenko, E.I. Neacsu, V. Constantin, Corrosion Behavior of NdFeB Magnets in Different Aqueous Solutions, Journal of the Brazilian Chemical Society (2024).
[44] M.P. Paranthaman, V. Yildirim, T.N. Lamichhane, B.A. Begley, B.K. Post, A.A. Hassen, B.C. Sales, K. Gandha, I.C. Nlebedim, Additive Manufacturing of Isotropic NdFeB PPS Bonded Permanent Magnets, Materials (Basel) 13(15) (2020).
[45] Y.W. Song, H. Zhang, H.X. Yang, Z.L. Song, A comparative study on the corrosion behavior of NdFeB magnets in different electrolyte solutions, Materials and Corrosion 59(10) (2008) 794-801.
[46] K. Shimba, M. Yamazaki, T. Horikawa, S. Sugimoto, H. Mitarai, Effect of Phosphate Treatment on the Corrosion Resistance of Nd–Fe–B Anisotropic Magnetic Powder, IEEE Transactions on Magnetics 59(11) (2023) 1-4.
[47] K. Gandha, M.P. Paranthaman, H. Wang, X. Liu, I.C. Nlebedim, Thermal stability of anisotropic bonded magnets prepared by additive manufacturing, Journal of the American Ceramic Society 106(1) (2022) 166-171.
[48] H.F. Webster, J.P. Wightman, Effects of oxygen and ammonia plasma treatment on polyphenylene sulfide thin films and their interaction with epoxy adhesive, Journal of Adhesion Science and Technology 5(1) (1991) 93-106.
[49] Y. Yang, L. Yang, Y. Sun, N. Jiang, C. Guan, X. Fang, J. Liu, Preparation and corrosion resistance of epoxy resin coating for bonded NdFeB magnet, Progress in Organic Coatings 173 (2022).
[50] Y. Yang, Y. Sun, L. Yang, L. Su, M. Jia, Y. Chen, X. Fang, J. Liu, Preparation and Anticorrosion Performance of Double-Layer Epoxy Resin Coatings on Bonded NdFeB Magnets, Journal of Materials Engineering and Performance 34(1) (2023) 473-483.
[51] Y. Yang, Y. Wang, L. Yang, R. Ren, Y. Chen, B. Dong, J. Liu, X. Fang, Q. Gao, The Preparation and Properties of ZnAl Coating for Ring-Shaped Bonded NdFeB Magnet with High Corrosion Resistance, Journal of Materials Engineering and Performance 31(2) (2021) 1003-1008.
[52] Y. Gao, Y. Bai, H. Zhu, W. Liang, Q. Liu, H. Dong, R. Jia, W. Ma, Corrosion Resistance, Mechanical and Magnetic Properties of Cold-Sprayed Al Coating on Sintered NdFeB Magnet, Journal of Thermal Spray Technology 30(8) (2021) 2117-2127.
[53] Y. Yang, N. Jiang, Y. Sun, L. Yang, C. Guan, E. Zhang, X. Fang, J. Liu, Structure and Corrosion Resistance Characteristics of ZnAl/EP Coating on Bonded NdFeB Magnet, Journal of Materials Engineering and Performance 32(12) (2022) 5475-5482.
[54] C. Lv, D. Krishnaraju, G. Konjevod, H. Yu, H. Jiang, Origami based mechanical metamaterials, Sci Rep 4 (2014) 5979.

[55] Z. Lin, L.S. Novelino, H. Wei, N.A. Alderete, G.H. Paulino, H.D. Espinosa, S. Krishnaswamy, Folding at the Microscale: Enabling Multifunctional 3D Origami-Architected Metamaterials, Small (2020) e2002229.
[56] E.T. Filipov, T. Tachi, G.H. Paulino, Origami tubes assembled into stiff, yet reconfigurable structures and metamaterials, Proc Natl Acad Sci U S A 112(40) (2015) 12321-6.
[57] M. Schenk, S.D. Guest, Geometry of Miura-folded metamaterials, Proc Natl Acad Sci U S A 110(9) (2013) 3276-81.
[58] Z.Y. Wei, Z.V. Guo, L. Dudte, H.Y. Liang, L. Mahadevan, Geometric mechanics of periodic pleated origami, Phys Rev Lett 110(21) (2013) 215501.
[59] L.T. Gaeta, K.J. McDonald, L. Kinnicutt, M. Le, S. Wilkinson-Flicker, Y. Jiang, T. Atakuru, E. Samur, T. Ranzani, Magnetically induced stiffening for soft robotics, Soft Matter 19(14) (2023) 2623-2636.
[60] X. Zhang, Z. Wu, L.a. Jin, J. Yang, X. Ou, D. Ni, Y. Cheng, L. Zhao, Y. Tong, W. Dong, B. Wu, G. Li, Q. Yao, Portable Magnetic Field Mapping Measurement System Based on Large-Scale Dipole Magnets in HIAF, Metrology 5(2) (2025).
[61] L. Rondin, J.P. Tetienne, P. Spinicelli, C. Dal Savio, K. Karrai, G. Dantelle, A. Thiaville, S. Rohart, J.F. Roch, V. Jacques, Nanoscale magnetic field mapping with a single spin scanning probe magnetometer, Applied Physics Letters 100(15) (2012).
[62] F. Niu, Q. Xue, Q. Cao, X. He, T. Wang, H. Wang, C. Hao, X. Li, Y. Li, H. Yang, H. Yang, D. Han, Magneto-soft robots based on multi-materials optimizing and heat-assisted in-situ magnetic domains programming, International Journal of Extreme Manufacturing 7(5) (2025).
[63] D. Yan, A. Abbasi, P.M. Reis, A comprehensive framework for hard-magnetic beams: Reduced-order theory, 3D simulations, and experiments, International Journal of Solids and Structures 257 (2022).
[64] S.I. Bernad, E. Bernad, Magnetic Forces by Permanent Magnets to Manipulate Magnetoresponsive Particles in Drug-Targeting Applications, Micromachines (Basel) 13(11) (2022).
[65] Y. Kim, X. Zhao, Magnetic Soft Materials and Robots, Chem Rev 122(5) (2022) 5317-5364.
[66] M. Mooney, The viscosity of a concentrated suspension of spherical particles, Journal of Colloid Science 6(2) (1951) 162-170.
[67] S.-Y. Fu, X.-Q. Feng, B. Lauke, Y.-W. Mai, Effects of particle size, particle/matrix interface adhesion and particle loading on mechanical properties of particulate–polymer composites, Composites Part B: Engineering 39(6) (2008) 933-961.
[68] C.S.X. Ng, M.W.M. Tan, C. Xu, Z. Yang, P.S. Lee, G.Z. Lum, Locomotion of Miniature Soft Robots, Adv Mater 33(19) (2021) e2003558.
[69] Y. Lee, F. Koehler, T. Dillon, G. Loke, Y. Kim, J. Marion, M.J. Antonini, I. Garwood, A. Sahasrabudhe, K. Nagao, X. Zhao, Y. Fink, E.T. Roche, P. Anikeeva, Magnetically Actuated Fiber-Based Soft Robots, Adv Mater (2023) e2301916.
[70] N.V. Medhekar, A. Ramasubramaniam, R.S. Ruoff, V.B. Shenoy, Hydrogen bond networks in graphene oxide composite paper: structure and mechanical properties, ACS Nano 4(4) (2010) 2300-6.
[71] S. Plimpton, Fast Parallel Algorithms for Short-Range Molecular Dynamics, Journal of Computational Physics 117(1) (1995) 1-19.
[72] A. Stukowski, Visualization and analysis of atomistic simulation data with OVITO–the Open Visualization Tool, Modelling and Simulation in Materials Science and Engineering 18(1) (2010).
[73] K. Chenoweth, A.C. van Duin, W.A. Goddard, 3rd, ReaxFF reactive force field for molecular dynamics simulations of hydrocarbon oxidation, J Phys Chem A 112(5) (2008) 1040-53.

[74] A.C.T. van Duin, S. Dasgupta, F. Lorant, W.A. Goddard, ReaxFF: A Reactive Force Field for Hydrocarbons, The Journal of Physical Chemistry A 105(41) (2001) 9396-9409.
[75] C.-T. Chen, F.J. Martin-Martinez, S. Ling, Z. Qin, M.J. Buehler, Nacre-inspired design of graphene oxide–polydopamine nanocomposites for enhanced mechanical properties and multi-functionalities, Nano Futures 1(1) (2017).
[76] D. Hou, T. Yang, A reactive molecular dynamics study of graphene oxide sheets in different saturated states: structure, reactivity and mechanical properties, Phys Chem Chem Phys 20(16) (2018) 11053-11066.
[77] O.C. Compton, S.W. Cranford, K.W. Putz, Z. An, L.C. Brinson, M.J. Buehler, S.T. Nguyen, Tuning the mechanical properties of graphene oxide paper and its associated polymer nanocomposites by controlling cooperative intersheet hydrogen bonding, ACS Nano 6(3) (2012) 2008-19.
[78] F. Guo, Y. Wang, Y. Jiang, Z. Li, Z. Xu, X. Zhao, T. Guo, W. Jiang, C. Gao, Hydroplastic Micromolding of 2D Sheets, Adv Mater 33(25) (2021) e2008116.
[79] Z. Zhai, L. Wu, H. Jiang, Mechanical metamaterials based on origami and kirigami, Applied Physics Reviews 8(4) (2021).
[80] Z. Ding, C. Yuan, X. Peng, T. Wang, H.J. Qi, M.L. Dunn, Direct 4D printing via active composite materials, Sci Adv 3(4) (2017) e1602890.
[81] J. Kim, J.A. Hanna, M. Byun, C.D. Santangelo, R.C. Hayward, Designing responsive buckled surfaces by halftone gel lithography, Science 335(6073) (2012) 1201-5.
[82] Q. Zhang, K. Zhang, G. Hu, Smart three-dimensional lightweight structure triggered from a thin composite sheet via 3D printing technique, Sci Rep 6 (2016) 22431.
[83] J. Wu, C. Yuan, Z. Ding, M. Isakov, Y. Mao, T. Wang, M.L. Dunn, H.J. Qi, Multi-shape active composites by 3D printing of digital shape memory polymers, Sci Rep 6 (2016) 24224.
[84] Q. Ge, A.H. Sakhaei, H. Lee, C.K. Dunn, N.X. Fang, M.L. Dunn, Multimaterial 4D Printing with Tailorable Shape Memory Polymers, Sci Rep 6 (2016) 31110.
[85] A.S. Gladman, E.A. Matsumoto, R.G. Nuzzo, L. Mahadevan, J.A. Lewis, Biomimetic 4D printing, Nat Mater 15(4) (2016) 413-8.
[86] S.E. Bakarich, R. Gorkin, 3rd, M. in het Panhuis, G.M. Spinks, 4D Printing with Mechanically Robust, Thermally Actuating Hydrogels, Macromol Rapid Commun 36(12) (2015) 1211-7.
[87] A. Kotikian, R.L. Truby, J.W. Boley, T.J. White, J.A. Lewis, 3D Printing of Liquid Crystal Elastomeric Actuators with Spatially Programed Nematic Order, Adv Mater 30(10) (2018).
[88] L. Huang, R. Jiang, J. Wu, J. Song, H. Bai, B. Li, Q. Zhao, T. Xie, Ultrafast Digital Printing toward 4D Shape Changing Materials, Adv Mater 29(7) (2017).
[89] W. Li, F. Li, H. Li, M. Su, M. Gao, Y. Li, D. Su, X. Zhang, Y. Song, Flexible Circuits and Soft Actuators by Printing Assembly of Graphene, ACS Appl Mater Interfaces 8(19) (2016) 12369-76.
[90] C.P. Ambulo, J.J. Burroughs, J.M. Boothby, H. Kim, M.R. Shankar, T.H. Ware, Four-dimensional Printing of Liquid Crystal Elastomers, ACS Appl Mater Interfaces 9(42) (2017) 37332-37339.
[91] Y. Wang, H. Ye, J. He, Q. Ge, Y. Xiong, Electrothermally controlled origami fabricated by 4D printing of continuous fiber-reinforced composites, Nat Commun 15(1) (2024) 2322.
[92] Y.-L. Zhang, J.-N. Ma, S. Liu, D.-D. Han, Y.-Q. Liu, Z.-D. Chen, J.-W. Mao, H.-B. Sun, A “Yin”-“Yang” complementarity strategy for design and fabrication of dual-responsive bimorph actuators, Nano Energy 68 (2020).
[93] J. Lin, P. Zhou, Z. Wen, W. Zhang, Z. Luo, L. Chen, Chinese ink: a programmable, dual-responsive and self-sensing actuator using a healing-assembling method, Nanoscale 13(47) (2021) 20134-20143.

[94] D. Gao, M.F. Lin, J. Xiong, S. Li, S.N. Lou, Y. Liu, J.H. Ciou, X. Zhou, P.S. Lee, Photothermal actuated origamis based on graphene oxide-cellulose programmable bilayers, Nanoscale Horiz 5(4) (2020) 730-738.
[95] Y. Dong, J. Wang, X. Guo, S. Yang, M.O. Ozen, P. Chen, X. Liu, W. Du, F. Xiao, U. Demirci, B.F. Liu, Multi-stimuli-responsive programmable biomimetic actuator, Nat Commun 10(1) (2019) 4087.
[96] J. Mu, C. Hou, H. Wang, Y. Li, Q. Zhang, M. Zhu, Origami-inspired active graphene-based paper for programmable instant self-folding walking devices, Sci Adv 1(10) (2015) e1500533.
[97] N. Nayakanti, S.H. Tawfick, A.J. Hart, Twist-coupled Kirigami cells and mechanisms, Extreme Mechanics Letters 21 (2018) 17-24.
[98] R.J. Lang, Twists, Tilings, and Tessellations: Mathematical Methods for Geometric Origami, CRC Press2017.
[99] H. Yasuda, B. Gopalarethinam, T. Kunimine, T. Tachi, J. Yang, Origami ‐ Based Cellular Structures with In Situ Transition between Collapsible and Load ‐ Bearing Configurations, Advanced Engineering Materials 21(12) (2019).
[100] Y. Miyazawa, H. Yasuda, H. Kim, J.H. Lynch, K. Tsujikawa, T. Kunimine, J.R. Raney, J. Yang, Heterogeneous origami-architected materials with variable stiffness, Communications Materials 2(1) (2021).
[101] A.A. Deleo, J. O'Neil, H. Yasuda, M. Salviato, J. Yang, Origami-based deployable structures made of carbon fiber reinforced polymer composites, Composites Science and Technology 191 (2020).
[102] H. Yasuda, J. Yang, Reentrant Origami-Based Metamaterials with Negative Poisson's Ratio and Bistability, Phys Rev Lett 114(18) (2015) 185502.
[103] H. Yasuda, T. Yein, T. Tachi, K. Miura, M. Taya, Folding behaviour of Tachi-Miura polyhedron bellows, Proc Math Phys Eng Sci 469(2159) (2013) 20130351.
[104] W. Wang, J.Y. Lee, H. Rodrigue, S.H. Song, W.S. Chu, S.H. Ahn, Locomotion of inchworm-inspired robot made of smart soft composite (SSC), Bioinspir Biomim 9(4) (2014) 046006.
[105] K.E. Feitl, A.F. Millett, S.P. Colin, J.O. Dabiri, J.H. Costello, Functional morphology and fluid interactions during early development of the scyphomedusa Aurelia aurita, Biol Bull 217(3) (2009) 283-91.
[106] Z. Ren, W. Hu, X. Dong, M. Sitti, Multi-functional soft-bodied jellyfish-like swimming, Nat Commun 10(1) (2019) 2703.
[107] X. Kuang, S. Wu, Q. Ze, L. Yue, Y. Jin, S.M. Montgomery, F. Yang, H.J. Qi, R. Zhao, Magnetic Dynamic Polymers for Modular Assembling and Reconfigurable Morphing Architectures, Adv Mater 33(30) (2021) e2102113.
[108] L. Wang, M.Y. Razzaq, T. Rudolph, M. Heuchel, U. Nöchel, U. Mansfeld, Y. Jiang, O.E.C. Gould, M. Behl, K. Kratz, A. Lendlein, Reprogrammable, magnetically controlled polymeric nanocomposite actuators, Materials Horizons 5(5) (2018) 861-867.
[109] Y. Sun, H. Zhang, Y. Zhao, J. Wu, Y. Zhu, M. Li, L. Wang, Locally Reprogrammable Magnetic Micropillars with On ‐ Demand Reconfiguration and Multi ‐ Functionality, Advanced Materials Technologies 8(22) (2023).
[110] M.R.B. Mermet-Guyennet, J. Gianfelice de Castro, H.S. Varol, M. Habibi, B. Hosseinkhani, N. Martzel, R. Sprik, M.M. Denn, A. Zaccone, S.H. Parekh, D. Bonn, Size-dependent reinforcement of composite rubbers, Polymer 73 (2015) 170-173.
[111] M. Mermet-Guyennet, M. Dinkgreve, M. Habibi, N. Martzel, R. Sprik, M. Denn, D. Bonn, Dependence of nonlinear elasticity on filler size in composite polymer systems, Rheologica Acta 56(6) (2017) 583-589.

[112] E. Diller, C. Pawashe, S. Floyd, M. Sitti, Assembly and disassembly of magnetic mobile micro-robots towards deterministic 2-D reconfigurable micro-systems, The International Journal of Robotics Research 30(14) (2011) 1667-1680.
[113] L. Dorfmann, R.W. Ogden, Nonlinear Theory of Electroelastic and Magnetoelastic Interactions, Springer New York, NY, New York, 2014.
[114] R. Zhao, Y. Kim, S.A. Chester, P. Sharma, X. Zhao, Mechanics of hard-magnetic soft materials, Journal of the Mechanics and Physics of Solids 124 (2019) 244-263.
